# Unlocking Therapeutic Potential of Novel Thieno-Oxazepine Hybrids as Multi-Target Inhibitors of AChE/BChE and Evaluation Against Alzheimer’s Disease: In Vivo, In Vitro, Histopathological, and Docking Studies

**DOI:** 10.3390/ph18081214

**Published:** 2025-08-17

**Authors:** Khulood H. Oudah, Mazin A. A. Najm, Triveena M. Ramsis, Maha A. Ebrahim, Nirvana A. Gohar, Karema Abu-Elfotuh, Ehsan Khedre Mohamed, Ahmed M. E. Hamdan, Amira M. Hamdan, Reema Almotairi, Shaimaa R. Abdelmohsen, Khaled Ragab Abdelhakim, Abdou Mohammed Ahmed Elsharkawy, Eman A. Fayed

**Affiliations:** 1 Department of Pharmacy, Mazaya University College, Nasiriyah 64001, Iraq; khuloodhayal@gmail.com (K.H.O.); mazinsajad@gmail.com (M.A.A.N.); 2 Department of Pharmaceutical Chemistry, Faculty of Pharmacy, Sinai University, Kantara Branch, Ismailia 41636, Egypt; triveena.farid@su.edu.eg; 3 Department of Pharmaceutical Organic Chemistry, Faculty of Pharmacy (Girls), Al-Azhar University, Cairo 11754, Egypt; maha.ahmed@azhar.edu.eg; 4 Department of Pharmaceutical Organic Chemistry, Faculty of Pharmacy, Modern University for Technology and Information (MTI), Cairo 11571, Egypt; nirvana.goher@pharm.mti.edu.eg; 5 Department of Clinical Pharmacy, Faculty of Pharmacy (Girls), Al-Azhar University, Cairo 11754, Egypt; karimasoliman.pharmg@azhar.edu.eg; 6 College of Pharmacy, Al-Ayen Iraqi University (AUIQ), An Nasiriyah 64004, Iraq; 7 Department of Biochemistry, Egyptian Drug Authority (EDA), Formerly National Organization of Drug Control and Research (NODCAR), Giza 12654, Egypt; drehsankhedre@hotmail.com; 8 Department of Pharmacy Practice, Faculty of Pharmacy, University of Tabuk, Tabuk 71491, Saudi Arabia; a_hamdan@ut.edu.sa; 9 Oceanography Department, Faculty of Science, Alexandria University, Alexandria 21515, Egypt; amirahamdan@alexu.edu.eg; 10 Department of Medical Laboratory Technology, Faculty of Applied Medical Sciences, University of Tabuk, Tabuk 71491, Saudi Arabia; ralmotairi@ut.edu.sa; 11 Department of Anatomy and Embryology, Faculty of Medicine, Al-Azhar University, Cairo 11754, Egypt; shaimaaramadan.medg@azhar.edu.eg; 12 Department of Histology, Misr University for Science and Technology, Giza 12566, Egypt; khalid.ragab@must.edu.eg; 13 Anatomy Department, Faculty of Medicine, Al-Azhar University, Cairo 11754, Egypt; abdouelsharkawy@gmail.com

**Keywords:** bicyclic, tricyclic, CNS, multi-target, histopathological examination, AChE, BChE, anti-inflammatory biomarkers, antioxidant biomarkers, in silico ADMET, docking studies

## Abstract

**Background:** Alzheimer’s disease (AD) is largely linked with oxidative stress, the accumulation of amyloid-*β* plaques, and hyperphosphorylated *τ*-protein aggregation. Alterations in dopaminergic and serotonergic neurotransmission have also been implicated in various AD-related symptoms. **Methods**: To explore new therapeutic agents, a series of bicyclic and tricyclic thieno-oxazepine derivatives were synthesized as potential acetylcholinesterase (AChE) and butyrylcholinesterase (BChE) inhibitors. The resultant compounds were purified via HPLC and characterized using spectral analysis techniques. Histopathological examinations, other antioxidants, and anti-inflammatory biomarkers were evaluated, and in silico ADMET calculations were performed for synthetic hybrids. Molecular docking was utilized to validate the new drugs’ binding mechanisms. **Results**: The most powerful AChE inhibitors were **14** and **16**, with respective values of IC_50_ equal to 0.39 and 0.76 µM. Derivative **15** demonstrated remarkable BChE-inhibitory efficacy, on par with tacrine, with IC_50_ values of 0.70 µM. Hybrids **13** and **15** showed greater selectivity towards BChE, despite substantial inhibition of AChE. Compounds **13** and **15** reduced escape latency and raised residence time, with almost equal activity to donepezil. **Conclusions:** According to these findings, the designed hybrids constitute multipotent lead compounds that could be used in the creation of novel anti-AD medications.

## 1. Introduction

Cholinergic and glutamatergic impairments are connected to a variety of mechanisms that contribute to cognitive loss in Alzheimer’s disease, a relatively complicated neurodegenerative illness. These elements include psychosis, dissatisfaction or violence, mood disorders (such as anxiety and depression), and deficiencies in facial expressions and emotional communication [1,2]. Previous biochemical studies have identified oxidative stress, the accumulation of hyperphosphorylated *τ*-protein, and amyloid-*β* (A*β*) formation as the key pathology of Alzheimer’s disease (AD) [3,4,5,6]. A decline in acetylcholine (ACh) levels within brain areas, accountable for memory and learning, results in the degeneration of cholinergic neurons, further driving the progression of AD [7,8,9,10,11,12]. ACh is increased in the synaptic cleft by AChE inhibitors, which prevent its breakdown [13,14,15,16,17,18,19].

**Figure 1:**
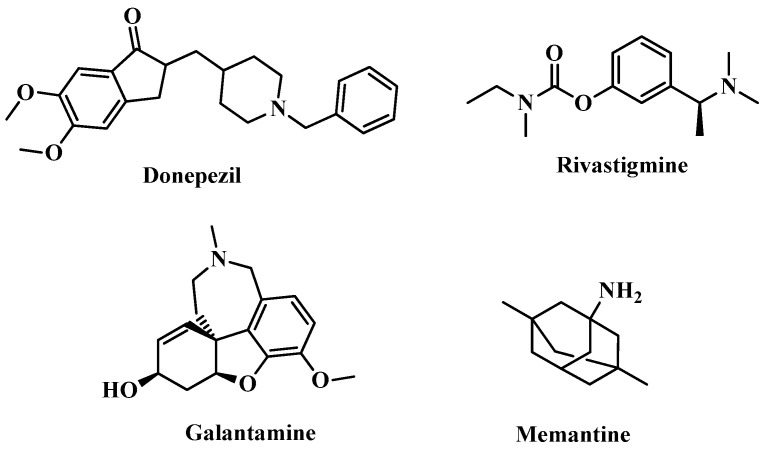
Anti-AD FDA-approved drugs.

Apoptotic cascades are regulated by the Bcl-2 family; although BAX stimulates apoptosis, Bcl-2 suppresses it [20,21,22,23,24]. A substantial change in the ratio of BAX (pro-apoptotic) to Bcl-2 (anti-apoptotic) is brought about by the overexpression of BAX and the decrease in Bcl-2 gene expression in AD [25,26,27,28,29]. The diffuse distribution of A*β* throughout the brain parenchyma causes oxidative stress, neuroinflammation, and neuronal apoptosis/necrosis [30,31,32]. Neurodegeneration is triggered by the stimulation of the NLRP3 inflammasome complex, which is initiated by an inflammatory cascade following the interaction of amyloid-*β* (Aβ) with Toll-like receptor 4 (TLR4) [33,34,35,36,37,38,39]. Treatment approaches that suppress GSK3*β* and/or increase Wnt/*β*-catenin signaling in neural cells can reduce the negative impact of A*β* and help neurogenesis in AD patients [40,41,42]. AChE inhibitor medications, such as donepezil, [43] rivastigmine, [44] memantine, [45] and galantamine, [46] are used to manage AD (Figure 1) [47].

Growing evidence suggests that BChE plays a critical role in regulating acetylcholine (ACh) in the brain throughout the progression of AD [48,49,50]. In advanced phases of AD, brain levels of BChE can increase to approximately 165% of normal, whereas AChE levels decline to about 55–67% of typical values [51]. Notably, BChE inhibition produces no adverse consequences. The creation of selective and powerful BChE inhibitors, which may increase ACh levels in the brain while drastically reducing adverse effects on the peripheral nervous system, represents a significant advancement in this regard [52,53,54,55].

Due to their abundant natural occurrence, heterocyclic compounds are used extensively in the domains of biology and synthesis [56,57,58,59,60]. The heterocyclic sulfur-containing moiety, thiophene, exhibits specific biological characteristics, including cytotoxic, antiviral, antibacterial, and antifungal effects [61,62,63,64,65,66,67]. Similarly, aryl-fused 1,4-oxazepines are structural elements of biologically active compounds that are utilized in medications such as analgesics, antihistaminics, psychoneurotics, and for the management of AD-related memory impairment [68,69].

As a therapeutic method to slow the progression of AD against AlCl_3_-induced AD, we implemented a rat model to investigate the impact of our newly synthesized compounds on behavioral parameters. This is because the pathological process of AD involves multiple mechanisms, all of which lead to increased inflammation and oxidative stress.

**Figure 2:**
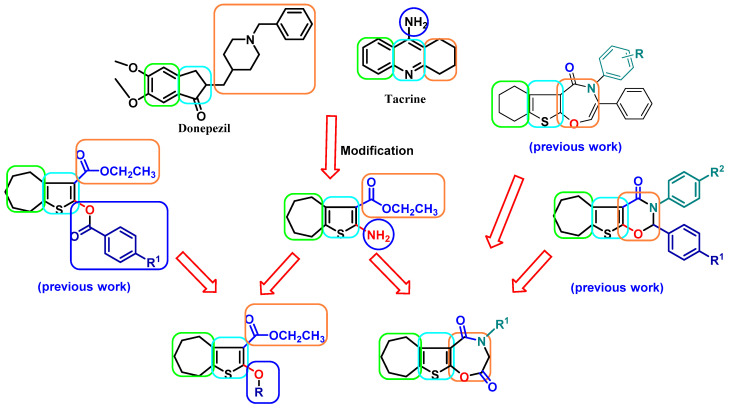
Reasoned analysis of the recently created synthetic materials.

As an extension of our previous work on the creation of cyclohepta[*b*]thiophene-containing compounds, the aim of this article is to create new lead compounds that include cyclohepta-thieno-oxazepine hybrids that could be applied as AChE and BChE inhibitors to treat AD [70,71,72]. To discover novel candidates with dual inhibitory activity against AChE and BChE, we synthesized a new cyclohepta[*b*]thiophene derivatives which are bicyclic and tricyclic (Figure 2). These compounds were assessed for their effects on multiple pathways, including cognitive function (learning and memory), antioxidant capacity, suppression of neuroinflammation, reduction in β-amyloid accumulation, modulation of apoptotic markers, improvement of brain-derived neurotrophic factor (BDNF) levels, and regulation of BAX/Bcl-2 and Wnt/*β*-catenin signaling paths. The modes of action of inhibitors of AChE and BChE were also identified among the most effective hybrids. Additional lead optimization may be aided by the outcomes of the accomplished docking studies and the ADMET property predictions.

## 2. Results and Discussion

### 2.1. Chemistry

**Scheme 1:**
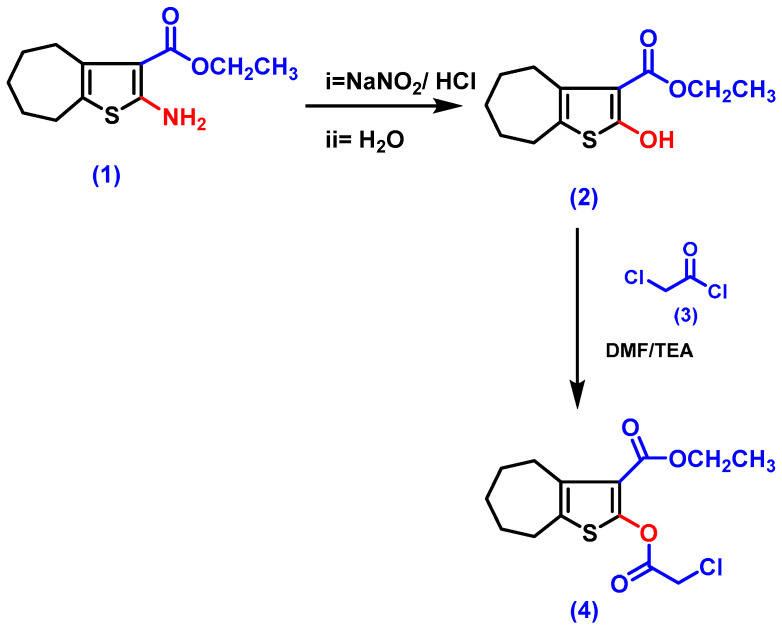
Synthesis of cyclohepta[*b*]thienophen derivatives **2** and **4**.

**Scheme 2:**
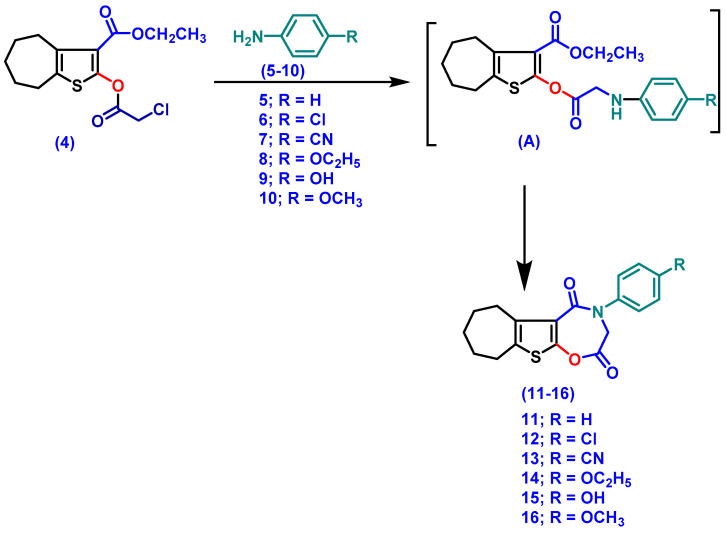
Formation of hybrids of cyclohepta[*b*]thieno-oxazepine **11**–**16**.

The study discusses newly made bi- and tricyclic cyclohepta[*b*]thiophene-oxazepine hybrids, shown in Scheme 1 and Scheme 2.

The target hybrids were synthesized following the outlined preparations. Derivative **1** was produced via the Gewald reaction [73]. Compound **2** was synthesized from **1** as reported [74].

Ester **4** was synthesized by reacting the 2-hydroxy thiophene derivative **2** with chloroacetylchloride [75,76].

The ^1^H NMR spectrum of hybrid **4** represented a quartet at *δ* 4.35 ppm due to 2Hs of -CH_2_-CH_3_) and a singlet at *δ* 4.25 ppm corresponding to 2Hs of CO-CH_2_-Cl, while the ^13^C NMR spectrum showed a signal at *δ* 166 ppm, indicating (CO-CH_2_-Cl), and another one at *δ* 163 ppm that indicates (CO-OCH_2_CH_3_).

Different aniline derivatives were allowed to react with thieno-chloroacetate **4**, resulting in 1,4-oxazepine-2,5-diones **9**–**14** [77,78]. The structure of **11** was evidenced by the ^1^H NMR, which displayed a singlet at *δ* 4.7 ppm matching with 2Hs of oxazepine CH_2_ and multiplets at *δ* 7.0–7.3 ppm, indicating phenyl protons.

Furthermore, the ^1^H NMR spectrum of hybrid **12** demonstrated two doublets of the phenyl ring at *δ* 7.35 and 7.28 ppm and a singlet at *δ* 4.7 ppm due to the 2Hs of oxazepine CH_2_.

The ^1^H NMR of compound **13** revealed two doublets at *δ* 7.5 and 7.5 ppm relative to the *para*-substituted phenyl hydrogens and a singlet at *δ* 4.7 due to oxazine protons, while the ^13^C NMR spectrum displayed two signals at *δ* 47.0 and 118.0 ppm that refer to oxazine-CH and CN, respectively.

The ^1^H NMR spectrum of compound **14** displayed a singlet at *δ* 4.7 ppm, corresponding to oxazine protons; a quartet at *δ* 4.0 ppm, owing to -CH_2_-CH_3_; and a triplet at *δ* 2.8 ppm due to CH_2_-CH_3_. Meanwhile, the ^13^C NMR spectrum showed a signal at *δ* 47.00 ppm, which refers to oxazine CH_2_, and signals at 63.6 and 14.6 ppm, indicating -OCH_2_CH_3_ carbons.

The ^1^H NMR spectrum of derivative **15** displayed a singlet at *δ* 7.75 ppm due to the OH proton, a doublet of doublet signals at *δ* 7.21 and 6.80 ppm due to the *para*-substituted phenyl Hs, and a singlet signal at *δ* 4.77 ppm due to the oxazine protons.

The ^1^H NMR spectra of derivative **16** revealed a singlet signal at *δ* 3.79 ppm due to the OCH_3_ proton, a doublet of doublet signals at *δ* 7.29 and 6.87 ppm due to the *para*-substituted phenyl hydrogens, and a singlet signal at *δ* 4.77 ppm due to the oxazine hydrogens. However, the ^13^C NMR spectrum showed two carbonyl carbon signals at *δ* 164 and 163 ppm and a singlet at *δ* 47.00 ppm, referring to oxazine CH_2_, and a signal at *δ* 54.30, corresponding to OCH_3_ carbon.

### 2.2. Biology

#### 2.2.1. Impact on Behavioral Assessments in AlCl_3_-Induced AD

**Figure 3:**
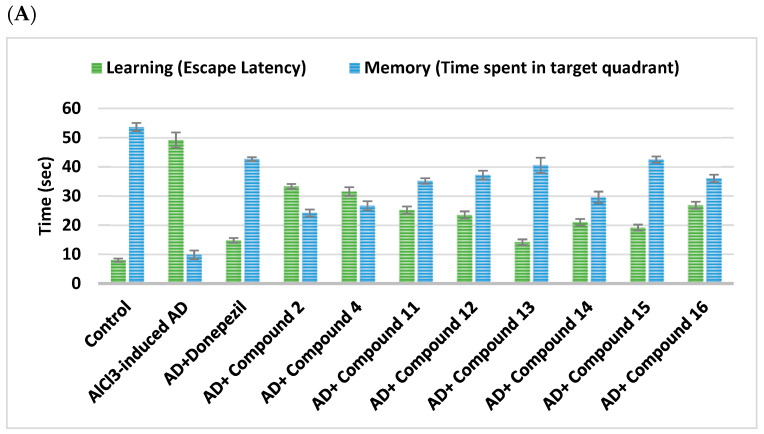

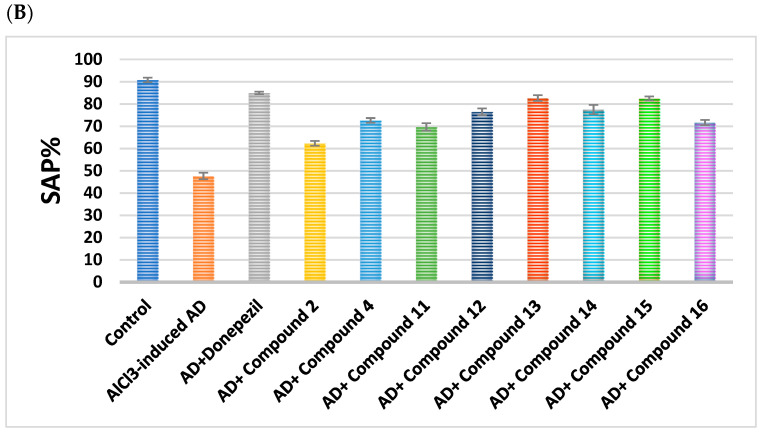
Impact on (**A**) escape latency/time spent in target quadrant and (**B**) Y-maze SAP%. Data is offered as means ± SE (n = 6).

Donepezil served as a control medication for all behavioral tests (Figure 3). While compounds **11**, **12**, **14**, and **16** produced moderate decreases in escape latency that fluctuated between 57.3% and 45.4%, compounds **13** and **15** displayed significant reductions in escape latency of 71.2% and 61%, respectively. Mild escape latency reductions of 32.2% and 35.9% were seen in hybrids **2** and **4**, respectively.

While hybrids **11**, **12**, and **16** demonstrated a modest increase in quadrant resident duration by 3.5, 3.7, and 3.6 times, respectively, the delivery of hybrids **13** and **15** raised the residence time by about 4.1 and 4.3 times, displaying almost equal activity to donepezil.

Equipotency to donepezil was confirmed by the administration of conjugates **13** and **15**, which displayed increases in SAP% of almost 35% and 34.8%, respectively. Furthermore, SAP% increased comparatively high in **4**, **12**, **14**, and **16**, ranging from 24% to 29.8%. A marginal rise in SAP% of 14.6% and 22% was noted by compounds **2** and **11**, respectively (Table 1 and Table 2).

#### 2.2.2. Effect on BACE1 and Amyloid-β

**Figure 4:**
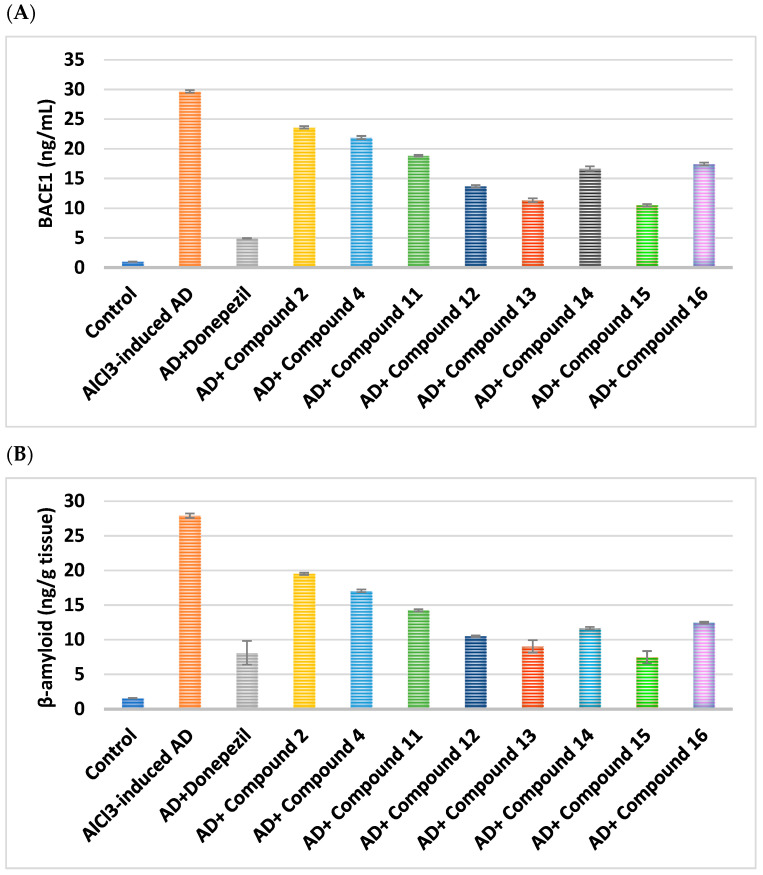
Effects on BACE1 and amyloid-*β* biomarker. Data is offered as means ± SE (n = 6). (**A**) BACE1 (ng/mL); (**B**) Amyloid-*β* (ng/g, Tissue).

*β*-Amyloid levels were dramatically reduced by 73.2% after intervention with compound **15**, demonstrating its superiority over donepezil (which reduced these levels by 70.9%). *β*-amyloid levels were significantly decreased by 62.3% and 67.6%, respectively, following administration of hybrids **12** and **13**, fostering equipotency to donepezil. Comparing compounds **11**, **16**, and **14** to donepezil, the former illustrated a significant reduction in *β*-amyloid by 48.9%, 55.4%, and 58.4%, respectively, whereas compounds **2** and **4** displayed a moderate reduction in *β*-amyloid by 30.2% and 38.9%, respectively (Figure 4).

Simultaneously, hybrids **12**, **13**, and **15** disclosed a noteworthy drop in BACE1 levels of 53.9%, 61.7%, and 64.6%, respectively. BACE1 levels were found to be moderately reduced by 36.5%, 41.2%, and 43.8% after treatment with compounds **11**, **5**, and **14**, whereas BACE1 levels were minimally reduced by 20.4% and 26.2% after treatment with compounds **2** and **4**, respectively (Table 3 and Table 4).

#### 2.2.3. Impact on Brain Neurotransmitters

When compared to donepezil, compounds **12**, **13**, and **15** moderately raised DA levels by 3.2, 4.1, and 3.8-fold, while compounds **11**, **14**, and **16** produced an extremely high elevation of 4.6, 4.3, and 4.4-fold.

**Figure 5:**
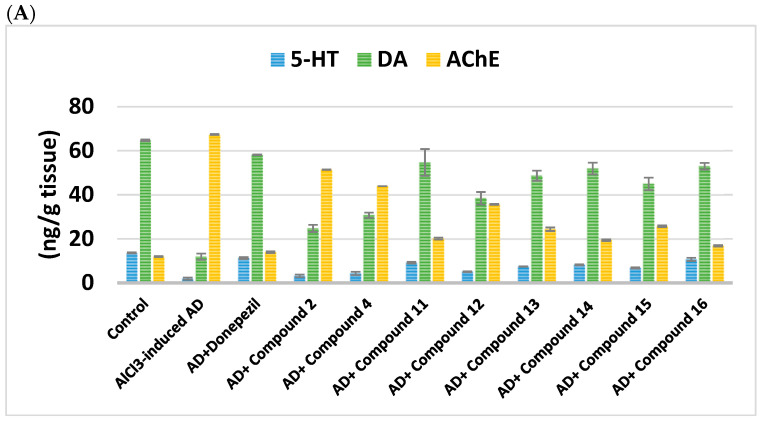

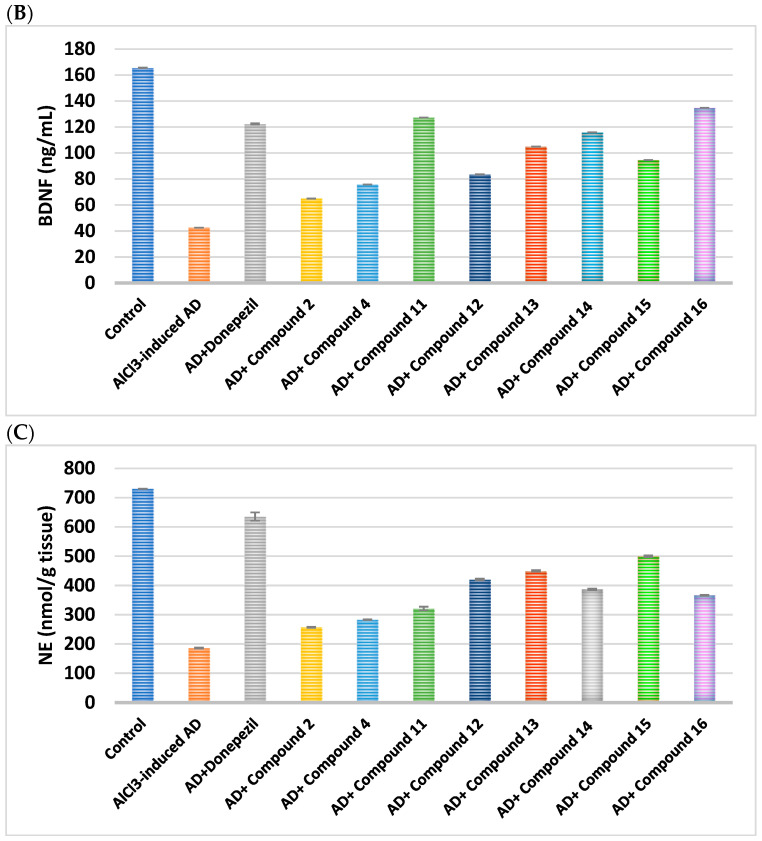
Impact on brain neurotransmitters: (**A**) 5-HT, DA, and AChE; (**B**) BDNF; (**C**) NE. Data is offered as means ± SE (n = 6).

Additionally, compounds **11**, **14**, and **16** demonstrated a moderate rise in the level of NE by 1.7, 2.1, and 2 times, respectively, in contrast to donepezil; nevertheless, compounds **12**, **13**, and **15** effectively enhanced the amount of NE by 2.2, 2.4, and 2.7, respectively (Figure 5).

Conjugates **12**, **13**, and **15** elevated 5-HT levels moderately by 2.8-, 4.1-, and 3.7-fold, respectively, whereas hybrids **11**, **14**, and **16** markedly enhanced 5-HT levels by 5.0-, 4.5-, and 5.9-fold, respectively. Additionally, as compared to donepezil, hybrids **2** and **4** showed a slight improvement in 5-HT levels of 1.7- and 2.4-fold, respectively.

While compounds **13** and **14** comparatively raised BDNF levels by 2.4- and 2.7-fold, respectively, compounds **11** and **16** demonstrated greater elevation of BDNF levels compared to donepezil by 3- and 3.1-fold, respectively. When compared to donepezil, hybrids **12** and **15** demonstrated an acceptable increase of 2- and 2.2-fold, respectively. More importantly, a significant reduction in AChE was seen by 70.1%, 71.2%, and 75%, respectively, following the administration of compounds **11**, **14**, and **16**. AChE was considerably reduced by compounds **12**, **13**, and **15** by 47%, 63.8%, and 61.8%, respectively. Compounds **2** and **4** showed a moderate reduction in AChE levels by 23.6% and 34.8%, respectively, in contrast to donepezil (AChE reduction of 79.3%) (Table 5 and Table 6).

#### 2.2.4. Impact on Neuroinflammatory Biomarkers

**Figure 6:**
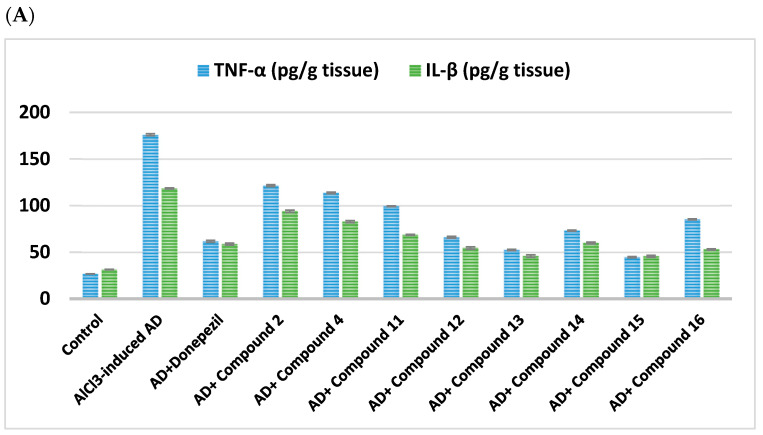

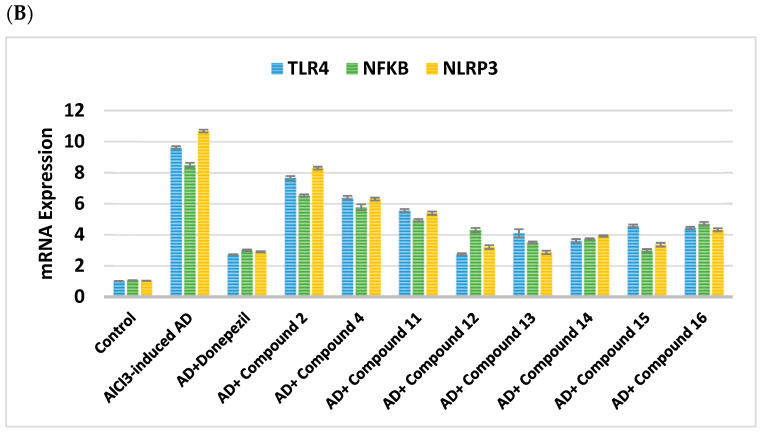
Impact on (**A**) TNF-*α* and IL-1*β*; (**B**) mRNA expression of TLR4, NFκβ, and NLRP3. Data is recorded as means ± SE (n = 6).

Examining the impact of the novel hybrids on IL-1*β* and TNF-*α*, along with mRNA expression levels of NF*κβ*, TLR4, and NLRP3, highlighted that administration of compounds **12**, **13**, **15**, and **16** drastically lowered IL-1*β* levels by 53.9%, 61.3%, 61.1%, and 55.2%, respectively. This exceeds the pronounced 50.3% reduction in donepezil inhibition. Hybrids **11** and **14** showed a moderate reduction in IL-1*β*, by 42.1% and 49.3%, respectively, while compounds **2** and **4** only showed acceptable inhibitory activity, by 20.6% and 30%, respectively (Figure 6).

Concurrently, compounds **13** and **15** dramatically decreased TNF-*α* levels, outperforming the donepezil inhibitory effect by 70.2% and 74.6%, respectively. Hybrids **11**, **12**, **16**, and **14** displayed a significant decline in TNF-*α* levels of 43.6%, 62.5%, 51.6%, and 58.4%, respectively, in contrast to hybrids **2** and **4**, which displayed an average decline of 31% and 35.4% (Table 7 and Table 8).

mRNA expression levels of NF*κβ* upon treatment with hybrid **15** displayed paramount reduction by 64.8%, corresponding to the effect of donepezil. Excellent NF*κβ* reduction was achieved by hybrids **13** and **14** by 58.8% and 56%, respectively. Hybrids **11**, **12**, and **16** demonstrated notable NF*κβ* level reduction by 41.8%, 49.2%, and 44.5%, respectively.

mRNA expression levels of TLR4 upon administration of hybrid **12** displayed superior reduction by 71.4%, analogous to the impact of donepezil. Outstanding TLR4 reduction was achieved by hybrids **13** and **14** by 57.5% and 62.6%, respectively. Hybrids **11**, **15**, and **16** confirmed remarkable TLR4 level reduction by 42.2%, 52.5%, and 53.9%, respectively.

The expression levels of NLRP3 mRNA upon treatment with hybrid **13** displayed major reduction by 73.2%, equivalent to the impact of donepezil. Excellent NLRP3 reduction was achieved by hybrids **12**, **14**, and **15** by 69.9%, 63.2%, and 68.5%, respectively. Hybrids **11** and **16** established significant NLRP3 level reduction by 49.5% and 59.5%, respectively.

#### 2.2.5. Effect on Caspase-1, BAX, and Bcl-2

Hybrid **15** demonstrated superior downregulation of Caspase-1 levels by 69.1%, comparable to the pronounced effect of donepezil on Caspase-1 levels. Compounds **12** and **13**, significantly suppressed Caspase-1 gene expression by 52.3% and 59.5%, respectively. Hybrids **11**, **14**, and **16** established moderate Caspase-1 level reductions of 37.8%, 46.6%, and 47%, respectively.

The BAX gene expression was dramatically suppressed by conjugates **12**, **13**, **14**, and **15** by 40% to 53.8%. With corresponding values of 29.3% and 36.5%, compounds **11** and **16** showed a rather small BAX inhibitory activity. In contrast to donepezil, compounds **2** and **4** showed a slight downregulatory effect of 16% and 23.3%, respectively.

**Figure 7:**
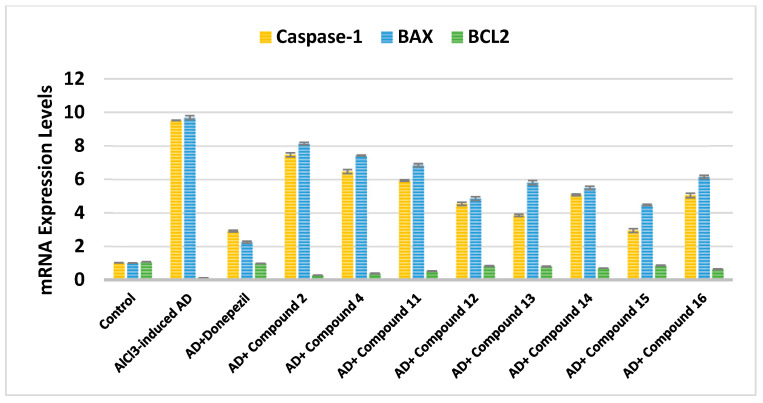
Effects on apoptosis of biomarkers Caspase-1, BAX, and Bcl2. The data are offered as means ± SE (n = 6).

In contrast, hybrids **11** and **16** demonstrated substantial upregulation by 4.3 and 5.2 folds, whereas compounds **12**, **13**, **14**, and **15** significantly elevated Bcl-2 gene expression by 6.8-, 6.5-, 5.8-, and 7.1-fold, respectively (Figure 7). Every synthesized drug exhibited a BAX/Bcl-2 ratio between 5.2 and 30.1, suggesting that they might increase brain cell proliferation by shifting the ratio to lower values (Table 9 and Table 10) [79].

#### 2.2.6. Effect on WNT Signaling Pathway

**Figure 8:**
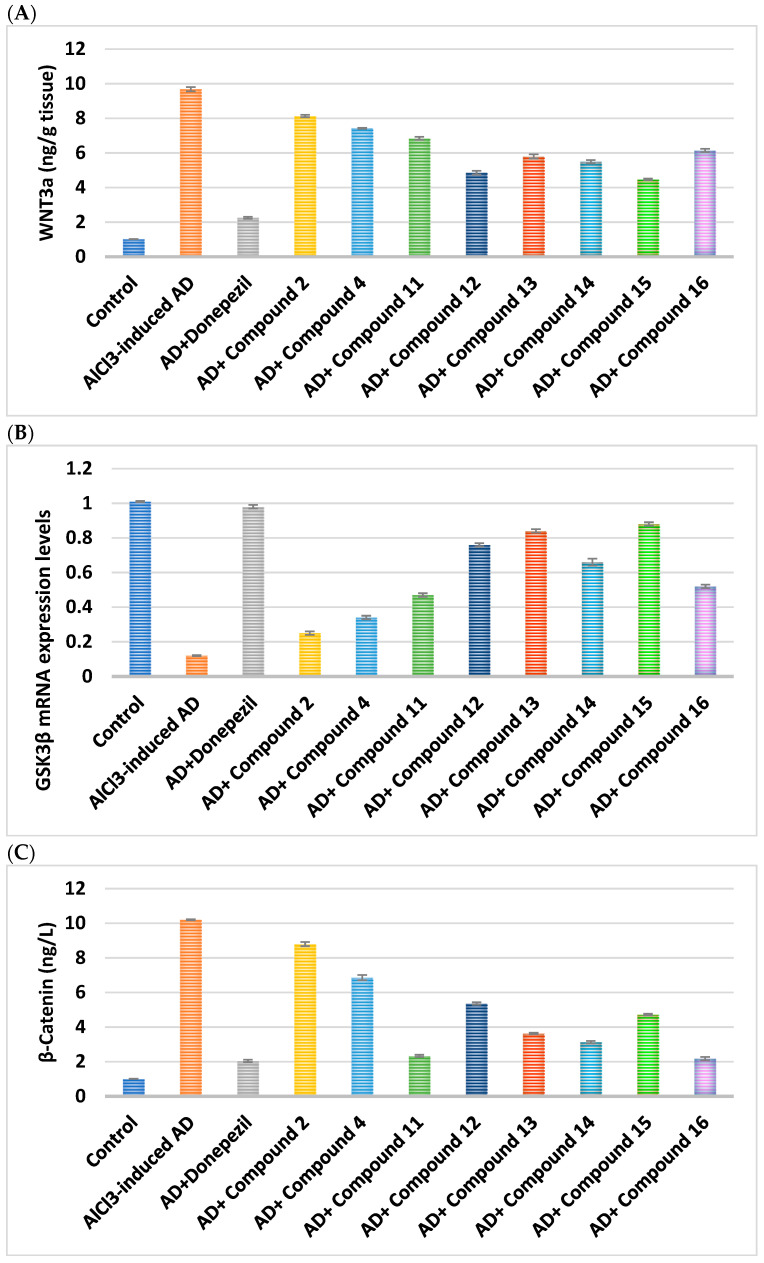
Effects on Wnt3/*β*-catenin/GSK3*β* signaling pathway. Data is recorded as means ± SE (n = 6). (**A**) Wnt3; (**B**) GSK3*β* signaling; (**C**) *β*-catenin.

Examining the impact on the WNT signaling pathway (Figure 8), compounds **12**, **13**, and **15** showed strong elevation of Wnt3a levels by 6.3-, 7-, and 7.3-fold, respectively, whereas compounds **11**, **16**, and **14** demonstrated moderate elevation by 3.9-, 5.5-, and 4-fold, respectively. Wnt3a levels were slightly elevated by 2 and 2.8 fold in conjugates **2** and **4**, respectively.

Furthermore, compared to donepezil, compound **15** dramatically enhanced the amount of *β*-catenin 8.8 times. The levels of *β*-catenin were elevated 7.7 times in compound **13**. Meanwhile, compounds **2** and **4** displayed a slight rise in *β*-catenin levels by 2.7- and 3.9-fold, respectively, while compounds **11**, **12**, **16**, and **14** demonstrated a moderate elevation in *β*-catenin levels by 4.8-, 6.3-, 4.6-, and 5.3-fold, respectively.

Conversely, compounds **11**, **13**, **16**, and **14** significantly reduced GSK3*β* gene expression by 64.4% to 78.6%. The GSK3*β* gene expression was moderately downregulated by hybrids **12** and **15**, by 47.4% and 53.7%, respectively, whereas compounds **2** and **4** showed the smallest degree of downregulation, by 13.7% and 32.7%, respectively (Table 11 and Table 12).

#### 2.2.7. Impacts on Brain Oxidative Stress

**Figure 9:**
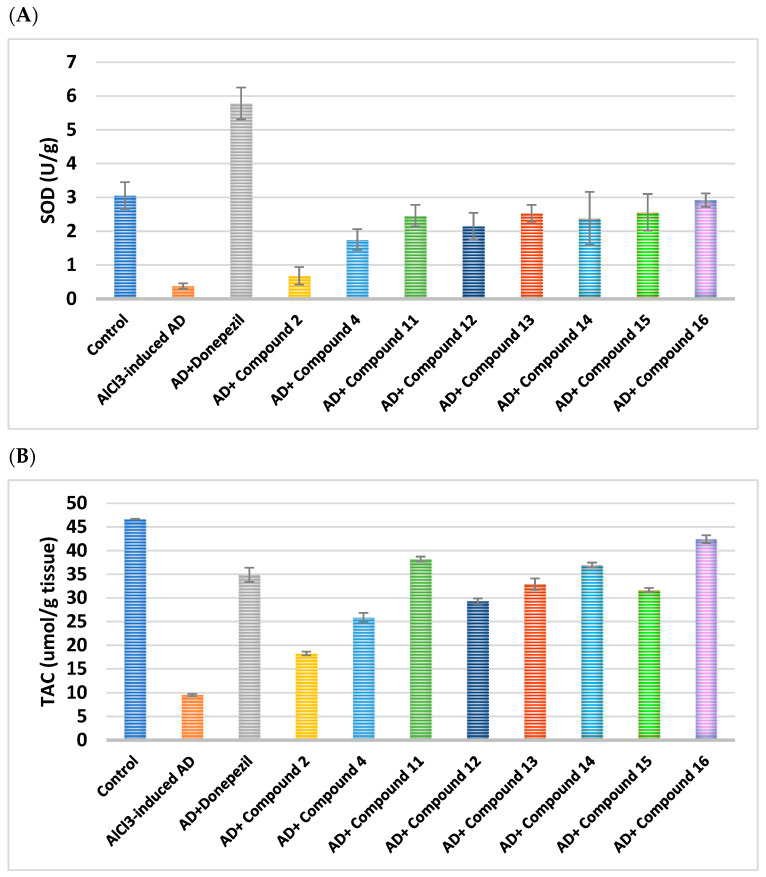

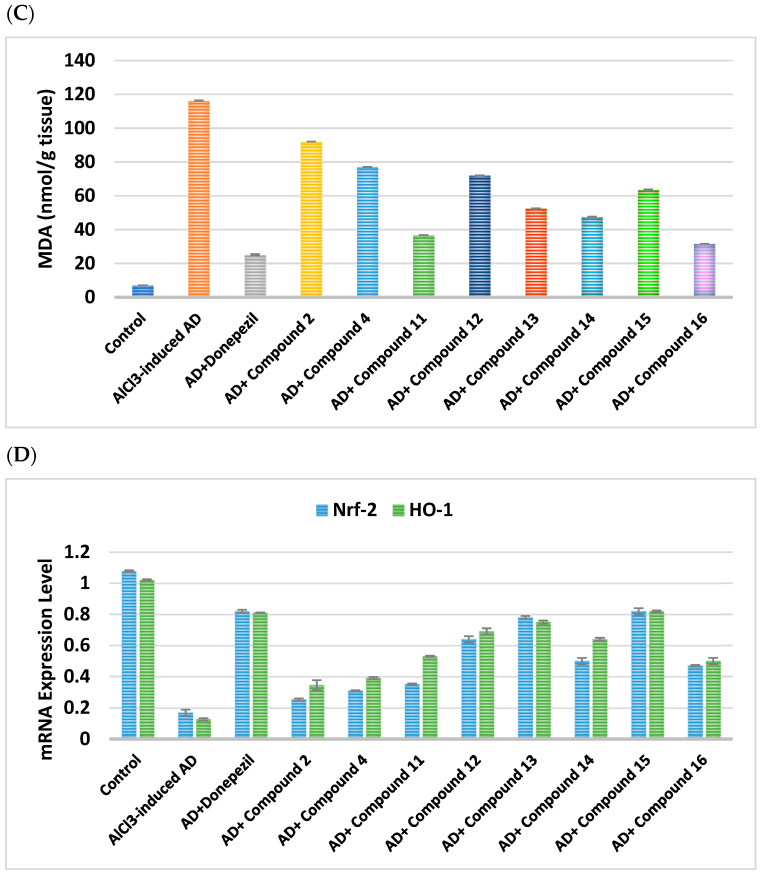
Effect on: (**A**) SOD, (**B**) TAC, (**C**) MDA, and (**D**) Nrf-2 and HO-1 expression. Data is recorded as means ± SE (n = 6).

TAC levels improved by 4, 3.8, and 4.4 times, respectively, upon administering conjugates **11**, **14**, and **16**, outperforming donepezil (Figure 9). In a manner comparable to donepezil, compounds **12**, **13**, and **15** showed improvements in TAC of approximately 3, 3.4, and 3.3 times, whereas compounds **2** and **4** showed only slight enhancements in TAC of 1.9 and 2.7 times, respectively.

Additionally, compounds **11**–**16** exhibited a significant improvement in SOD activity levels, ranging from 2.15 to 2.92 U/g tissue, whereas compound **4** showed a little rise in SOD activity levels, reaching 1.75 U/g tissue.

Furthermore, compounds **11**, **13**, **14**, and **16** demonstrated an exceptional reduction in the MDA brain content, with respective values of 68.4%, 54.7%, 59%, and 72.8%. Following treatment with compounds **4**, **12**, and **15**, there was a moderate decrease in MDA levels of 33.6%, 38%, and 45.2%, respectively (Table 13 and Table 14).

mRNA expression levels of HO-1 upon administration of hybrid **15** displayed superior elevation, by 6.7-fold, analogous to the impact of donepezil administration. Outstanding HO-1 elevation was achieved by hybrid **13**, by 6.2-fold. Hybrids **12** and **14** confirmed remarkable HO-1 level elevation by 5.7- and 5.3-fold, respectively. Relatively good elevation of HO-1 level was demonstrated by **11** and **16**, by 4.4- and 4.1-fold, respectively.

The expression levels of Nrf-2 mRNA upon treatment with hybrids **13** and **15** displayed major elevation by 4.8- and 5.1-fold, respectively, equivalent to the impact of donepezil. Moderate Nrf-2 elevation was achieved by hybrids **12**, **14**, and **16** by 4-, 3.1-, and 2.9-fold, respectively.

#### 2.2.8. Histopathological Examination

**Figure 10:**
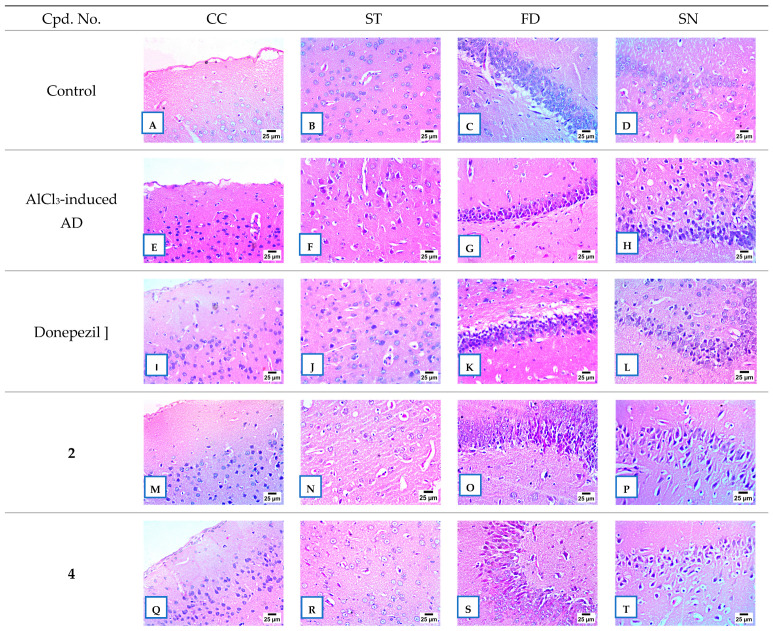
A micrograph of a section in the CC, ST, FD, and SN. (**A**–**D**) Negative control. (**E**–**H**) AlCl_3_-induced AD group, showing a high number of shrunken degenerated neurons with severe NP in the CC, NP, FD, and necrotic neurons in ST. (**I**–**L**) Donepezil group, showing moderate NP in CC, ST, FD, and SN. (**M**–**P**) Compound 2 group, showing moderate NP in CC, FD, and SN, in addition to normal histology in ST. (**Q**–**T**) Compound 4 group, showing moderate NP in CC and SN, a moderate number of necrotic neurons in FD, as well as normal histology of ST neurons.

**Figure 11:**
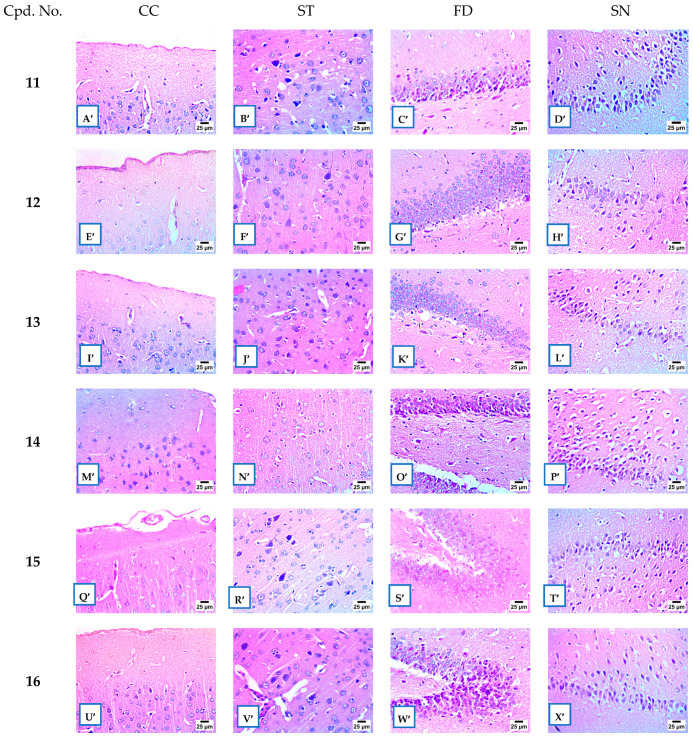
A micrograph from section in CC, ST, FD, and SN. (**A**′–**D**′) Compound **11,** showing mild NP in neurons in CC and moderate NP in ST, FD, and SN. (**E**′–**H**′) Compound **12,** showing normal histology in CC and FD, as well as mild NP in ST and SN. (**I**′–**L**′) Compound **13,** showing normal histology of CC and FD, and moderate NP in ST and SN. (**M**′–**P**′) Compound **14,** showing moderate NP in CC, FD, and SN, normal histology of neurons in ST. (**Q**′–**T**′) Compound **15,** showing normal histology of CC and FD, and moderate NP in ST and SN. (**U**′–**X**′) Compound **16,** showing mild NP in neurons in CC, as well as moderate NP in neurons of ST, FD, and SN.

The brain tissues of rats given donepezil [70] experienced significant nuclear pyknosis (NO) in the cerebral cortex (CC), striatum (ST), fascia dentata (FD), and subiculum neurons (SN) (Figure 10I–L). Rats administered **2** and **4** additionally demonstrated significant NP in the CC, FD, and SN, whereas the striatal neurons’ histological structures remained normal (Figure 10M–T)). There was histologically minor NP in the CC and significant NP in FD, ST, and SN in hybrids **11** (Figure 11A′–D′) and **16** (Figure 11U′–X′)).

The CC neurons (Figure 11E′,I′,M′) and FD (Figure 11G′,K′,O′)) displayed normal histological structures following administration of hybrids **12**, **13**, and **15**, respectively. Compounds **13** (Figure 11J′,N′) and **15** (Figure 11R′,T′) elicited moderate pyknosis in striatal and subicular neurons. In the ST and SN of hybrid **12**, minimal pyknosis was seen (Figure 11F′,H′). The histological anatomy of neurons in the CC, FD, and SN evidenced significant pyknosis after treatment with hybrid **14** (Figure 11M′,O′,P′).

#### 2.2.9. AChE and BChE Inhibition

**Figure 12:**
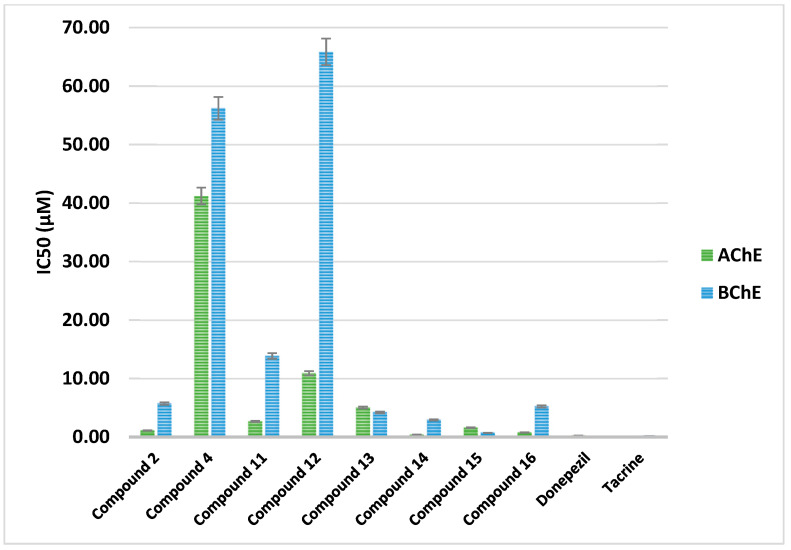
IC_50_ values of hybrids investigated against AChE/BChE.

The potential inhibitory effect of all produced hybrids on AChE and BChE were assessed and compared to donepezil and tacrine, (Figure 12). Significant advancements have been achieved in the development of potent and specific BChE inhibitors able to raise brain ACh levels with few adverse peripheral effects. Based on their respective IC_50_ values of 1.10, 0.39, and 0.76 µM, compounds **2**, **14**, and **16** were found to be the most effective AChE inhibitors. Inhibitory activity against AChE was shown to be promising for compounds **11**, **13**, and **15**, with corresponding IC_50_ values of 2.67, 5.04, and 1.59 µM. Comparable IC_50_ results of 10.90 µM indicated that compound **12** similarly exhibited considerable AChE inhibitory effect. On par with tacrine, compound **15** also demonstrated remarkable BChE inhibitory action, with an IC_50_ value of 0.70 µM. Additionally, with IC_50_ values ranging from 2.93 to 5.72 µM, compounds **2**, **13**, **14**, and **16** show remarkable BChE inhibitory action. The IC_50_value of 13.85µM for compound **11** indicated substantial BChE inhibition.

The ratio of the IC_50_ values for BChE and AChE is used to compute the Selectivity Index (SI). When the result is less than 1, preferential inhibition of BChE is observed. Only the produced hybrids **13** and **15**, with values of 0.84 and 0.44, respectively, showed greater selectivity towards BChE, despite the fact that the majority of the hits presented substantial inhibition of AChE, emphasizing the ability to increase ACh levels in the brain while drastically reducing adverse effects on the peripheral nervous system (Table 15 and Table 16).

### 2.3. SAR Analysis

**Figure 13:**
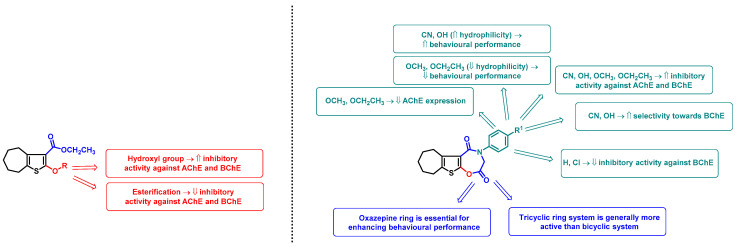
SAR of cyclohepta[*b*]thieno[1,4]oxazepine hybrids.

This investigation facilitated understanding of the structure–activity relationship through the biological effects of the novel derivatives (Figure 13). The significance of introducing the oxazepine ring can be witnessed by the remarkable learning, memory, and SAP% scores of compounds **11**–**16** on behavioral tests, which suggest that the tricyclic ring system is generally better than the bicyclic ring system. It was verified by looking at the substitution on the *N*-phenyl ring that compounds **13** and **15**, which have hydrophilic groups on the *para* position, performed the best in behavioral testing. This is further corroborated by tricyclic compounds **14** and **16**, which possessed less hydrophilic *para*-alkoxy modification on the *N*-phenyl ring and performed well in behavioral testing, however slightly worse than compounds **2** and **4**. Bicyclic hybrids are essentially less active than tricyclic compounds, as evidenced by the satisfactory, yet modest, performance displayed by compounds **2** and **4** in behavioral testing.

Similarly to behavioral performance, the tricyclic ring system actually takes precedence over the bicyclic ring system, as demonstrated by the influence of compounds **11**–**16** on the suppression of AChE expression levels. Hybrids **14** and **16** displayed the greatest suppression of AChE expression levels, demonstrating the significance of the para-alkoxy group on the N-phenyl ring.

Further supporting the crucial need for a tricyclic ring system is the fact that tricyclic compounds (compounds **11**–**16**) outperform bicyclic compounds (compounds **2** and **4**) in reducing the levels of inflammatory biomarkers. While switching to alkoxy groups typically reduced the inhibition of inflammatory indicators, tricyclic compounds **13** and **15**, which contained *para*-hydroxy and *para*-cyano groups, showed the strongest anti-inflammatory action.

The inhibition effect of hybrids **14** and **16** against AChE exhibits a SAR, underscoring the significance of the alkoxy group in tricyclic hybrids. Regardless of the number of fused rings, the inhibitory activity against AChE is typically improved by the presence of an OH group.

The significance of having *para*-hydroxy or alkoxy groups, which result in maximal BChE inhibitory activity, was established by SAR contingent on the inhibitory activities of **2**, **14**, **15**, and **16** against BChE. It was shown that BChE inhibition is decreased by unsubstituted or *para*-chloro-substituted phenyl rings.

### 2.4. In Silico ADME Evaluation

In silico drug-likeness, ADME, and physicochemical assessments were conducted on all hybrids in addition to the common reference drugs donepezil and tacrine. Various physicochemical characteristics, such as lipophilicity and rotating contacts, are noted in Table 17. Five of the new compounds featured four H-bond donor groups, exactly as observed with donepezil; nevertheless, all of them shared three to four H-bond acceptor groups. In contrast, hybrids **2** and **15** featured one H-bond donor group, which is analogous to tacrine.

The capacity of drugs to infiltrate the BBB is a crucial pharmacokinetic characteristic of AD treatments. For medications to easily pass across the blood–brain barrier, PSA ≤ 70 Å^2^ and log P = 2–5 are required [80,81]. MlogP values appeared over 3.0 for compounds **11**, **12**, and **14**, and above 2.0 across all synthesized compounds. Large MlogP values ensure that the synthetic compounds can cross the BBB. Table 18 displays the values of the TPSA. SwissADME’s PAINS discovered that none of the compounds prompted any alerts [82]. All hybrids thrived better than donepezil and tacrine, with synthetic accessibility scores extending from 3.14 to 3.58, demonstrating easy wide scale synthesis (Table 17).

Each synthesized molecule is assigned a TPSA of less than 200 Å^2^. The range of the percentage ABS among all hits was between 81.11 to 89.06 percent, corresponding to donepezil and tacrine. This implies that these derivatives could possess the required bioavailability and permeability of cell membranes. Percent ABS, or absorption, was also computed using the formula % ABS = 109 − (0.345 × TPSA) (Table 18).

Table 19 lists the anticipated ADME and pharmacokinetic attributes of the hybrids under investigation. In line with the findings, all the substances that were evaluated indicated high gastrointestinal (GI) absorption and did not inhibit p-glycoprotein (P-gp).

The unit used to indicate the BBB penetration rate is cm/s. In contrast to BBB-molecules (Category 0), which exhibited log BBB ≤ −1, BBB+ molecules (Category 1) exhibited log BBB > −1. The output value, which ranges from 0 to 1, shows the probability that an input is BBB+. In comparison to donepezil, hybrids **11** and **12** were predicted to have greater BBB permeability, whereas hybrids **2** and **4** had a moderate probability of BBB permeability. Additionally, the entire examined Cytochrome P450 (CYP) isomers are inactivated by the newly generated oxazepine derivatives. Each of the compounds examined, with the exception of **15**, **16**, and **14**, do not inhibit CYP2D6. Furthermore, every anticipated analog displayed CYP3A4 inhibition, with the exception of compounds **2**, **4**, and **12**. The skin permeability coefficient values (log K_p_), where K_p_ is expressed in cm/s, for the tested compounds were minimal. (Table 19).

The assorted drug-likeness rules, i.e., Lipinski [83], Veber [84], Ghose [85], Muegge [86], and Egan [87], were compelled to evaluate the molecule to be an influential drug candidate. The number of violations of the previously mentioned regulations and their bioavailability scores are displayed in Table 20. The Lipinski (Pfizer) strain is subject to the present setting rule-of-five (RO5), which asserts that every molecule under examination is drug-like. All compounds uphold the norm and have the potential to be medications, according to the predictions of the Ghose, Veber, Muegge, and Egan rules. For each of the compounds under examination, the bioavailability score was 0.55 (Table 20).

**Figure 14:**
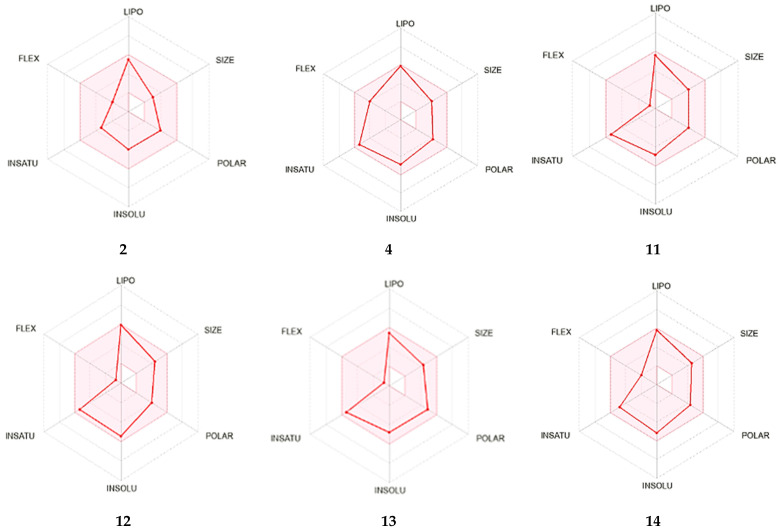

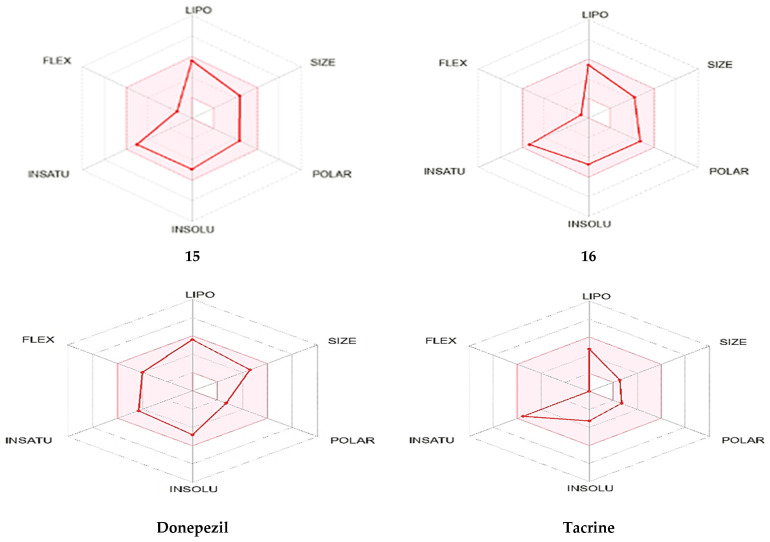
Bioavailability radar comparing the studied drugs with donepezil and tacrine.

The following traits are most correctly represented by the pink surface of the bioavailability radar: flexibility, no more than six rotatable bonds; solubility; sp3 hybridization fraction, not less than 0.23; log S, not greater than 7; lipophilicity; XLOGP3 range between 4.79 and + 6.81; MW, 330–430 g/mol MW. Because the molecules are confined inside the pink surface, they are potentially bioavailable when taken orally (Figure 14).

### 2.5. In Silico Toxicity Evaluation

Based on online resources ProTox-II (https://tox-new.charite.de/protox_II, accessed on 1 February 2025) and pkCSM (http://biosig.unimelb.edu.au/pkcsm/prediction, accessed on 1 February 2025), Table 21 articulates the estimated toxicity of the most potent derivatives, **2**, **14**, and **16,** alongside the two reference anti-AD medications, tacrine and donepezil [88,89]. The pkCSM online tools indicate that the only compounds that are safer than tacrine are compounds **2** and **14**, which are comparable to donepezil in that they fail to demonstrate any signs of AMES toxicity. Additionally, the highest tolerated human dose value (0.668 log mg/kg/day) for active hit **2** was greater than that of tacrine and donepezil. Compared to donepezil and tacrine, **14** and **16** have higher oral rat acute toxicity (LD_50_) values. Additionally, all hits exhibited greater Oral Rat Chronic Toxicity (LOAEL) values than donepezil and tacrine. The proper repolarization of the pulse is controlled by the potassium channels expressed by the human Ether-à-go-go Related Gene (hERG). Serious cardiovascular disorders might arise from any blockage or dysfunction of these channels in heart cells. As a result, the pharmaceutical sector is highly concerned about drug-induced potassium channel blocking. It was anticipated that no active hybrids would inhibit hERG-I or hERG-II. Hepatotoxicity was examined using the pkCSM server, and it was found that none of the active hits were hepatotoxic. The only hybrid that displayed skin sensitivity was hybrid **2**.

Tacrine was expected to fall into class two (GHS), while donepezil and all hybrids were predicted to fall into class four using the ProTox-II detection web tool (Table 21). All hits were found to display higher LD_50_ values than donepezil and tacrine. All active derivatives have been determined to be non-mutagenic and non-immunotoxic. Furthermore, conjugates **2** and **16**, unlike donepezil, were expected to be non-cytotoxic.

### 2.6. Molecular Docking

**Figure 15:**
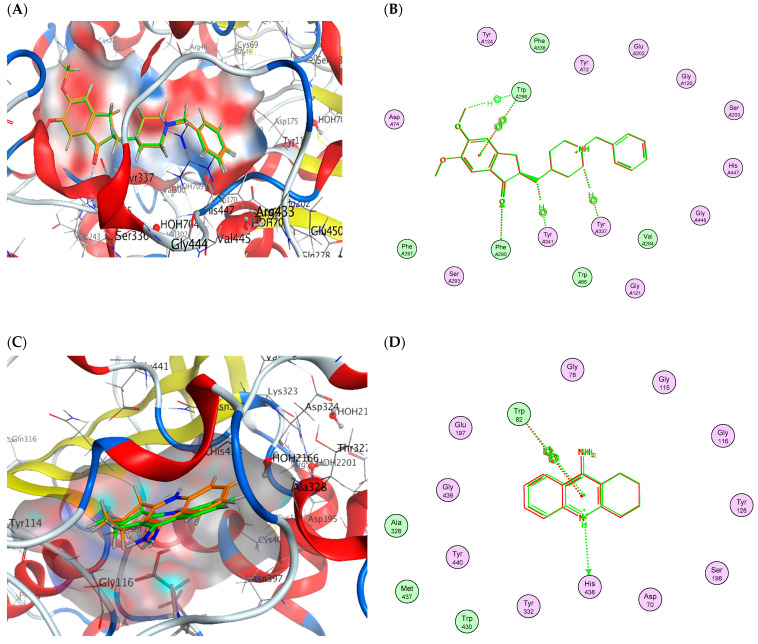
(**A**) Bound ligand (green) and re-docked pose (orange) of donepezil (RMSD = 0.117 Å). (**B**) 2D complex overlay in 4EY7. (**C**) Bound ligand (green) and re-docked pose (orange) overlay (3D) of tacrine (RMSD = 0.589 Å). (**D**) Two-dimensional complex overlay in 4BDS.

The reliability of docking was validated by self-docking the reference antagonists, tacrine and donepezil, into the active sites of AChE (PDB ID: 4EY7) and BChE (PDB ID: 4BDS), respectively. The validation was successful, with energy scores (S) of −17.710 kcal/mol and −10.080 kcal/mol, in addition to marginal RMSD values between the native ligands and the re-docked pose of 0.117 Å and 0.589 Å, respectively. Figure 15 shows that the re-docked location could superimpose the native ligand while also occupying crucial contacts produced by the bound ligand (Table 22) with the active sites of both AChE and BChE, supporting the reliability of the validation.

Phe295 was involved in one H-bond interaction between donepezil and the AChE pocket (PDB: 4EY7). The residues Trp286, Tyr341, and Tyr 337 in AChE were also observed to engage in three H–arene interactions with donepezil. Additionally, a single arene–arene interaction through Trp286 was revealed. Tacrine was shown to engage with the BChE pocket by forming an H-bond with a His438 residue (PDB: 4BDS). It was observed that tacrine and His438 produced one H–arene interaction and that the Trp82 residue produced one arene–arene contact (Table 22).

**Figure 16:**
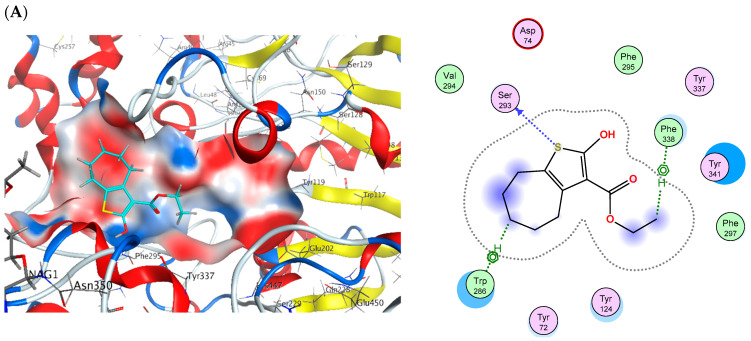

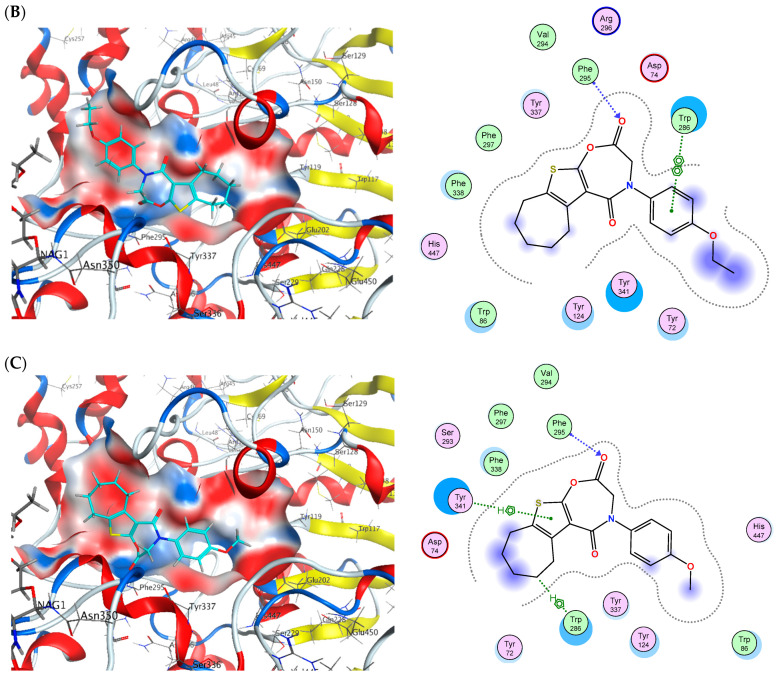
Two-dimensional and three-dimensional binding modes of the most promising hybrids—(**A**) **2**, (**B**) **14**, and (**C**) **16**—with AChE (PDB ID: 4EY7).

**Figure 17:**
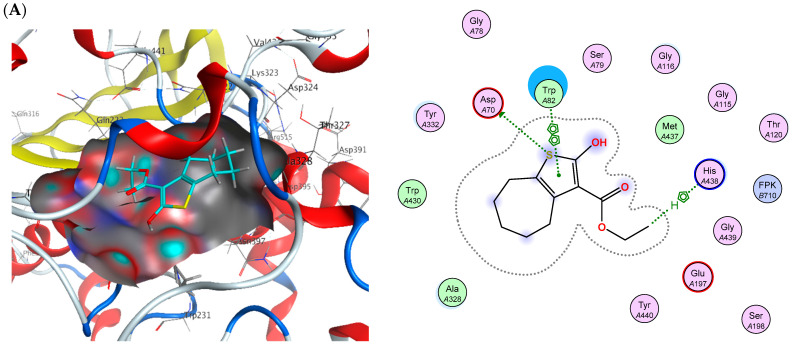

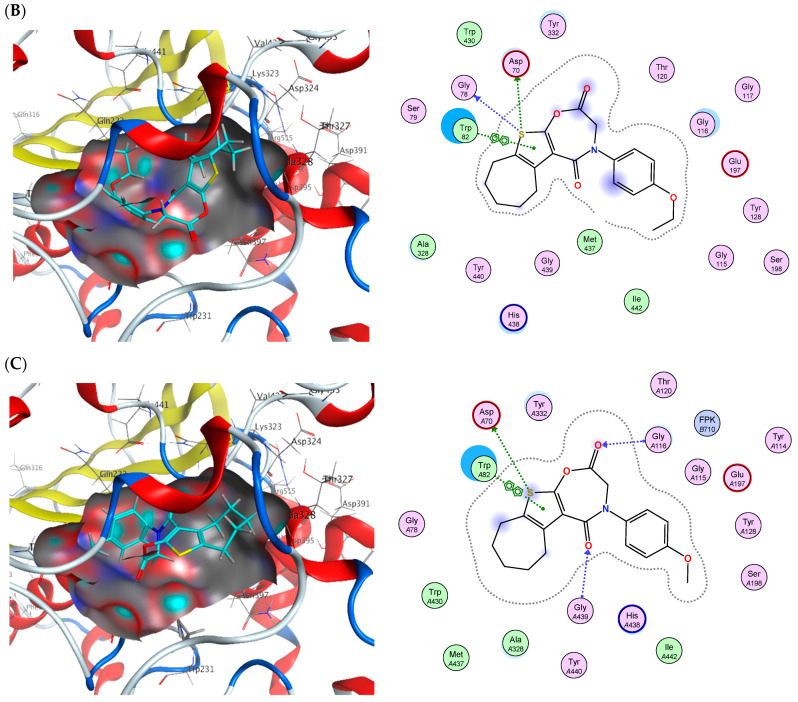
Two-dimensional and three-dimensional binding modes of the most promising hybrids—(**A**) **2**, (**B**) **14**, and (**C**) **16**—with BChE (PDB ID: 4BDS).

The binding energies along with interactions of the most potent derivatives—**2**, **14**, and **16**—with both AChE and BChE were inspected. With respective values of −14.45, −15.09, and −14.74 kCal/mol, the binding energies were found to be rather proximal to donepezil’s binding energy with AChE. In a comparable manner, it was discovered that the binding energies with BChE were very similar to those of tacrine, reporting −14.97, −16.41, and −18.10 kCal/mol, respectively. As they interact with the AChE pocket, the three most active hybrids were discovered to interact with the same amino acid residues—Phe295, Tyr341, and Trp286—that are implicated in donepezil interaction to the binding site (Figure 16). Both an additional H-bond with Ser293 and an additional H–arene contact with the Phe338 residue of AChE were observed by compound **2**. Additionally, the three most active hits displayed binding interactions with BChE (Figure 17), including two amino acid residues that are also implicated in tacrine binding: His438 and Trp82. Asp70 additionally produced an additional H-bond with the three hits. Upon compound **14** binding, a second H-bond with Gly78 was discovered. Two further H-bonds with Gly439 and Gly116 were discovered upon binding of compound **16** (Table 23).

## 3. Materials and Methods

### 3.1. Chemistry

#### 3.1.1. General Details

See Supplementary Information.

#### 3.1.2. Synthesis

**Ethyl 2-amino-5,6,7,8-tetrahydro-4*H*-cyclohepta[*b*]thiophene-3-carboxylate (1)**

Created utilizing the documented process [73].

**Ethyl 2-hydroxy-5,6,7,8-tetrahydro-4*H*-cyclohepta[*b*]thiophene-3-carboxylate (2)**

Compound **1** (0.005 mol) was dissolved in 30 milliliters of water and 4.5 milliliters of 37% *w*/*v* hydrochloric acid. The diazonium salt was obtained by adding a dropwise, stirred cold solution of sodium nitrite (0.005 mol) in water (1 mL) to the cooled amine solution (0–5 °C). To guarantee a full response, stirring was maintained for fifteen minutes. Adding an equivalent amount of water and boiling it in a warm water bath at 50 °C for 30 min made hydrolysis simple. Once N_2_ evolution stopped, the precipitate that had formed was filtered, and crystallized from pure ethanol to produce (**2**) [73].

**Ethyl 2-(2-chloroacetoxy)-5,6,7,8-tetrahydro-4*H*-cyclohepta[*b*]thiophene-3-carboxylate, (4)**

A solution of compound **2** (0.01 mol) in *N*,*N*-dimethyl formamide (DMF) (20 mL) with five drops of triethylamine was refluxed for six hours before the addition of chloroacetyl chloride (0.01 mol). The mixture was introduced into ice-cold water once it cooled. Following a water rinse and a dry filter, the solid crystallized from the ethanol.

Yield: 92%; m.p.: 155–156 °C; IR (KBr, cm^−1^) = 3037 (sp^2^ C-H), 2930, 2840 (sp^3^ C-H), 1707, 1690 (2 C=O ester); ^1^H NMR (400 MHz, CDCl_3_): *δ* = 4.35 (q, *J* = 6.3 Hz, 2H, -CH_2_-CH_3_), 4.25 (s, 2H, CO-CH_2_-Cl), 2.96 (t, *J* = 8.2 Hz, 2H, cycloheptane-Hs), 2.68 (t, *J* = 7.3 Hz, 2H, cycloheptane-Hs), 1.73–1.51 (m, 6H, cycloheptane-Hs), 1.38 (t, *J* = 6.2 Hz, 3H, CH_2_-CH_3_); ^13^C NMR (100 MHz, CDCl_3_): *δ* = 165.97 (CO-CH_2_-Cl), 162.90 (CO-OCH_2_CH_3_), 160.04, 136.30, 125.65, 111.96, 59.81, 39.36, 31.59, 28.78, 28.40, 27.43, 25.93, 13.19.; MS (EI, 70 eV): *m*/*z* (%) = 318 [M**^. +^**+2] (9.14%), 316 [M**^. +^**] (10.11%), 42 (100%); Anal. Calc. for C_14_H_17_ClO_4_S (316.80): C, 53.08; H, 5.41; S, 10.12. Found: C, 53.10; H, 5.43; S, 10.13.

**4-(Aryl)-3,4,7,8,9,10-hexahydro-6*H*-cyclohepta[4,5]thieno[3,2-*f*][1,4]oxazepine-2,5-dione (11–16)**

Typical operating procedure.

In ten milliliters of glacial acetic acid, the suitable amine (0.01 mol) and chloro-acetyl derivative 4 (0.01 mol) were refluxed for ten hours. After condensing the reaction mixture at reduced pressure, diethyl ether was employed to triturate it. To filter and crystallize the separate material, the proper solvent was utilized.

**4-Phenyl-3,4,7,8,9,10-hexahydro-6*H*-cyclohepta[4,5]thieno[3,2-*f*][1,4]oxazepine-2,5-dione, (11)**

Compound **11** was yielded upon the reaction of compound **4** and aniline.

Crystallized from ethanol. Yield: 89%; m.p.: 189–191 °C; IR (KBr, cm^−1^) = 3078 (sp^2^ CH), 2910, 2860 (sp^3^ C-H), 1670 (2 C=O); ^1^H NMR (400 MHz, CDCl_3_): *δ* = 7.37 (dd, *J* = 7.4, 1.4 Hz, 2H, Ar-H), 7.30–7.26 (m, 2H, Ar-H), 7.12–7.05 (m, 1H, Ar-H), 4.77 (s, 2H, oxazepine CH_2_), 2.85 (t, *J* = 8.2 Hz, 2H, cycloheptane-Hs), 2.72 (t, *J* = 7.3 Hz, 2H, cycloheptane-Hs), 1.72–1.52 (m, 6H, cycloheptane-Hs).; ^13^C NMR (100 MHz, CDCl_3_): *δ* = 164.07 (CO), 163.76 (CO), 155.82, 139.48, 135.89, 128.26, 125.64, 123.80, 121.61, 118.06, 47.06 (oxazepine CH_2_), 31.08, 28.39, 28.19, 27.43, 25.93.; MS (EI, 70 eV): *m*/*z* (%) = 327 [M**^. +^**] (40.74%), 54 (100%); Anal. Calc. for C_18_H_17_NO_3_S (327.40): C, 66.04; H, 5.23; N, 4.28; S, 9.79. Found: C, 66.01; H, 5.25; N, 4.29; S, 9.80.

**4-(4-Chlorophenyl)-3,4,7,8,9,10-hexahydro-6*H*-cyclohepta[4,5]thieno[3,2-*f*][1,4]oxazepine-2,5-dione (12)**

Compound **12** was yielded upon the reaction of compound **4** and 4-chloroaniline.

Crystallized from acetic acid as crystals. Yield: 85%; m.p.: 170–172 °C; IR (KBr, cm^−1^)= 3062 (sp^2^ CH), 2965, 2845 (sp^3^ C-H), 1703 (2C=O); ^1^H NMR (400 MHz, CDCl_3_): *δ* = 7.35 (d, *J* = 8.6 Hz, 2H, Ar-H), 7.28 (d, *J* = 8.7 Hz, 2H, Ar-H), 4.77 (s,2H, oxazepine CH_2_), 2.85 (t, *J* = 8.2 Hz, 2H, cycloheptane-Hs), 2.72 (t, *J* = 7.3 Hz, 2H, cycloheptane-Hs), 1.73–1.51 (m, 6H, cycloheptane-Hs).; ^13^C NMR (100 MHz, CDCl_3_): *δ* = 164.07 (C=O), 163.77 (C=O), 155.82, 138.25, 135.89, 129.02, 128.37, 125.64, 123.80, 118.06, 46.97 (oxazepine CH_2_), 31.08, 28.39, 28.19, 27.43, 25.93.; MS (EI,70 eV): *m*/*z* (%) = 363 [M**^. +^**+2] (7.60%), 361 [M**^. +^**] (17.67%), 328 (100%); Anal. Calc. for C_18_H_16_ClNO_3_S (361.84): C, 59.75; H, 4.46; N, 3.87; S, 8.86. Found: C, 59.73; H, 4.48; N, 3.88; S, 8.88.

**4-(2,5-Dioxo-2,3,7,8,9,10-hexahydro-6*H*-cyclohepta[4,5]thieno[3,2-*f*][1,4]oxazepin-4(5*H*)-yl)benzonitrile (13)**

Compound **13** was yielded upon the reaction of compound **4** and 4-aminobenzonitrile. Crystallized from ethanol. Yield: 89%; m.p.: 200–202 °C; IR (KBr, cm^−1^) = 3080 (sp^2^ CH), 2980, 2861 (sp^3^ CH), 2222 (CN), 1664 (2 C=O); ^1^H NMR (400 MHz, CDCl_3_): *δ* = 7.58 (d, *J* = 7.9 Hz, 2H, Ar-H), 7.50 (d, *J* = 7.7 Hz, 2H, Ar-H), 4.77 (s, 2H, oxazepine CH_2_), 2.85 (t, *J* = 8.2 Hz, 2H, cycloheptane-Hs), 2.72 (t, *J* = 7.3 Hz, 2H, cycloheptane-Hs), 1.73–1.51 (m, 6H, cycloheptane-Hs).;^13^C NMR (100 MHz, CDCl_3_): *δ* = 164.07 (C=O), 163.75 (C=O), 155.82, 140.82, 135.89, 132.45, 125.64, 118.76, 118.06 (CN), 117.28, 102.30 (C-CN), 47.05 (oxazepine CH_2_), 31.08, 28.39, 28.19, 27.43, 25.93.; MS (EI,70 eV): *m*/*z* (%) = 352 [M**^. +^**] (19.30%), 350 (100%); Anal. Calc. for C_19_H_16_N_2_O_3_S (352.41): C, 64.76; H, 4.58; N, 7.95; S, 9.10. Found: C, 64.75; H, 4.55; N, 7.96; S, 9.13.

**4-(4-Ethoxyphenyl)-3,4,7,8,9,10-hexahydro-6*H*-cyclohepta[4,5]thieno[3,2-*f*][1,4]oxazepine-2,5-dione (14)**

Compound **14** was yielded upon the reaction of compound **4** and 4-ethoxyaniline. Crystallized from ethanol. Yield: 85%; m.p.: 187–189 °C; IR (KBr, cm^−1^) = 3075 (sp^2^ CH), 2972, 2870 (sp^3^ CH), 1702, 1680 (2 C=O); ^1^H NMR (400 MHz, CDCl_3_): *δ* = 7.31 (d, *J* = 8.8 Hz, 2H, Ar-H), 6.85 (d, *J* = 8.8 Hz, 2H, Ar-H), 4.77 (s, 2H, oxazepine CH_2_), 4.04 (q, *J* = 6.7 Hz, 2H, -CH_2_-CH_3_), 2.85 (t, *J* = 8.2 Hz, 2H, cycloheptane-Hs), 2.72 (t, *J* = 7.3 Hz, 2H, cycloheptane-Hs), 1.73–1.51 (m, 6H, cycloheptane-Hs), 1.42 (t, *J* = 6.6 Hz,3H, -CH_2_-CH_3_).; ^13^C NMR (100 MHz, CDCl_3_): *δ* = 164.07 (C=O), 163.74 (C=O), 155.82, 149.71, 135.89, 134.95, 125.64, 122.81, 118.06, 114.58, 62.57 (-OCH_2_-CH_3_), 47.00 (oxazepine CH_2_), 31.08, 28.39, 28.19, 27.43, 25.93, 13.61; Anal. Calc. for C_20_H_21_NO_4_S (371.45): C, 64.67; H, 5.70; N, 3.77; S, 8.63. Found: C, 64.66; H, 5.72; N, 3.78; S, 8.60.

**4-(4-Hydroxyphenyl)-3,4,7,8,9,10-hexahydro-6*H*-cyclohepta[4,5]thieno[3,2-*f*][1,4]oxa-zepine-2,5-dione (15)**

Compound **15** was yielded upon the reaction of compound **4** and 4-aminophenol, formed from ethanol as crystals. Yield: 88%; m.p.: 270–371 °C; IR (KBr, cm^−1^) = 3380 (OH), 3036, (sp^2^ CH), 2970, 2889 (sp^3^ CH), 1670 (2 C=O); ^1^H NMR (400 MHz, CDCl_3_): *δ* = 7.75 (s, 1H, OH), 7.21 (d, *J* = 9.2 Hz, 2H, Ar-H), 6.80 (d, *J* = 9.2 Hz, 2H, Ar-H), 4.77 (s, 2H, oxazepine CH_2_), 2.85 (t, *J* = 8.2 Hz, 2H, cycloheptane-Hs), 2.72 (t, *J* = 7.3 Hz, 2H, cycloheptane-Hs), 1.73–1.51 (m, 6H, cycloheptane-Hs); ^13^C NMR (100 MHz, CDCl_3_): *δ* = 164.07 (C=O), 163.74 (C=O), 155.82, 151.03, 135.89, 133.79, 125.64, 124.73, 118.06, 115.72, 47.00 (oxazepine CH_2_), 31.08, 28.39, 28.19, 27.43, 25.93.; MS (EI,70 eV): *m*/*z* (%) = 343 [M**^. +^**] (18.30%), 91 (100%); Anal. Calc. for C_18_H_17_NO_4_S (343.40): C, 62.96; H, 4.99; N, 4.08; S, 9.34. Found: C, 62.98; H, 4.97; N, 4.10; S, 9.35.

**4-(4-Methoxyphenyl)-3,4,7,8,9,10-hexahydro-6*H*-cyclohepta[4,5]thieno[3,2-*f*][1,4]oxa-zepine-2,5-dione (16)**

Compound **16** was yielded upon the reaction of compound **4** and 4-methoxyaniline. Crystallized from ethanol. Yield: 89%; m.p.: 210–212 °C; IR (KBr, cm^−1^)= 3075 (sp^2^ CH), 2960, 2850 (sp^3^ CH), 1699 (2 C=O); ^1^H NMR (400 MHz, CDCl_3_): *δ* = 7.29 (d, *J* = 8.9 Hz, 2H, Ar-H), 6.87 (d, *J* = 8.7 Hz, 2H, Ar-H), 4.77 (s, 2H, oxazepine CH_2_), 3.79 (s, 3H, -OCH_3_), 2.85 (t, *J* = 8.2 Hz, 2H, cycloheptane-Hs), 2.72 (t, *J* = 7.3 Hz, 2H, cycloheptane-Hs), 1.73–1.51 (m, 6H, cycloheptane-Hs).; ^13^C NMR (100 MHz, CDCl_3_): *δ* = 164.07 (C=O), 163.74 (C=O), 155.82, 154.71, 135.89, 134.78, 125.64, 122.61, 118.06, 113.77, 54.30 (-OCH_3_), 47.00 (oxazepine CH_2_), 31.08, 28.39, 28.19, 27.43, 25.93.; MS (EI, 70 eV): *m*/*z* (%) = 357 [M**^. +^**] (28.55%), 326 (100%); Anal. Calc. for C_19_H_19_NO_4_S (357.42): C, 63.85; H, 5.36; N, 3.92; S, 8.97. Found: C, 63.88; H, 5.38; N, 3.90; S, 8.98.

### 3.2. Biology

#### 3.2.1. Induction of AD Rat Models

All procedures were conducted conforming to the applicable National Institutes of Health standards and regulations for the Care and Use of Laboratory Animals. In this experiment, adult male Dawley rats in excellent condition weighing between 300 and 320 g were used. The animals were acquired from the Nile Company for Pharmaceuticals and Chemical Industries (Cairo, Egypt). They were placed in a controlled laboratory setting with unrestricted access to water, a temperature range of 24 to 26 °C, and 12 h a light–dark cycle. Three rats per cage were housed in polycarbonate cages that were individually packed with paper and encased with stainless steel wire [90].

#### 3.2.2. Experimental Design

See Supplementary Information [90,91,92].

#### 3.2.3. Behavioral Tests

##### MWM Test

Spatial learning and memory were inspected using the MWM test [93]. Tap water was poured into a 150 cm diameter by 60 cm high circular water tank until it reached a depth of 30 cm (25 ± 2 °C). To make the water translucent, non-toxic white dye was applied. The east, west, north, and south quadrants of the pool were essentially separated into four equal sections. At a specific spot in the middle of one quadrant, an escape platform with a diameter of 10 cm was submerged 2 cm below the water’s surface. The platform stood in the exact same quadrant throughout the testing. The rats’ swimming route was recorded by a video camera situated above the pool. From a designated spot in each quadrant, each rat was submerged in the water with its back turned to the pool wall, and it was then left to swim to the platform. The rats received training sessions every day for three days in a row, with four trials conducted in each session. Before the next trial began, the animals were kept a maximum of 60 s to explore the hidden platform. After that, they were permitted to relax on it for 20 s. The rat was positioned gently on the platform and allowed 20 s to rest if it took longer than 60 s to locate it. The time taken to find the platform, or the escape latency, was documented. In order to do a probing test on the fourth day, the platform was detached away, and the rats were given 60 s to swim liberally. The time spent in the assigned quadrant was counted [93].

##### Y-Maze SAP Test

One kind of short-term memory that SAP can represent is spatial working memory. Three arms, designated A, B, or C, were employed in a black hardwood Y-maze with a symmetrical triangular core space. Rats were briefly positioned at the edge of one arm and given 8 min to freely navigate around the maze. When the rat’s rear paws were fully inside the arm, the entries were counted. The following formula was used to determine SAP based on the total number of arm entries and alternations: SAP (%) = [number of alternations/(total arm entries − 2)] × 100 [94].

#### 3.2.4. Tissue Sampling and Preparation

The brain tissues of the rats were removed and rigorously washed in isotonic saline 24 h after they were euthanized following the last behavioral test. For histological investigation, four brains per group were preserved for the whole night in 10% neutral buffered formalin. The remaining six brains were split into two sections each. In order to create a 10% homogenate (*w*/*v*), the first component was instantaneously homogenized using an ice-cold solution that included 300 mM sucrose and 50 mM Tris-HCl (pH 7.4). The homogenate was centrifuged at 1800× *g* for 10 min at 4 °C for biochemical tests, and the supernatant was thereafter kept at −20 °C. The second portion was set aside for use in RT-PCR analysis at −80 °C [94].

#### 3.2.5. Biochemical Measurements

##### Fluorometric Technique

Brain monoamine levels were promptly assessed following the rats’ euthanasia to prevent changes in the substance’s concentration. Dopamine (DA), norepinephrine (NE), and serotonin (5-HT) fluorometric tests were conducted on brain tissue homogenate using the Ciarlone method. The process involved oxidizing monoamines to their adrenochromes, rearranging them to their adrenolutins, and then fluorometrically detecting them using samples at λ_ex_/λ_em_ = 385/485 nm, 320/385 nm, and 360/470 nm for NE, DA, and 5-HT, respectively. The concentrations were articulated as nanograms per gram of fresh tissue using the fluorescence of reference solutions [95].

##### Colorimetric Technique

MDA was evaluated using the thiobarbituric acid method at a wavelength of 532 nm in order to colorimetrically detect the degree of lipid peroxidation in brain homogenate. The SOD enzyme activity was estimated using the Marklund et al. method, which is founded on the enzyme’s capacity to decrease the nitro blue tetrazolium dye at a wavelength of 540 nm [96]. To evaluate the TAC, a colorimetric approach was employed to estimate the amount of hydrogen peroxide that remains after 3,5-dichloro-2-hydroxybenzene sulphonate is converted to a colored product at 660 nm [96]. Additionally, a colorimetric measurement was made at 412 nm to assess the amount of AChE in the brain tissue homogenate [97].

##### ELISA Technique

See Supplementary Information [98].

##### Real-Time Quantitative Polymerase Chain Reaction (RT-qPCR)

See Supplementary Information [91].

##### AChE Inhibition Assay

All newly created compounds were subjected to an AChE enzyme inhibitory screening. This was performed with the Colorimetric AChE Inhibitor Screening Kit (BioVision Catalog # K197-100). The 96-well assay kit provides a rapid, simple, and reliable way to screen for AChE inhibitors in large quantities. The candidate inhibitors were dissolved at 100X or higher and then further diluted to a concentration of 20× using the AChE test buffer. A completely transparent 96-well plate with a flat bottom was loaded with 10 µL of test inhibitors that were diluted (20×). Assay buffer applied to dilute 2 μL of the stock (10 mM) solution to 100 μL in order to create the inhibitor control (donepezil). Assay buffer was then applied to further dilute the donepezil solution to 40 μL. Ten microliters of the 20 μM donepezil working solution were added to the chosen well. After being reconstructed, AChE was diluted 25 times. A measure of 10 μL of diluted AChE was applied to each well that contained compounds, inhibitor control, and solvent control. A 12-fold dilution of the AChE substrate was made. Measures of 25 μL of AChE assay buffer, 5 μL of probe mix, and 10 μL of diluted AChE substrate were mixed to create the reaction mix. The reaction mix was mixed with the compound, enzyme control, and inhibitor control. After 40 min at room temperature in kinetic mode, the absorbance (OD) at a wavelength of 412 nm was measured using a temperature-controlled plate reader. In a control test, DMSO was utilized as the solvent and was anticipated to have 100% enzyme activity. The IC_50_ was calculated using nonlinear regression analysis using the response–concentration (log) curve [99].

##### BChE Inhibition Assay

Using an Invitrogen BChE test kit (Catalog #EIABCHEF), the assay was performed in compliance with the supplier’s instructions. The study was conducted using black 96-well plates. A measure of 100 μL of each diluted tested chemical or tacrine was added to the 96-well plate. Each well was then filled with 50 μL of the reaction mixture, which consisted of fluorescence detection reagent and butyrylthiocholine iodide in assay buffer. The plate was then left to incubate for 20 min at room temperature. Finally, excitation at 390 nm was used to observe emission at 510 nm using a microplate reader. Background fluorescence was further eliminated for each data point. In a single run, the assay was conducted in triplicate [100,101].

#### 3.2.6. Statistical Analysis

The Tukey–Kramer test was used for post hoc analysis after the one-way ANOVA was used for multiple comparisons. The findings are shown as mean ± SEM, and a p-value of less than 0.05 was deemed statistically significant. The GraphPad Prism software (version 8, ISI^®^, San Diego, CA, USA) was used in order to perform statistical analysis and to create the graphs.

#### 3.2.7. Histopathology of Brain Tissue

Brain tissue samples were stationed in 10% formalin for 24 h, washed with water, and then serially diluted with alcohol to induce dehydration. After being submerged in paraffin, the specimens were cut into segments that were 4 µm thick using a microtome. After being collected on glass slides, the tissue samples were deparaffinized and stained with eosin and hematoxylin in order to conduct a standard histological examination under a light microscope [102,103].

### 3.3. In Silico ADME and Toxicity Prediction

The SwissADME online tool was used to analyze the synthetic compounds in this investigation as well as the traditional reference medications tacrine and donepezil (http://www.sib.swiss, accessed on 1 February 2025). Investigations were conducted on the in silico ADME’s properties and drug-likeness [104]. We used ChemDraw 19.0 to transform the structures into SMILES databases. These SMILES were submitted to the SwissADME website, which evaluated the physicochemical traits, pharmacokinetic attributes, ADME parameters, and compatibility with medicinal chemistry [105,106]. BBB permeability was predicted using the ADMETLab3.0 webtool (https://admetlab3.scbdd.com/) [107,108].

Based on online resources, ProTox-II (https://tox-new.charite.de/protox_II) and pkCSM (http://biosig.unimelb.edu.au/pkcsm/prediction), the toxicity of the top hits, **2**, **14**, and **16**, as well as tacrine and donepezil, were obtained [88,89].

### 3.4. Molecular Docking

The 3D crystal structures of AChE (PDB ID: 4EY7) and BChE (PDB ID: 4BDS) were retrieved from the RCSB PDB (https://www.rscb.org) in pdb format [109,110]. The protein structure was introduced in the Molecular Operating Environment (Montreal, QC, Canada) (MOE 2014.09) [111]. The MMFF94x forcefield was set before the simulation study. The imported proteins were prepared by rectifying usual errors, such as adding missing H atoms and minimizing energy [112,113,114]. All water molecules were eliminated from the structure. The structures of the compounds were sketched and imported into a database. The triangle matcher was utilized for placement, while London dG was employed as a scoring function throughout the docking procedure [115]. The Rigid Receptor approach was utilized for refining [116,117].

## 4. Conclusions

This study assessed the anti-AD effects of a new class of tricyclic and bicyclic thieno-oxazepine hybrids by contrasting them with donepezil and tacrine, respectively, as AChE and BChE suppressors. Compounds **14** and **16** were found to be the most potent AChE inhibitors, with IC_50_ values of 0.39 µM and 0.76 µM, respectively. Additionally, with an IC_50_ value of 0.70 µM, compound **15** revealed exceptional BChE inhibitory activity on parity with tacrine. Only the developed hybrids **13** and **15**, with respective values of 0.84 and 0.44, showed more selectivity towards BChE. In addition to other biomarkers, the rat brains were examined for TAC, SOD, MDA, BDNF, NFκ*β*, TLR4, IL-*β*, and TNF-*α*. We found that our compounds elevated antioxidant biomarkers and reduced inflammatory signs. By enhancing Bcl-2 levels and lowering BAX levels, all of the deigned hybrids showed their ability to reduce brain cell death. Each of the deigned hybrids presented promise in enhancing Wnt pathway activity, which in turn enhanced the proliferation of brain cells. These results suggest that the deigned hybrids are multipotent lead molecules that may be used to develop novel anti-AD drugs.

## Figures and Tables

**Table 1:** The impact of 2 and 4 on memory, learning and SAP%.

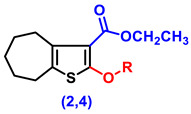				
Cpd. No.	R	Memory (s)	Learning (s)	SAP%
**Control**	-	53.67 ± 1.41	8 ± 0.58	90.83 ± 1.01
**AlCl_3_-induced AD**	-	9.83 ± 1.49 ^a^	49.17 ± 2.63 ^a^	47.67 ± 1.48 ^a^
**Donepezil**	-	42.67 ± 0.61 ^ab^	14.78 ± 0.83 ^ab^	85 ± 0.58 ^ab^
**2**	H	24.17 ± 1.22 ^abc^	33.33 ± 0.8 ^abc^	62.33 ± 1.05 ^abc^
**4**	-CO-CH_2_-Cl	26.67 ± 1.54 ^abc^	31.5 ± 1.48 ^abc^	72.67 ± 1.02 ^abc^

**Note:** Significance (*p* < 0.05) relative to (^a^) control group, (^b^) AlCl_3_-induced AD group, and (^c^) donepezil (data ± SE; n = 6).

**Table 2:** The effect of (**11**–**16)** on memory (s), learning (s), and SAP%.

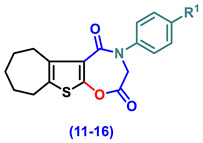				
Cpd. No.	R^1^	Memory (s)	Learning (s)	SAP%
**Control**	-	53.67 ± 1.41	8 ± 0.58	90.83 ± 1.01
**AlCl_3_-induced AD**	-	9.83 ± 1.49 ^a^	49.17 ± 2.63 ^a^	47.67 ± 1.48 ^a^
**Donepezil**	-	42.67 ± 0.61 ^ab^	14.78 ± 0.83 ^ab^	85 ± 0.58 ^ab^
**11**	H	35.17 ± 0.95 ^abc^	25.17 ± 1.25 ^abc^	69.67 ± 1.69 ^abc^
**12**	Cl	37.17 ± 1.52 ^abc^	23.5 ± 1.23 ^abc^	76.5 ± 1.52 ^abc^
**13**	CN	40.50 ± 2.62 ^abc^	14.17 ± 0.95 ^abc^	82.67 ± 1.28 ^abc^
**14**	-OCH_2_CH_3_	29.50 ± 2 ^abc^	21 ± 1.13 ^abc^	77.5 ± 2.11 ^abc^
**15**	OH	42.50 ± 1.06 ^abc^	19.17 ± 1.01 ^abc^	82.5 ± 0.89 ^abc^
**16**	-OCH_3_	36.00 ± 1.27 ^abc^	26.83 ± 1.2 ^abc^	71.67 ± 1.15 ^abc^

**Note:** Significance (*p* < 0.05) relative to (^a^) control group, (^b^) AlCl_3_-induced AD group, and (^c^) donepezil (data ± SE; n = 6).

**Table 3:** Effects of **2** and **4** on BACE1 (ng/mL) and amyloid-*β*(ng/g tissue).

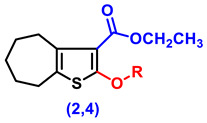			
Cpd. No.	R	BACE1 (ng/mL)	Amyloid-*β* (ng/g Tissue)
Control	-	1.01 ± 0.01	1.59 ± 0.03
AlCl_3_-induced AD	-	29.68 ± 0.2 ^a^	27.92 ± 0.32 ^a^
Donepezil	-	4.93 ± 0.04 ^ab^	8.10 ± 1.71 ^ab^
**2**	H	23.60 ± 0.2 ^abc^	19.52 ± 0.15 ^abc^
**4**	-CO-CH_2_-Cl	21.89 ± 0.27 ^abc^	17.05 ± 0.2 ^abc^

**Note:** Significance (*p* < 0.05) relative to (^a^) control group, (^b^) AlCl_3_-induced AD group, and (^c^): donepezil (data ± SE; n = 6).

**Table 4:** Effects of (**11**–**16)** on BACE1(ng/mL) and amyloid-*β*(ng/g tissue).

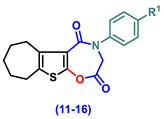			
Cpd. No.	R^1^	BACE1 (ng/mL)	Amyloid-*β* (ng/g Tissue)
Control	-	1.01 ± 0.01	1.59 ± 0.03
AlCl_3_-induced AD	-	29.68 ± 0.2 ^a^	27.92 ± 0.32 ^a^
Donepezil	-	4.93 ± 0.04 ^ab^	8.10 ± 1.71 ^ab^
**11**	H	18.83 ± 0.13 ^abc^	14.25 ± 0.16 ^abc^
**12**	Cl	13.68 ± 0.2 ^abc^	10.51 ± 0.1 ^abc^
**13**	CN	11.35 ± 0.31 ^abc^	9.04 ± 0.9 ^abc^
**14**	-OCH_2_CH_3_	16.66 ± 0.38 ^abc^	11.66 ± 0.17 ^abc^
**15**	OH	10.50 ± 0.2 ^abc^	7.46 ± 0.9 ^abc^
**16**	-OCH_3_	17.44 ± 0.23 ^abc^	12.44 ± 0.14 ^abc^

**Note:** Significance (*p* < 0.05) relative to (^a^) control group, (^b^) AlCl_3_-induced AD group, and (^c^) donepezil (data ± SE; n = 6).

**Table 5:** Effect of **2** and **4** on 5-HT (ng/g tissue), DA (ng/g tissue), and NE (nmol/g tissue). BDNF (ng/mL) and AChE (ng/g tissue) levels in brain tissues compared to donepezil and negative control.

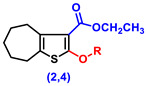						
Cpd. No.	R	5-HT (ng/g Tissue)	DA (ng/g Tissue)	NE (nmol/g Tissue)	BDNF (ng/mL)	AChE (ng/g Tissue)
**Control**	-	13.59 ± 0.22	64.73 ± 0.36	730.6 ± 0.36	165.5 ± 0.14	11.93 ± 0.26
**AlCl_3_-induced AD**	-	1.887 ± 0.49 ^a^	11.84 ± 1.42 ^a^	185.7 ± 1.42 ^a^	42.56 ± 0.04 ^a^	67.34 ± 0.22 ^a^
**Donepezil**	-	11.23 ± 0.41 ^ab^	58.12 ± 14.01 ^ab^	635.4 ± 14.01 ^ab^	122.4 ± 0.48 ^ab^	13.91 ± 0.42 ^b^
**2**	H	3.16 ± 0.63 ^abc^	24.71 ± 1.62 ^abc^	256.9 ± 1.62 ^abc^	64.95 ± 0.01 ^abc^	51.41 ± 0.13 ^abc^
**4**	-CO-CH_2_-Cl	4.29 ± 0.76 ^abc^	30.69 ± 1.2 ^abc^	282.6 ± 1.2 ^abc^	75.75 ± 0.01 ^abc^	43.89 ± 0.19 ^abc^

**Note:** Significance (*p* < 0.05) relative to (^a^) control group, (^b^) AlCl_3_-induced AD group, and (^c^) donepezil (data ± SE; n = 6).

**Table 6:** Effect of (**11**–**16)** on 5-HT (ng/g tissue), DA (ng/g tissue), and NE (nmol/g tissue). BDNF (ng/mL) and AChE (ng/g tissue) levels in brain tissues compared to donepezil and negative control.

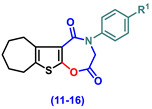						
Cpd. No.	R^1^	5-H (ng/g Tissue)	DA (ng/g Tissue)	NE (nmol/g Tissue)	BDNF (ng/mL)	AChE (ng/g Tissue)
**Control**	-	13.59 ± 0.22	64.73 ± 0.36	730.6 ± 0.36	165.5 ± 0.14	11.93 ± 0.26
**AlCl_3_-induced AD**	-	1.887 ± 0.49 ^a^	11.84 ± 1.42 ^a^	185.7 ± 1.42 ^a^	42.56 ± 0.04 ^a^	67.34 ± 0.22 ^a^
**Donepezil**	-	11.23 ± 0.41 ^ab^	58.12 ± 0.14 ^ab^	635.4 ± 14.01 ^ab^	122.4 ± 0.48 ^ab^	13.91 ± 0.42 ^ab^
**11**	H	9.13 ± 0.36 ^abc^	54.63 ± 6.11 ^abc^	321.2 ± 6.11 ^abc^	127.3 ± 0.01 ^abc^	20.09 ± 0.45 ^abc^
**12**	Cl	5.07 ± 0.18 ^abc^	38.37 ± 2.86 ^abc^	420.5 ± 2.86 ^abc^	83.62 ± 0.06 ^abc^	35.66 ± 0.19 ^abc^
**13**	CN	7.39 ± 0.1 ^abc^	48.65 ± 2.3 ^abc^	449.6 ± 2.3 ^abc^	104.9 ± 0.07 ^abc^	24.35 ± 0.8 ^abc^
**14**	-OCH_2_CH_3_	8.22 ± 0.19 ^abc^	51.88 ± 2.66 ^abc^	386.7 ± 2.66 ^abc^	115.8 ± 0.07 ^abc^	19.4 ± 0.39 ^abc^
**15**	OH	6.77 ± 0.22 ^abc^	44.92 ± 2.85 ^abc^	499.8 ± 2.85 ^abc^	94.48 ± 0.05 ^abc^	25.73 ± 0.37 ^abc^
**16**	-OCH_3_	10.65 ± 0.71 ^abc^	52.96 ± 1.52 ^abc^	366.4 ± 1.52 ^abc^	134.8 ± 0.01 ^abc^	16.83 ± 0.29 ^abc^

**Note:** Significance (*p* < 0.05) relative to (^a^) control group, (^b^) AlCl_3_-induced AD group, and (^c^) donepezil (data ± SE; n = 6).

**Table 7:** Effect of **2** and **4** on TNF-*α* (pg/g tissue) and IL-*β* (pg/g tissue), along with mRNA expression levels of TLR4, NFκ*β*, and NLRP3.

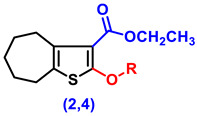						
Cpd. No.	R	TNF-*α* (pg/g Tissue)	IL-*β* (pg/g Tissue)	TLR4	NFκβ	NLRP3
**Control**	-	26.75 ± 0.16	31.3 ± 0.28	1.02 ± 0.01	1.07 ± 0.01	1.04 ± 0.004
**AlCl_3_-induced AD**	-	176 ± 1.05 ^a^	118.4 ± 0.5 ^a^	9.63 ± 0.08 ^a^	8.48 ± 0.15 ^a^	10.68 ± 0.09 ^a^
**Donepezil**	-	61.7 ± 1.12 ^ab^	58.75 ± 0.99 ^ab^	2.72 ± 0.02 ^ab^	3.0 ± 0.05 ^ab^	2.90 ± 0.04 ^ab^
**2**	H	121.4 ± 0.97 ^abc^	93.93 ± 1.26 ^abc^	7.66 ± 0.12 ^abc^	6.53 ± 0.08 ^abc^	8.29 ± 0.09 ^abc^
**4**	-CO-CH_2_-Cl	113.6 ± 0.83 ^abc^	82.82 ± 1.1 ^abc^	6.39 ± 0.12 ^abc^	5.77 ± 0.19 ^abc^	6.30 ± 0.09 ^abc^

**Note:** Significance (*p* < 0.05) relative to (^a^) control group, (^b^) AlCl_3_-induced AD group, and (^c^) donepezil (data ± SE; n = 6).

**Table 8:** Effect of (**11**–**16)** on TNF-*α* (pg/g tissue) and IL-*β* (pg/g tissue), in addition to mRNA expression levels of TLR4, NFκ*β*, and NLRP3.

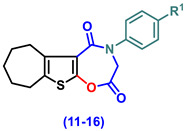						
Cpd. No.	R^1^	TNF-*α* (pg/g Tissue)	IL-*β* (pg/g Tissue)	TLR4	NFκβ	NLRP3
**Control**	-	26.75 ± 0.16	31.3 ± 0.28	1.02 ± 0.01	1.07 ± 0.01	1.04 ± 0.004
**AlCl_3_-induced AD**	-	176 ± 1.05 ^a^	118.4 ± 0.5 ^a^	9.63 ± 0.08 ^a^	8.48 ± 0.15 ^a^	10.68 ± 0.09 ^a^
**Donepezil**	-	61.7 ± 1.12 ^ab^	58.75 ± 0.99 ^ab^	2.72 ± 0.02 ^ab^	3.0 ± 0.05 ^ab^	2.90 ± 0.04 ^ab^
**11**	H	99.23 ± 0.42 ^abc^	68.56 ± 0.54 ^abc^	5.56 ± 0.1 ^abc^	4.93 ± 0.08 ^abc^	5.39 ± 0.11 ^abc^
**12**	Cl	65.98 ± 0.91 ^abc^	54.51 ± 1.3 ^abc^	2.75 ± 0.06 ^abc^	4.30 ± 0.14 ^abc^	3.21 ± 0.12 ^abc^
**13**	CN	52.38 ± 0.59 ^abc^	45.79 ± 1.34 ^abc^	4.09 ± 0.27 ^abc^	3.49 ± 0.07 ^abc^	2.86 ± 0.11 ^abc^
**14**	-OCH_2_CH_3_	73.21 ± 0.58 ^abc^	59.92 ± 0.98 ^abc^	3.60 ± 0.12 ^abc^	3.73 ± 0.04 ^abc^	3.92 ± 0.04 ^abc^
**15**	OH	44.6 ± 0.6 ^abc^	45.66 ± 1.03 ^abc^	4.57 ± 0.09 ^abc^	2.98 ± 0.10 ^abc^	3.36 ± 0.12 ^abc^
**16**	-OCH_3_	85.17 ± 0.46 ^abc^	53.04 ± 0.61 ^abc^	4.43 ± 0.08 ^abc^	4.70 ± 0.12 ^abc^	4.32 ± 0.10 ^abc^

**Note:** Significance (*p* < 0.05) relative to (^a^) control group, (^b^) AlCl_3_-induced AD group, and (^c^) donepezil (data ± SE; n = 6).

**Table 9:** Effect of **2** and **4** on Caspase-1, BAX, and Bcl-2 gene expression.

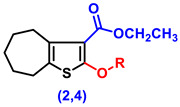					
Cpd. No.	R	Caspase-1	BAX	Bcl-2	BAX/Bcl-2 Ratio
**Control**	-	1.02 ± 0.003	1.01 ± 0.004	1.08 ± 0.006	0.94
**AlCl_3_-induced AD**	-	9.52 ± 0.009 ^a^	9.68 ± 0.160 ^a^	0.12 ± 0.002 ^a^	80.67
**Donepezil**	-	2.92 ± 0.050 ^ab^	2.25 ± 0.058 ^ab^	0.98 ± 0.002 ^ab^	2.30
**2**	H	7.46 ± 0.120 ^abc^	8.13 ± 0.070 ^abc^	0.27 ± 0.007 ^abc^	30.11
**4**	-CO-CH_2_-Cl	6.46 ± 0.120 ^abc^	7.42 ± 0.030 ^abc^	0.38 ± 0.020 ^abc^	19.53

**Note:** Significance (*p* < 0.05) relative to (^a^) control group, (^b^) AlCl_3_-induced AD group, and (^c^) donepezil (data ± SE; n = 6).

**Table 10:** Effects of compounds **11**–**16** on Caspase-1, BAX, and Bcl-2 gene expression.

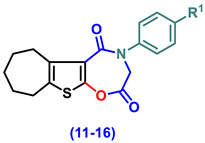					
Cpd. No.	R^1^	Caspase-1	BAX	Bcl-2	BAX/Bcl-2 Ratio
**Control**	-	1.02 ± 0.003	1.01 ± 0.004	1.08 ± 0.006	0.94
**AlCl_3_-induced AD**	-	9.52 ± 0.009 ^a^	9.68 ± 0.160 ^a^	0.12 ± 0.002 ^a^	80.67
**Donepezil**	-	2.92 ± 0.050 ^ab^	2.25 ± 0.0580 ^ab^	0.98 ± 0.002 ^ab^	2.30
**11**	H	5.92 ± 0.040 ^abc^	6.84 ± 0.090 ^abc^	0.52 ± 0.020 ^abc^	13.15
**12**	Cl	4.54 ± 0.090 ^abc^	4.85 ± 0.110 ^abc^	0.82 ± 0.020 ^abc^	5.91
**13**	CN	3.85 ± 0.070 ^abc^	5.80 ± 0.120 ^abc^	0.79 ± 0.020 ^abc^	7.34
**14**	-OCH_2_CH_3_	5.08 ± 0.050 ^abc^	5.49 ± 0.100 ^abc^	0.70 ± 0.004 ^abc^	7.84
**15**	OH	2.94 ± 0.110 ^abc^	4.47 ± 0.040 ^abc^	0.86 ± 0.020 ^abc^	5.20
**16**	-OCH_3_	5.04 ± 0.130 ^abc^	6.14 ± 0.090 ^abc^	0.63 ± 0.020 ^abc^	9.75

**Note:** Significance (*p* < 0.05) relative to (^a^) control group, (^b^) AlCl_3_-induced AD group, and (^c^) donepezil (data ± SE; n = 6).

**Table 11:** Effects of **2** and **4** on Wnt3/*β*-catenin/GSK-3*β* signaling.

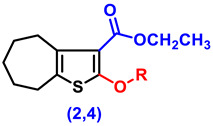				
Cpd. No.	R	Wnt3a (ng/g Tissue)	GSK3*β*	*β*-Catenin (ng/L)
**Control**	-	1.01 ± 0.003	1.01 ± 0.004	16.94 ± 0.14
**AlCl_3_-induced AD**	-	0.12 ± 0.003 ^a^	10.20 ± 0.03 ^a^	1.578 ± 0.09 ^a^
**Donepezil**	-	0.98 ± 0.01 ^ab^	2.05 ± 0.07 ^ab^	12.92 ± 0.48 ^ab^
**2**	H	0.25 ± 0.01 ^abc^	8.80 ± 0.12 ^abc^	4.262 ± 0.12 ^abc^
**4**	-CO-CH_2_-Cl	0.34 ± 0.01 ^abc^	6.86 ± 0.15 ^abc^	6.17 ± 0.07 ^abc^

**Note:** Significance (*p* < 0.05) relative to (^a^) control group, (^b^) AlCl_3_-induced AD group, and (^c^) donepezil (data ± SE; n = 6).

**Table 12:** Effects of compounds (**11**–**16)** on Wnt3/*β*-catenin/GSK-3*β* signaling.

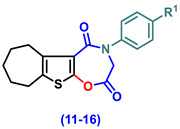				
Cpd. No.	R^1^	Wnt3a (ng/g Tissue)	GSK3*β*	*β*-Catenin (ng/L)
**Control**	-	1.01 ± 0.003	1.01 ± 0.004	16.94 ± 0.14
**AlCl_3_-induced AD**	-	0.12 ± 0.003 ^a^	10.20 ± 0.03 ^a^	1.578 ± 0.09 ^a^
**Donepezil**	-	0.98 ± 0.01 ^ab^	2.05 ± 0.07 ^ab^	12.92 ± 0.48 ^ab^
**11**	H	0.47 ± 0.01 ^abc^	2.34 ± 0.06 ^abc^	7.25 ± 0.04 ^abc^
**12**	Cl	0.76 ± 0.01 ^abc^	5.36 ± 0.07 ^abc^	9.91 ± 0.15 ^abc^
**13**	CN	0.84 ± 0.01 ^abc^	3.63 ± 0.04 ^abc^	11.67 ± 0.13 ^abc^
**14**	-OCH_2_CH_3_	0.66 ± 0.02 ^abc^	3.13 ± 0.07 ^abc^	8.4 ± 0.09 ^abc^
**15**	OH	0.88 ± 0.01 ^abc^	4.72 ± 0.05 ^abc^	13.86 ± 0.11 ^abc^
**16**	-OCH_3_	0.52 ± 0.01 ^abc^	2.18 ± 0.10 ^abc^	7.34 ± 0.12 ^abc^

**Note:** Significance (*p* < 0.05) relative to (^a^) control group, (^b^) AlCl_3_-induced AD group, and (^c^) donepezil (data ± SE; n = 6).

**Table 13:** Effect of **2** and **4** on SOD, TAC, and MDA levels, as well as Nrf-2 and HO-1 mRNA expression in brain tissues compared to donepezil and negative control.

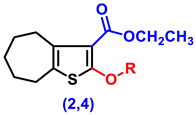						
Cpd. No.	R	SOD(U/g)	TAC (µmol/g Tissue)	MDA (nmol/g Tissue)	Nrf-2	HO-1
**Control**	-	3.052 ± 0.4	46.68 ± 0.05	7.105 ± 0.03	1.07 ± 0.006	1.02 ± 0.003
**AlCl_3_-induced AD**	-	0.38 ± 0.38 ^a^	9.55 ± 0.250 ^a^	116.3 ± 0.07 ^a^	0.16 ± 0.020 ^a^	0.12 ± 0.007 ^a^
**Donepezil**	-	5.78 ± 0.97 ^ab^	34.88 ± 1.510 ^ab^	25.25 ± 0.35 ^ab^	0.82 ± 0.001 ^ab^	0.81 ± 0.002 ^ab^
**2**	H	0.68 ± 0.26 ^abc^	18.26 ± 0.395 ^abc^	92.07 ± 0.04 ^abc^	0.25 ± 0.008 ^abc^	0.34 ± 0.032 ^abc^
**4**	-CO-CH_2_-Cl	1.75 ± 0.31 ^abc^	25.87 ± 0.965 ^abc^	77.14 ± 0.06 ^abc^	0.31 ± 0.004 ^abc^	0.39 ± 0.007 ^abc^

**Note:** Significance (*p* < 0.05) relative to (^a^) control group, (^b^) AlCl_3_-induced AD group, and (^c^) donepezil (data ± SE; n = 6).

**Table 14:** Effect of (**11**–**16)** on SOD, TAC, and MDA levels, as well as Nrf-2 and HO-1 mRNA expression in brain tissues compared to donepezil and negative control.

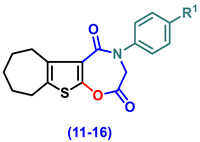						
Cpd. No.	R^1^	SOD(U/g)	TAC (µmol/g Tissue)	MDA (nmol/g Tissue)	Nrf-2	HO-1
**Control**	-	3.05 ± 0.4	46.68 ± 0.05	7.10 ± 0.03	1.07 ± 0.006	1.02 ± 0.003
**AlCl_3_-induced AD**	-	0.38 ± 0.38 ^a^	9.55 ± 0.25 ^a^	116.3 ± 0.07 ^a^	0.16 ± 0.02 ^a^	0.12 ± 0.007 ^a^
**Donepezil**	-	5.78 ± 0.97 ^ab^	34.88 ± 1.51 ^ab^	25.25 ± 0.35 ^ab^	0.82 ± 0.01 ^ab^	0.81 ± 0.002 ^ab^
**11**	H	2.46 ± 0.32 ^abc^	38.23 ± 0.50 ^abc^	36.75 ± 0.09 ^abc^	0.35 ± 0.006 ^abc^	0.53 ± 0.004 ^abc^
**12**	Cl	2.15 ± 0.39 ^abc^	29.33 ± 0.57 ^abc^	72.02 ± 0.05 ^abc^	0.64 ± 0.020 ^abc^	0.69 ± 0.020 ^abc^
**13**	CN	2.53 ± 0.25 ^abc^	32.9 ± 1.22 ^abc^	52.58 ± 0.08 ^abc^	0.78 ± 0.010 ^abc^	0.75 ± 0.010 ^abc^
**14**	OCH_2_CH_3_	2.38 ± 0.78 ^abc^	36.96 ± 0.51 ^abc^	47.6 ± 0.12 ^abc^	0.50 ± 0.020 ^abc^	0.64 ± 0.009 ^abc^
**15**	OH	2.56 ± 0.54 ^abc^	31.67 ± 0.42 ^abc^	63.68 ± 0.12 ^abc^	0.82 ± 0.020 ^abc^	0.82 ± 0.005 ^abc^
**16**	OCH_3_	2.92 ± 0.20 ^abc^	42.42 ± 0.84 ^abc^	31.63 ± 0.05 ^abc^	0.47 ± 0.005 ^abc^	0.50 ± 0.020 ^abc^

**Note:** Significance (*p* < 0.05) relative to (^a^) control group, (^b^) AlCl_3_-induced AD group, and (^c^) donepezil (data ± SE; n = 6).

**Table 15:** IC_50_ values and Selectivity Index (SI) of **2** and **4** against AChE/BChE vs. donepezil and tacrine.

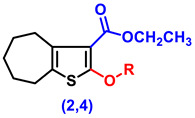				
Cpd. No.	R	IC_50_(µM)		Selectivity Index BChE/AChE
		AChE	BChE	
**2**	H	1.10 ± 0.04	5.72 ± 0.20	5.21
**4**	-CO-CH_2_-Cl	41.22 ± 1.42	56.19 ± 1.93	1.36
**Donepezil**	-	0.22 ± 0.01	-	-
**Tacrine**	-	-	0.18 ± 0.01	-

**Table 16:** IC_50_ values and Selectivity Index (SI) of compounds (**11**–**16)** against AChE/BChE vs. donepezil and tacrine.

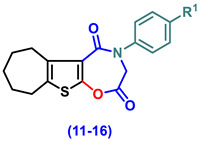				
Cpd. No.	R^1^	IC_50_ (µM)		Selectivity Index BChE/AChE
		AChE	BChE	
**11**	H	2.67 ± 0.09	13.85 ± 0.48	5.18
**12**	Cl	10.90 ± 0.38	65.86 ± 2.27	6.04
**13**	CN	5.04 ± 0.17	4.22 ± 0.14	0.84
**14**	-OCH_2_CH_3_	0.39 ± 0.01	2.93 ± 0.10	7.50
**15**	OH	1.59 ± 0.06	0.70 ± 0.02	0.44
**16**	-OCH_3_	0.76 ± 0.03	5.23 ± 0.18	6.89
**Donepezil**	-	0.22 ± 0.01	-	-
**Tacrine**	-	-	0.18 ± 0.01	-

**Table 17:** Physicochemical attributes of synthetic hybrids, donepezil, and tacrine.

Molecule	#H-Bond Donors	#H-Bond Acceptors	MLogP	MW	PAINS Alerts	Synthetic Accessibility
**2**	1	3	2.17	240.32	0	3.14
**4**	0	4	2.83	316.80	0	3.48
**11**	0	3	3.18	327.40	0	3.34
**12**	0	3	3.68	361.84	0	3.33
**13**	0	4	2.51	352.41	0	3.38
**14**	0	4	3.08	371.45	0	3.58
**15**	1	4	2.62	343.40	0	3.37
**16**	0	4	2.85	357.42	0	3.47
**Donepezil**	0	4	3.06	379.49	0	3.36
**Tacrine**	1	1	2.33	198.26	0	2.08

**Table 18:** TPSA and determined percentage of ABS for generated compounds compared to donepezil and tacrine.

Cpd. No.	TPSA (Å^2^)	%ABS
**2**	80.840	81.110
**4**	80.840	81.110
**11**	57.780	89.060
**12**	57.780	89.060
**13**	67.010	85.880
**14**	67.010	85.880
**15**	57.780	89.060
**16**	57.780	89.060
**Donepezil**	38.770	95.620
**Tacrine**	38.910	95.580

**Table 19:** ADME/pharmacokinetic attributes of the hybrids investigated.

Cpd. No	BBB Perm-eant	GI Abs	P-gp Substrate	CYP1A2 Inhibitor	CYP2C19 Inhibitor	CYP2C9 Inhibitor	CYP2D6 Inhibitor	CYP3A4 Inhibitor	Log K_p_
**2**	0.199	High	−	+	+	+	−	−	−4.84
**4**	0.112	High	−	+	+	+	−	−	−5.18
**11**	0.382	High	−	+	+	+	−	+	−5.26
**12**	0.824	High	−	+	+	+	−	−	−5.02
**13**	0.017	High	−	+	+	+	−	+	−5.61
**14**	0.002	High	−	+	+	+	+	+	−5.29
**15**	0.027	High	−	+	+	+	+	+	−5.60
**16**	0.027	High	−	+	+	+	+	+	−5.46
**Donepezil**	0.353	High	+	−	−	−	+	+	−5.58
**Tacrine**	0.977	High	+	+	−	−	−	+	−5.59

**Table 20:** Drug-likeness of the investigated hybrids.

Cpd. No.	Lipinski	Veber	Ghose	Muegge	Egan	Bioavailability Score
**2**	Yes	Yes	Yes	Yes	Yes	0.55
**4**	Yes	Yes	Yes	Yes	Yes	0.55
**11**	Yes	Yes	Yes	Yes	Yes	0.55
**12**	Yes	Yes	Yes	Yes	Yes	0.55
**13**	Yes	Yes	Yes	Yes	Yes	0.55
**14**	Yes	Yes	Yes	Yes	Yes	0.55
**15**	Yes	Yes	Yes	Yes	Yes	0.55
**16**	Yes	Yes	Yes	Yes	Yes	0.55
**Donepezil**	Yes	Yes	Yes	Yes	Yes	0.55
**Tacrine**	Yes	Yes	Yes	No	Yes	0.55

**Table 21:** Toxicity of hits **2**, **14**, and **16** versus donepezil and tacrine.

pkCSM					
Test	2	14	16	Donepezil	Tacrine
**AMES toxicity**	No	No	Yes	No	Yes
**Max. tolerated dose (human)**	0.668	−0.116	−0.18	−0.217	0.55
**hERG I inhibition**	No	No	No	No	No
**hERG II inhibition**	No	No	No	Yes	No
**LD_50_**	2.201	2.666	2.762	2.753	2.33
**LOAEL**	1.884	2.048	2.113	0.991	1.204
**Hepatotoxicity**	No	No	No	Yes	Yes
**Skin Sensitization**	Yes	No	No	No	No
***T. Pyriformis* toxicity**	0.98	1.052	0.981	0.804	0.642
**Minnow toxicity**	1.138	−0.041	0.05	−2.011	0.206
**ProTox-II prediction**					
**LD_50_ (mg/kg)**	1500	2000	2000	505	40
**Toxicity Class**	IV	IV	IV	IV	II
**Immunotoxicity**	(−) (0.99)	(−) (0.91)	(−) (0.89)	(+) (0.95)	(−) (0.98)
**Mutagenicity**	(−) (0.74)	(−) (0.67)	(−) (0.66)	(−) (0.53)	(+) (0.91)
**Cytotoxicity**	(−) (0.76)	(+) (0.50)	(−) (0.50)	(+) (0.63)	(−) (0.72)
**(Tumor Suppressor) p53**	(−) (0.85)	(−) (0.80)	(−) (0.79)	(−) (0.94)	(−) (0.95)

**Table 22:** (*S*) values (Kcal·mol^−1^), interactions, and distances (Å) of donepezil (PDB ID: 4EY7) and tacrine (PDB ID: 4BDS).

Drug	(*S*) (kCal/mol)	Receptor Amino Acid	Interaction	Distance (Å)
**Donepezil**	−17.710	Phe295	H-acceptor	2.98
		Tyr341	H–arene	3.67
		Tyr337	H–arene	3.71
		Trp286	H–arene	3.87
		Trp286	arene-arene	3.88
**Tacrine**	−10.080	His438	H-donor	3.32
		His438	H–arene	4.54
		Trp82	arene-arene	3.89

**Table 23:** (*S*) values (Kcal·mol^−1^), interactions, and distances (Å) of **2**, **14**, and **16** against AChE and BChE.

Cpd. No.	(*S*) (kCal/mol)	Receptor Amino Acid	Interaction	Distance (Å)	(*S*) (kCal/mol)	Receptor Amino Acid	Interaction	Distance (Å)
	AChE				BChE			
**2**	−14.45	Ser293 Trp286 Trp286 Phe338	H-donor H-pi H-pi H-pi	3.8 4.07 4.1 4.53	−14.97	Asp70 His438 Trp82	H-donor H-pi pi-pi	4.01 4.68 3.85
**14**	−14.74	Phe295 Trp286	H-acceptor pi-pi	3.1 3.6	−18.10	Asp70 Gly78 Trp82	H-donor H-donor pi-pi	4.22 3.68 3.89
**16**	−15.09	Phe295 Trp286 Tyr341	H-acceptor H-pi pi-H	2.77 4.01 4.21	−16.41	Asp70 Gly439 Gly116 Trp82	H-donor H-acceptor H-acceptor pi-pi	4.4 3.34 3.08 3.72

## Data Availability

The data supporting the conclusions of this investigation are accessible in the publication or Supplementary File.

## Supplementary Materials

The following supporting information can be downloaded at: https://www.mdpi.com/article/10.3390/ph18081214/s1, Table S1: The sequences of primers employed in real-time RT-PCR analysis; Spectral Data for the compounds; Molecular Docking: Figure S1: Overlay between co-crystallized ligand (green) and re-docked pose (orange) of Donepezil (RMSD = 0.117 Å); Figure S2: Ligand Interactions between the complex overlay and AChE pocket (PDB ID: 4EY7); Figure S3: Overlay between co-crystallized ligand (green) and re-docked pose (orange) of Tacrine (RMSD= 0.589 Å); Figure S4: Ligand Interactions between the complex overlay and BChE pocket (PDB ID: 4BDS); Figure S5: 3D binding modes of compound **2** with AChE (PDB ID: 4EY7); Figure S6: 2D ligand interactions of compound **2** with AChE (PDB ID: 4EY7); Figure S7: 3D binding mode of compound **14** with AChE (PDB ID: 4EY7); Figure S8: 2D ligand interactions of **14** with AChE (PDB ID: 4EY7); Figure S9: 3D binding mode of compound **16** with AChE (PDB ID: 4EY7); Figure S10: 2D ligand interactions of compound **16** with AChE (PDB ID: 4EY7); Figure S11: 3D binding mode of compound **2** with BChE (PDB ID: 4BDS); Figure S12: 2D ligand interactions of compound **2** with BChE (PDB ID: 4BDS); Figure S13: 3D binding mode of compound **14** with BChE (PDB ID: 4BDS); Figure S14: 2D ligand interactions of compound **14** with BChE (PDB ID: 4BDS); Figure S15: 3D binding mode of compound **16** with BChE (PDB ID: 4BDS); Figure S16: 2D ligand interactions of compound **16** with BChE (PDB ID: 4BDS).
