# Supplementary material for: Unlocking Therapeutic Potential of Novel Thieno-Oxazepine Hybrids as Multi-Target Inhibitors of AChE/BChE and Evaluation Against Alzheimer’s Disease: In Vivo, In Vitro, Histopathological, and Docking Studies
Source: Pharmaceuticals (Basel). 2025 Aug 17;18(8):1214. doi: 10.3390/ph18081214 (PMC12389534; doi:10.3390/ph18081214)
Supplement: Supplementary file 1 [file pharmaceuticals-18-01214-s001.zip › pharmaceuticals-3796254-supplementary.pdf]

Supplementary Information

Unlocking Therapeutic Potential of Novel Thieno-Oxazepine Hybrids as Multi-Target Inhibitors of AChE/BChE and Evaluation Against Alzheimer's Disease: In Vivo, In Vitro, Histopathological, and Docking Studies

Khulood H. Oudah [1], Mazin A. A. Najm [1], Triveena M. Ramsis [2], Maha A. Ebrahim [3], Nirvana A. Gohar [4], Karema Abu-Elfotuh [5,6], Ehsan Khedre Mohamed [7], Ahmed M. E. Hamdan [8], Amira M. Hamdan [9], Reema Almotairi [10], Shaimaa R. Abdelmohsen [11], Khaled Ragab Abdelhakim [12], Abdou Mohammed Ahmed Elsharkawy [13] and Eman A. Fayed [3,*]

1 Department of Pharmacy, Mazaya University College, Nasiriyah 64001, Iraq; khuloodhayal@gmail.com (K.H.O.); mazinsajad@gmail.com (M.A.A.N.)
2 Department of Pharmaceutical Chemistry, Faculty of Pharmacy, Sinai University, Kantara Branch, Ismailia 41636, Egypt; triveena.farid@su.edu.eg
3 Department of Pharmaceutical Organic Chemistry, Faculty of Pharmacy (Girls), Al-Azhar University, Cairo 11754, Egypt; maha.ahmed@azhar.edu.eg
4 Department of Pharmaceutical Organic Chemistry, Faculty of Pharmacy, Modern University for Technology and Information (MTI), Cairo 11571, Egypt; nirvana.goher@pharm.mti.edu.eg
5 Department of Clinical Pharmacy, Faculty of Pharmacy (Girls), Al-Azhar University, Cairo 11754, Egypt; karimasoliman.pharmg@azhar.edu.eg
6 College of Pharmacy, Al-Ayen Iraqi University (AUIQ), An Nasiriyah 64004, Iraq
7 Department of Biochemistry, Egyptian Drug Authority (EDA), Formerly National Organization of Drug Control and Research (NODCAR), Giza 12654, Egypt; drehsankhedre@hotmail.com
8 Department of Pharmacy Practice, Faculty of Pharmacy, University of Tabuk, Tabuk 71491, Saudi Arabia; a_hamdan@ut.edu.sa
9 Oceanography Department, Faculty of Science, Alexandria University, Alexandria 21515, Egypt; amirahamdan@alexu.edu.eg
10 Department of Medical Laboratory Technology, Faculty of Applied Medical Sciences, University of Tabuk, Tabuk 71491, Saudi Arabia; ralmotairi@ut.edu.sa
11 Department of Anatomy and Embryology, Faculty of Medicine, Al-Azhar University, Cairo 11754, Egypt; shaimaaramadan.medg@azhar.edu.eg
12 Department of Histology, Misr University for Science and Technology, Giza 12566, Egypt; khalid.ragab@must.edu.eg
13 Anatomy Department, Faculty of Medicine, Al-Azhar University, Cairo 11754, Egypt; abdouelsharkawy@gmail.com
* Correspondence: alfayed_e@azhar.edu.eg or alfayed_e@yahoo.com

- **Chemistry**

General details

The NMR Spectrometer at the Main Chemical Warfare Laboratories, Chemical Warfare Department, Ministry of Defense, was used to perform the ^{1}H NMR Spectra at 400 MHz and the 13C NMR Spectra at 100 MHz on the Gemini 400 Mercury. All commercial solvents and reagents were purchased from generic chemical companies and used exactly as is, without any purification. The melting points were recorded without correction using the Electrothermal LA 9000 SERIS, Digital Melting Point Apparatus. Using $CDCl_3$ as the solvent, chemical changes were tracked and calculated in parts per million (ppm) in relation to TMS, which served as the internal standard. A Schimadzu GC/MS-QP5050A Spectrometer was used at the Regional Centre for Mycology and Biotechnology at Al-Azhar University to record mass spectra at a voltage of 70 eV. Analytical RP-HPLC analyses were performed on a HPLC–UV apparatus (Schimazu Lab Solutions), confirming purity of all final compounds of >95%. The apparatus was equipped with Phenomenex Kinetex C18 column (150 × 4.6 mm, 5 µm particle size). The solvent system consisted of water containing 0.1% TFA as eluent (A) and 0.1% TFA in acetonitrile as eluent (B).

The HPLC method implemented an injection volume of 50 µL through manual injection system and a flow rate of 1 mL/min. The percentage of B started at 5%, increased to 100% during 17 min, was kept at 100% for 1 min, and was flushed back to 5% in 1 min and was kept at 5% for 1 min.

- ## Biology

Experimental Design

The rats were casually assigned into eleven groups with 10 rats each as follows. Group 1: Rats were administered saline (i.p., daily for 5 weeks) and considered as a negative control. Group 2: AlCl3-induced AD by administering rats with 70 mg/kg AlCl3, i.p., daily for 5 weeks) as reported [79]. Groups 3-11: AD + either donepezil, compounds 2, 4, 11-16 (orally, 30 mg/Kg) [80]. Behavioral assessments were used to gauge the level of memory and spatial recognition impairment five weeks into the study [79-83].

ELISA Technique

Brain tissue homogenate was used to test the levels of IL-1*β* and TNF-*α* using ELISA kits. Brain-derived neurotrophic factor (BDNF) and *β*-amyloid were among the biomarkers of cognition and the degree of neurodegeneration that were estimated using ELISA kits (MyBioSource, Inc., San Diego, CA, USA). Furthermore, ELISA kits from MyBioSource, Inc., San Diego, CA, USA, were used to quantify the concentration of the *β*-secretase enzyme (BACE1). Following the manufacturer's instructions, the brain concentrations of Wnt Family Member 3A (Wnt3a) (orb555678, Biorbyt Ltd., Cambridge, UK) and Rat *β*-catenin ELISA Kit (K3383, Biovision Inc.) were assessed. Every step of the quantitative sandwich ELISA procedure was carried out according to the manufacturer's instructions [85].

Real-Time Quantitative Polymerase Chain Reaction

Using the Applied Biosystems Step One Plus device, RT-qPCR was used to measure the mRNA levels of GSK-3*β*, BAX, and Bcl-2. Total RNA was isolated using the Qiagen tissue extraction kit (Qiagen, Germantown, MD, USA) in accordance with the manufacturer's instructions. The Maxima SYBR Green qPCR kit (Fermentas, Hanover, MD, USA) was used to amplify the extracted mRNA after it had been reverse-

transcribed using a Sense Rapid cDNA Synthesis kit (CAT No. BIO-65053) (**Table S1**). The ABI Prism 7500 sequence detection system (Applied Biosystems, Foster City, CA, USA) was used to quantify the mRNA levels. In order to determine the relative expression of the target genes, the findings were normalized to *β*-actin expression using the 2−ΔΔCT technique [82].

Table S1: The sequences of primers employed in real-time RT-PCR analysis.

Target gene	Primer sequence	Accession Number
**BAX**	F: 5'-CACGTCTGCGGGGAGTCA-3',<br>R: 5’-TAGGAAAGGAGGCCATCCCA-3	NM_017059
**Bcl-2**	F: 5'-CATCTCATGCCAAGGGGGAA-3',<br>R: 5'-TATCCCACTCGTAGCCCCTC-3'	NM_016993
**GSK-3β**	F: 5'-AGCCTATATCCATTCCTTGG-3',<br>R: 5'-CCTCGGACCAGCTGCTTT-3'	NM_032080
**HO-1**	F: 5′ -CACCAGCCACACAGCACTAC-3′,<br>R: 5′ -CACCCACCCCTCAAAAGACA-3′	NM_012580
**Nrf2**	F: 5′-CTCTCTGGAGACGGCCATGACT-3′,<br>R:5′-CTGGGCTGGGGACAGTGGTAGT-3′	NM_031789
**NFKβ**	F: 5′-TTCCTCAGCCATGGTACCTC-3′,<br>R: 5′-CCCCAAGTCTTCATCAGCAT-3′	NM_199267
**TLR4**	F: 5′ -TCAGCTTTGGTCAGTTGGCT-3′,<br>R: 5′ -GTCCTTGACCCACTGCAAGA-3′	NM_019178
**CASP-1**	F: 5′-GAACAAAGAAGGTGGCGCAT-3′,<br>R: 5′ -GAGGTCAACATCAGCTCCGA-3′	NM_012762
**NLRP3**	F: 5′ -TGCATGCCGTATCTGGTTGT-3′,<br>R: 5′ -ACCTCTTGCGAGGGTCTTTG-3′	NM_001191642
***β*-actin**	F: 5'-CCGTAAAGACCTCTATGCCA-3',<br>R: 5'-AAGAAAGGGTGTAAAACGCA-3'	NM_031144

Spectral Data

Compound 4

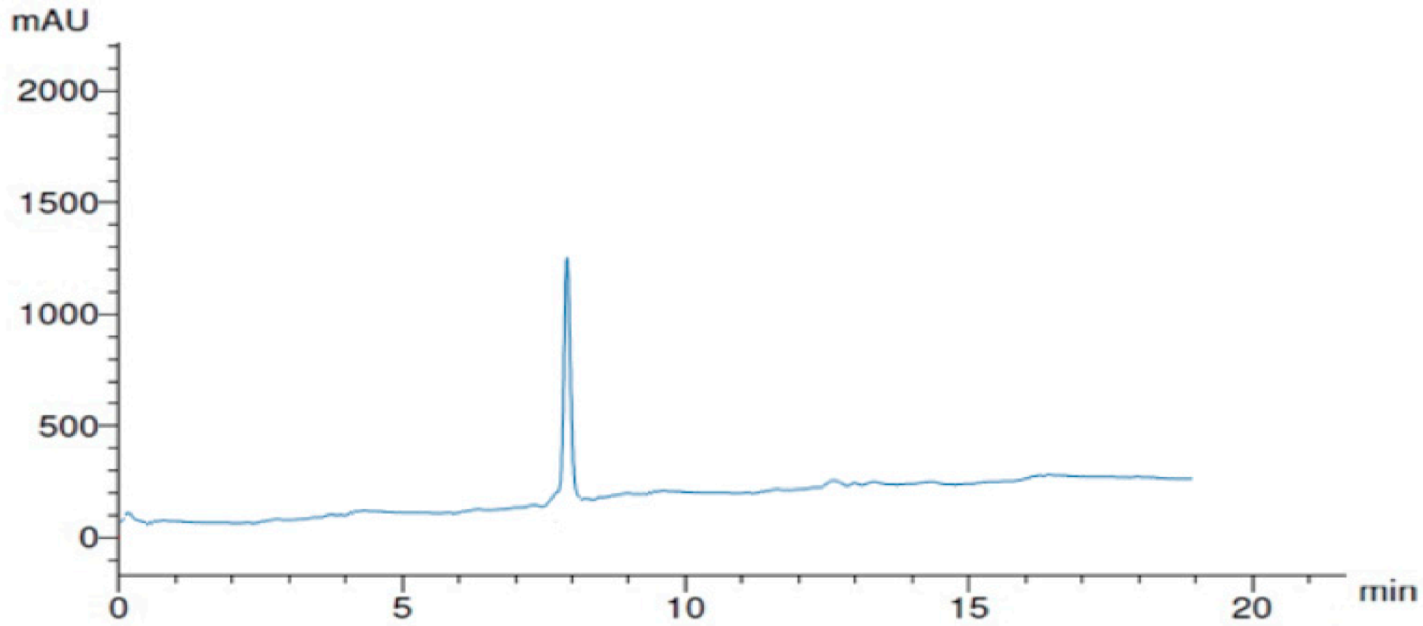

Ret. Time	Area (mAu)	Area %
7.9	95.027	100.0

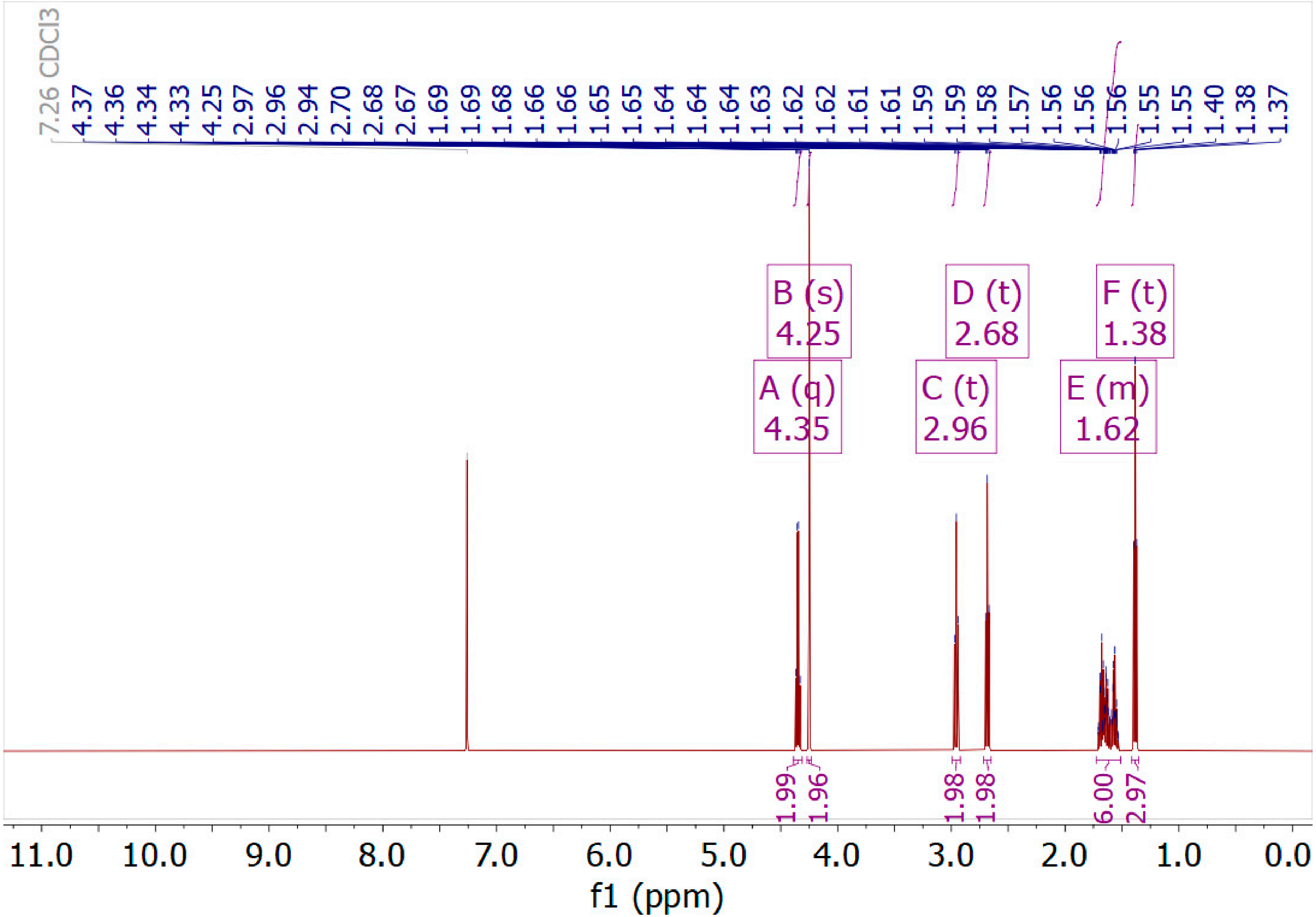
7.26 CDCl3
4.37
4.36
4.34
4.33
4.25
2.97
2.96
2.94
2.70
2.68
2.67
1.69
1.69
1.68
1.66
1.66
1.65
1.65
1.64
1.64
1.64
1.63
1.62
1.62
1.61
1.61
1.59
1.59
1.58
1.57
1.56
1.56
1.56
1.55
1.55
1.40
1.38
1.37
B (s) 4.25
D (t) 2.68
F (t) 1.38
A (q) 4.35
C (t) 2.96
E (m) 1.62
1.99
1.96
1.98
1.98
6.00
2.97
11.0
10.0
9.0
8.0
7.0
6.0
5.0
4.0
3.0
2.0
1.0
0.0
f1 (ppm)

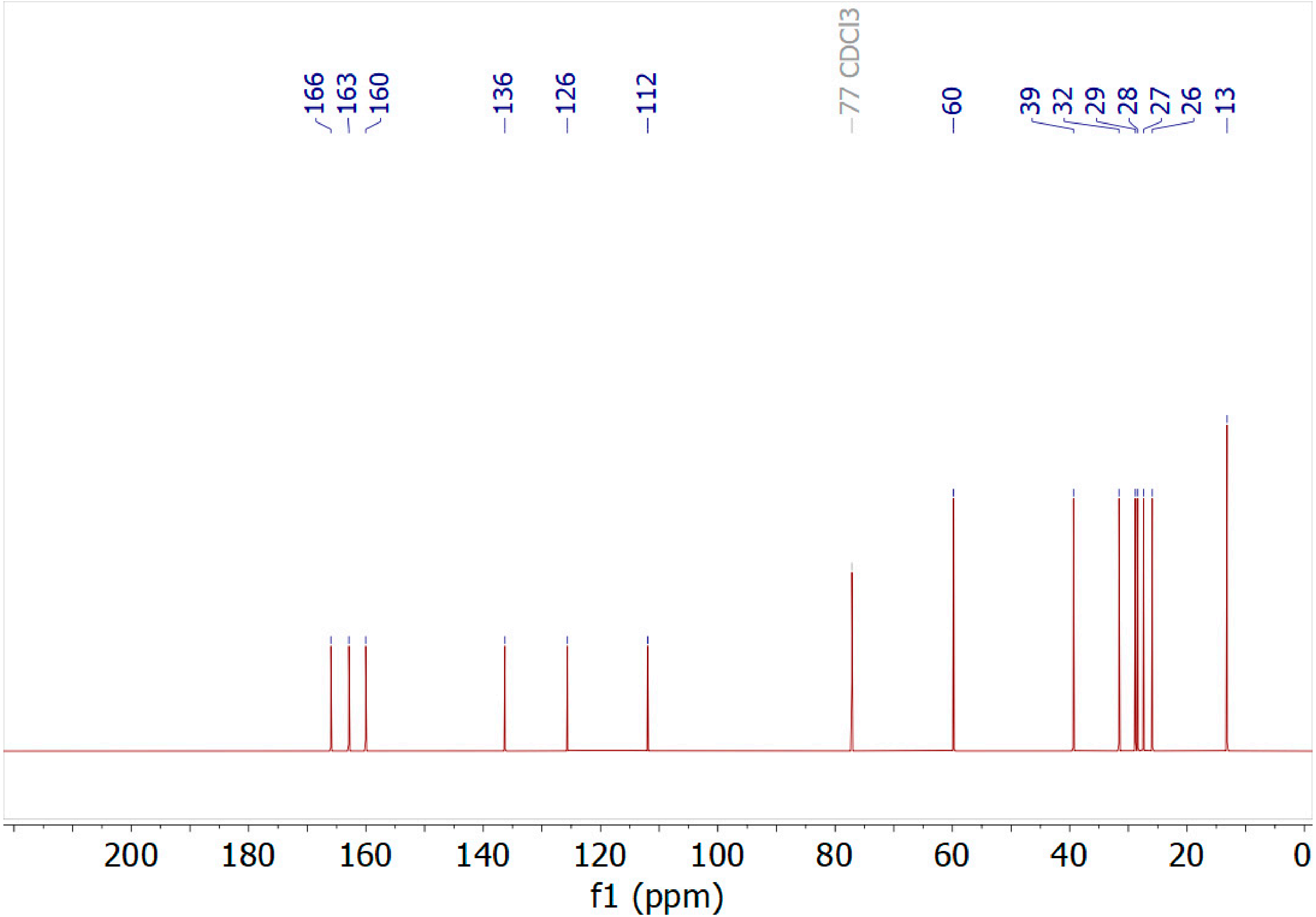
166
163
160
136
126
112
77 CDCl3
60
39
32
29
28
27
26
13
200
180
160
140
120
100
80
60
40
20
0
f1 (ppm)

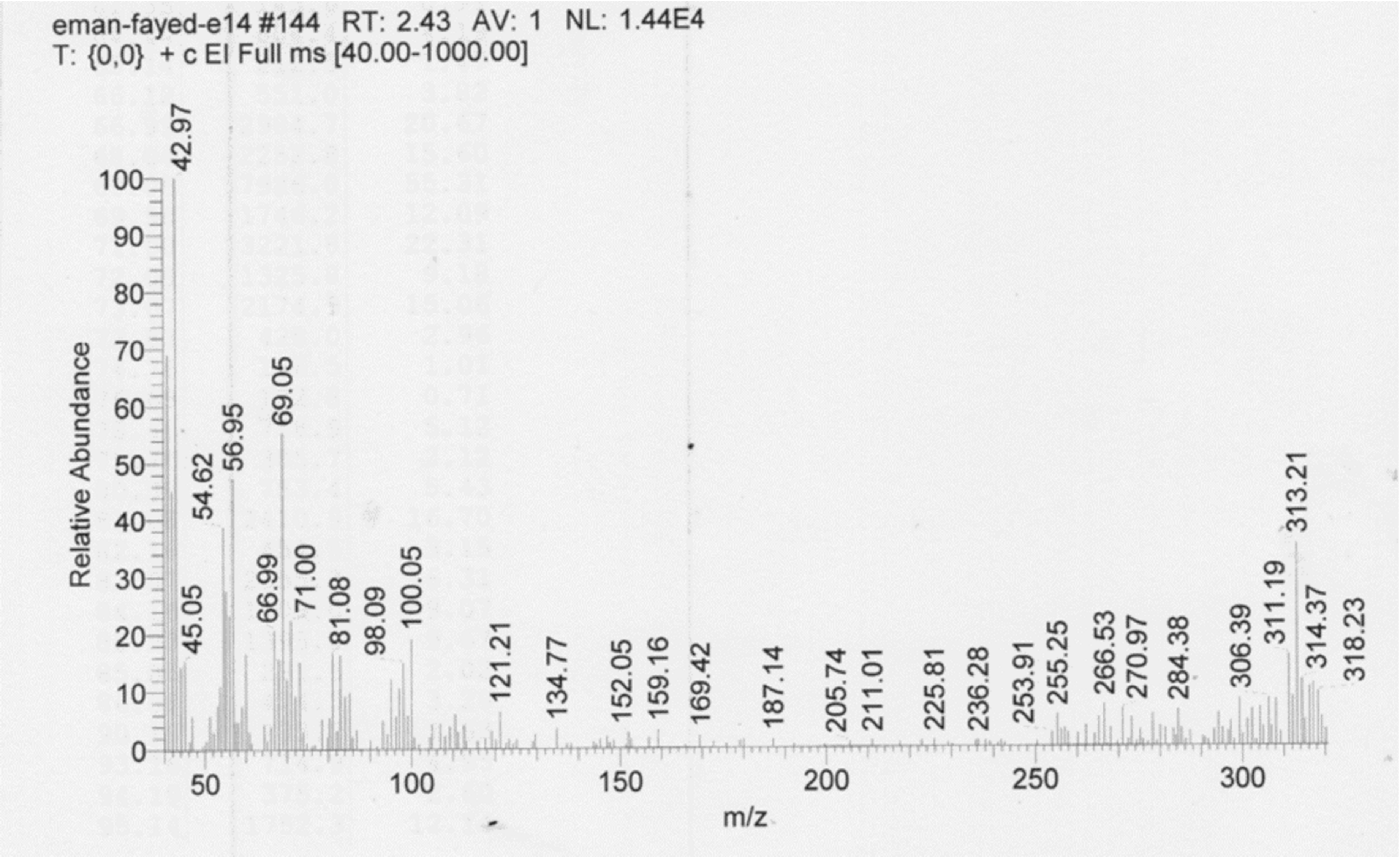
eman-fayed-e14 #144 RT: 2.43 AV: 1 NL: 1.44E4
T: {0,0} + c EI Full ms [40.00-1000.00]
Relative Abundance
m/z
42.97
45.05
54.62
56.95
66.99
69.05
71.00
81.08
98.09
100.05
121.21
134.77
152.05
159.16
169.42
187.14
205.74
211.01
225.81
236.28
253.91
255.25
266.53
270.97
284.38
306.39
311.19
313.21
314.37
318.23

Compound 11

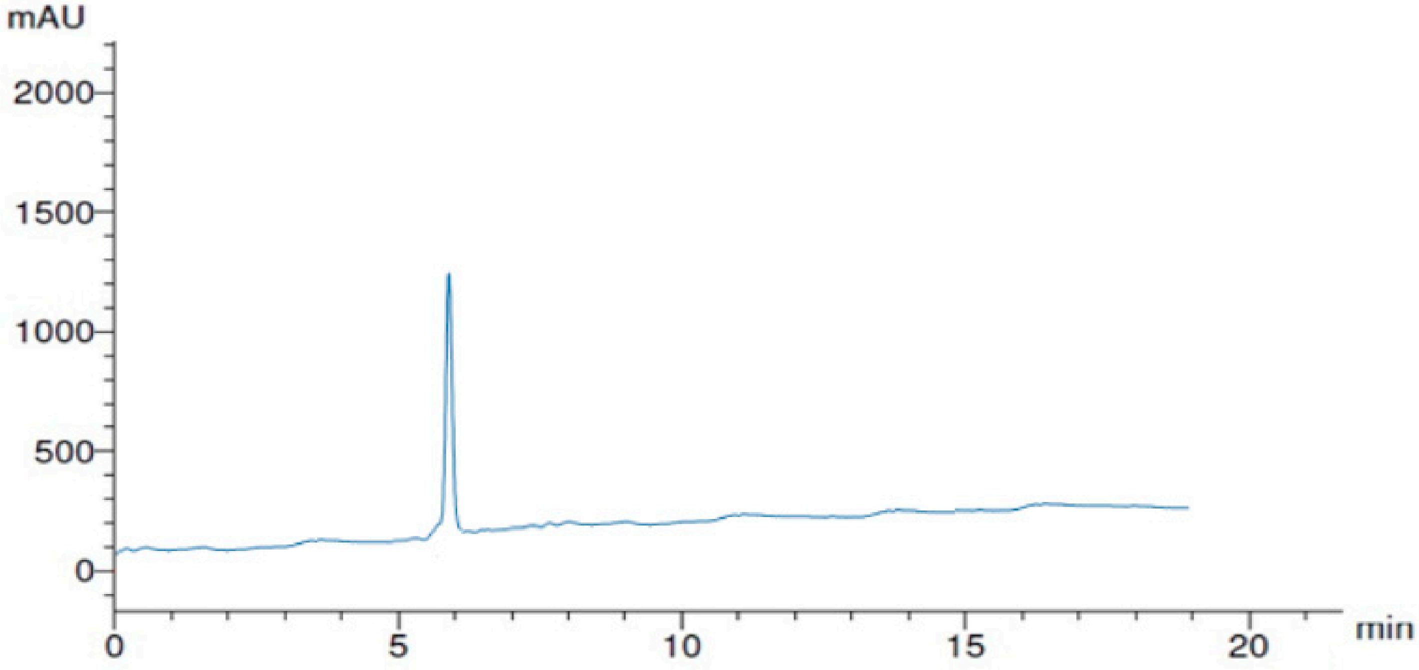

Ret. Time	Area (mAu)	Area %
6. 0	88. 074	100. 0

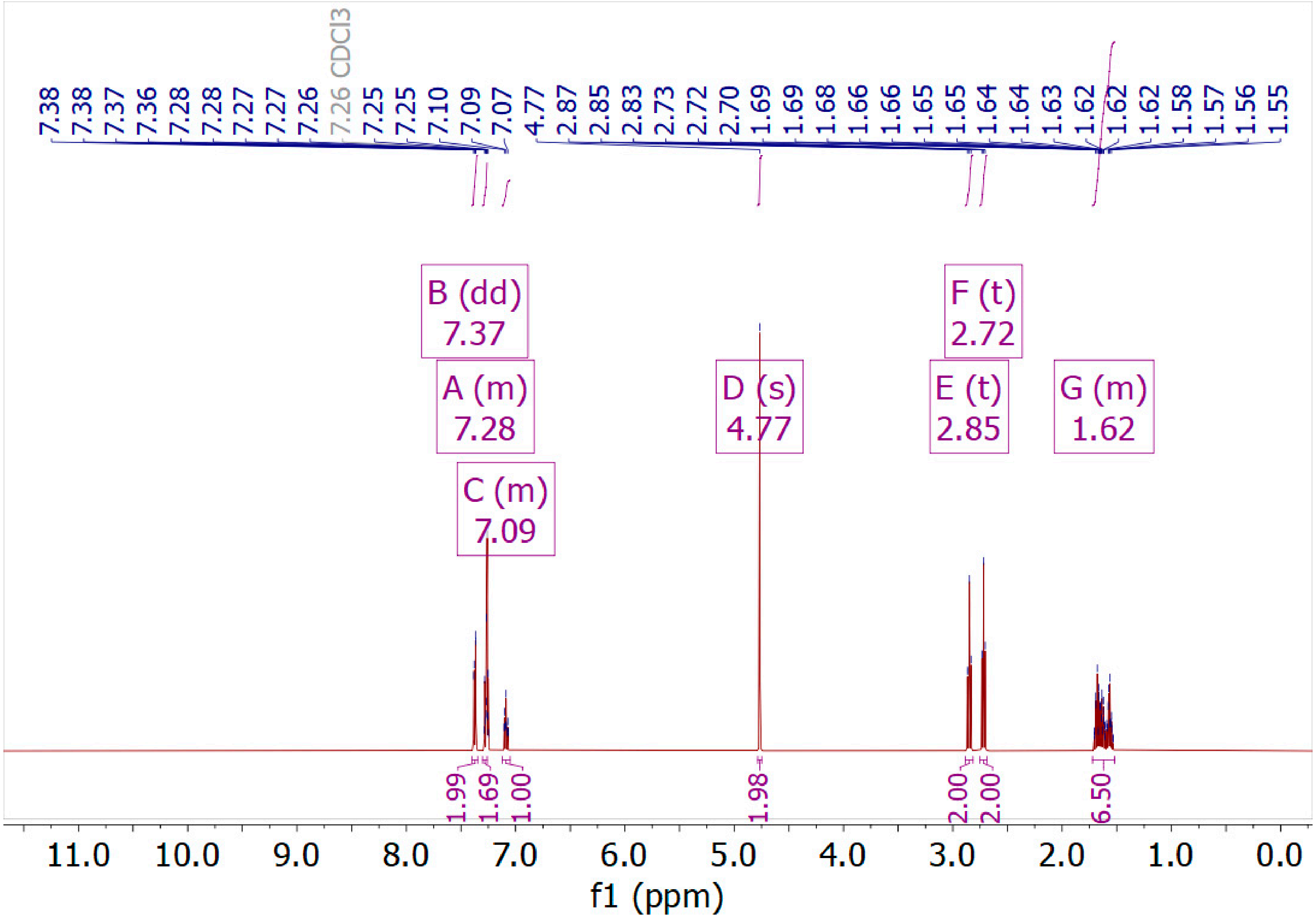
7.38
7.38
7.37
7.36
7.28
7.28
7.27
7.27
7.26
7.26 CDCl3
7.25
7.25
7.10
7.09
7.07
4.77
2.87
2.85
2.83
2.73
2.72
2.70
1.69
1.69
1.68
1.66
1.66
1.65
1.65
1.64
1.64
1.63
1.62
1.62
1.62
1.58
1.57
1.56
1.55
B (dd)
7.37
A (m)
7.28
C (m)
7.09
D (s)
4.77
F (t)
2.72
E (t)
2.85
G (m)
1.62
1.99
1.69
1.00
1.98
2.00
2.00
6.50
11.0
10.0
9.0
8.0
7.0
6.0
5.0
4.0
3.0
2.0
1.0
0.0
f1 (ppm)

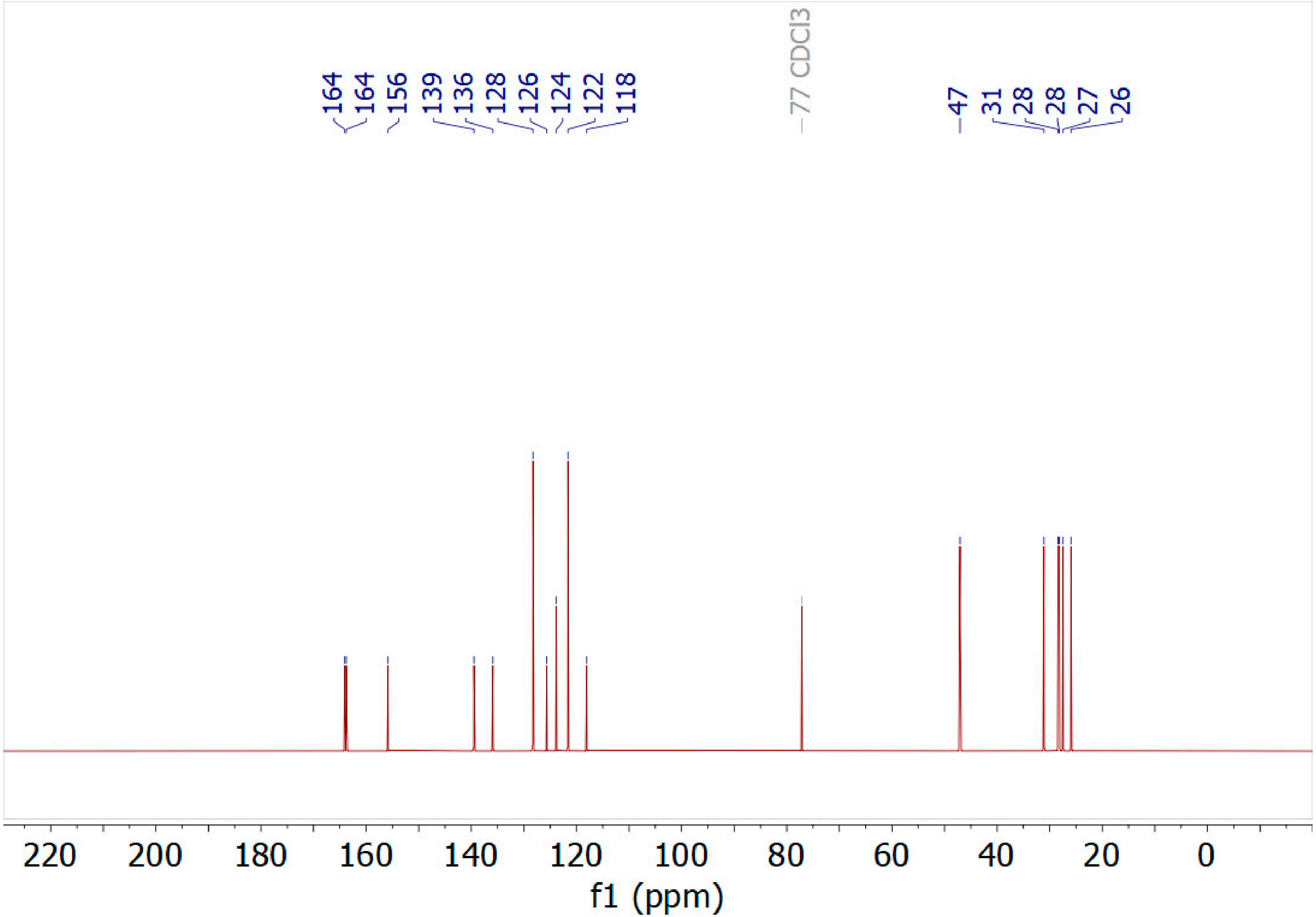
164
164
156
139
136
128
126
124
122
118
77 CDCl3
47
31
28
28
27
26
220
200
180
160
140
120
100
80
60
40
20
0
f1 (ppm)

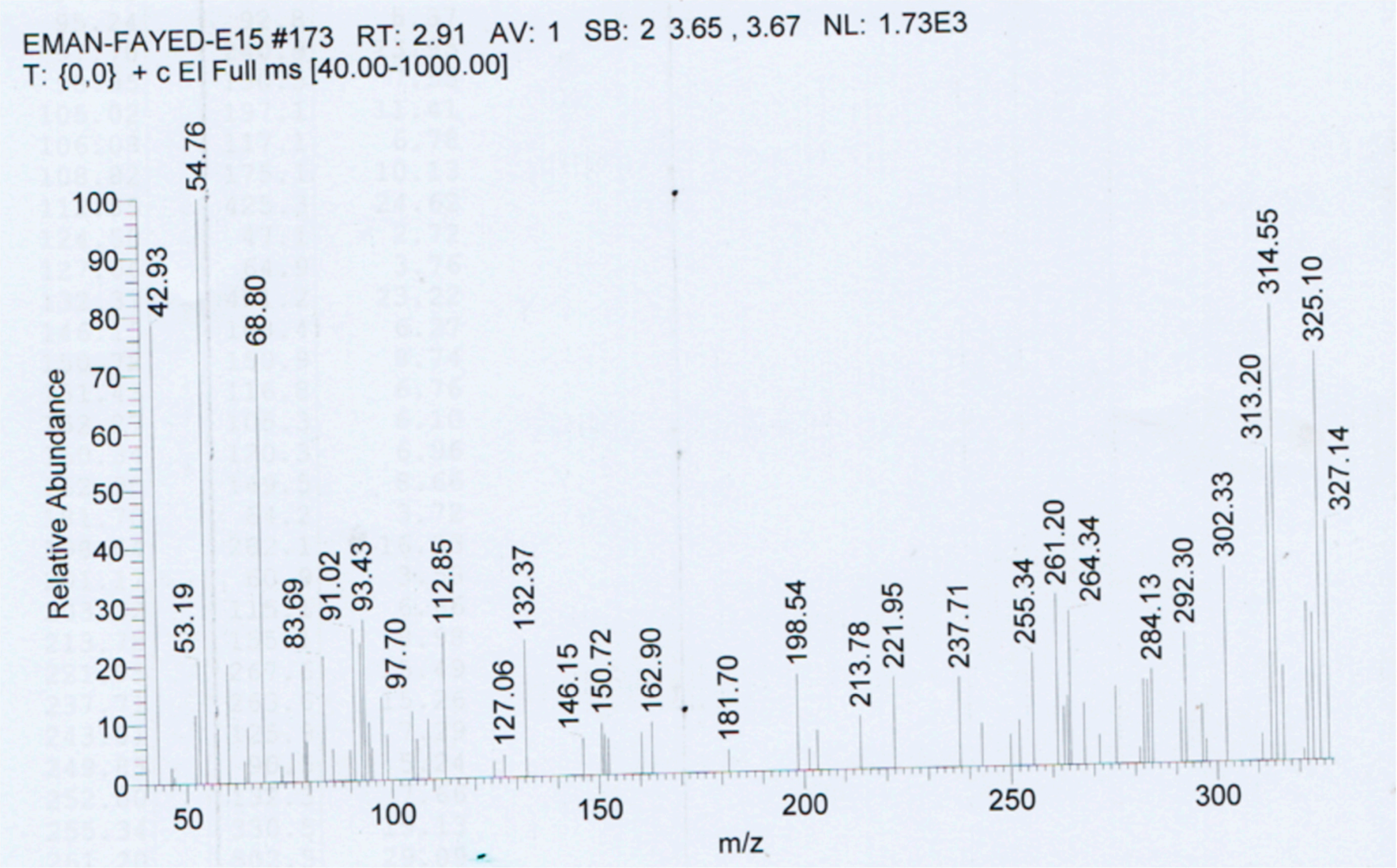
EMAN-FAYED-E15 #173 RT: 2.91 AV: 1 SB: 2 3.65 , 3.67 NL: 1.73E3
T: {0,0} + c EI Full ms [40.00-1000.00]
Relative Abundance
m/z
42.93
53.19
54.76
68.80
83.69
91.02
93.43
97.70
112.85
127.06
132.37
146.15
150.72
162.90
181.70
198.54
213.78
221.95
237.71
255.34
261.20
264.34
284.13
292.30
302.33
313.20
314.55
325.10
327.14

Compound 12

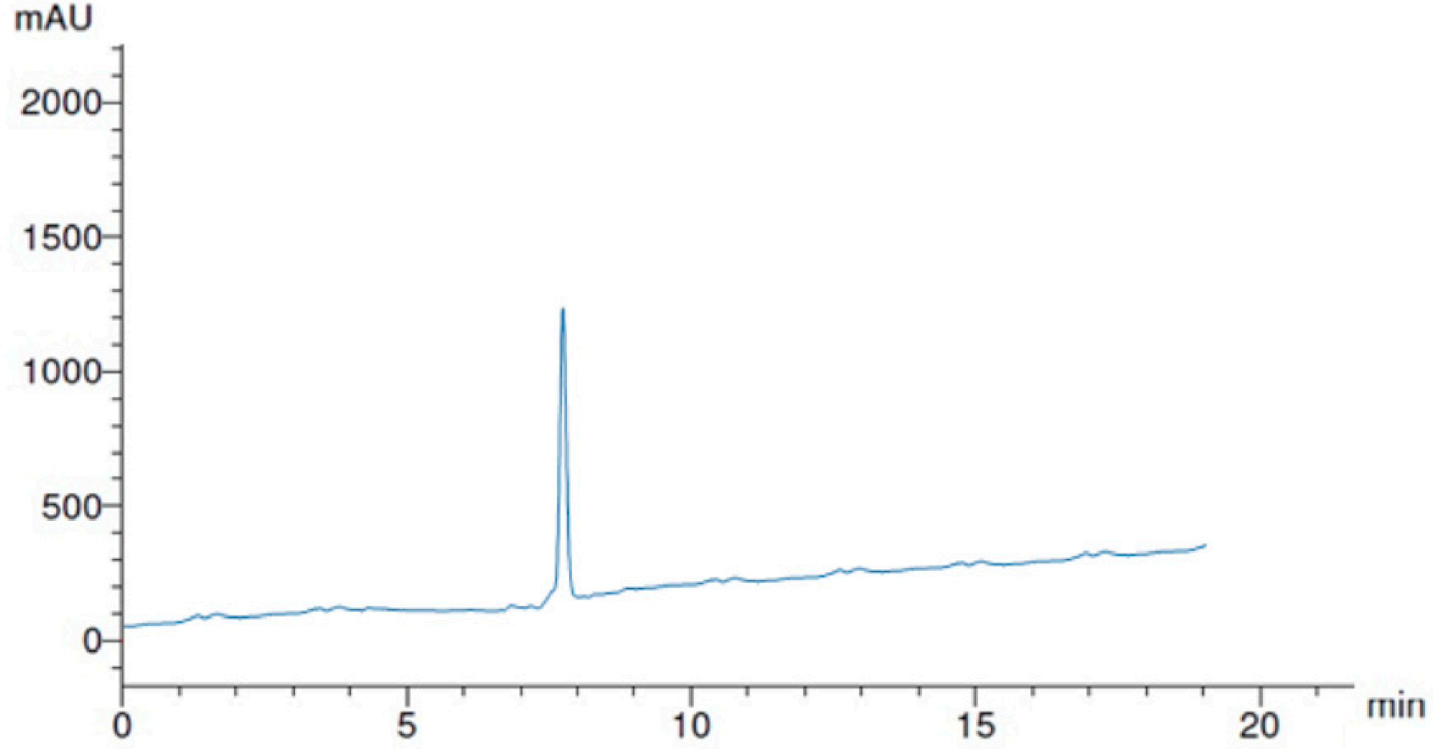

Ret. Time	Area (mAu)	Area %
7.0	88.102	100 %

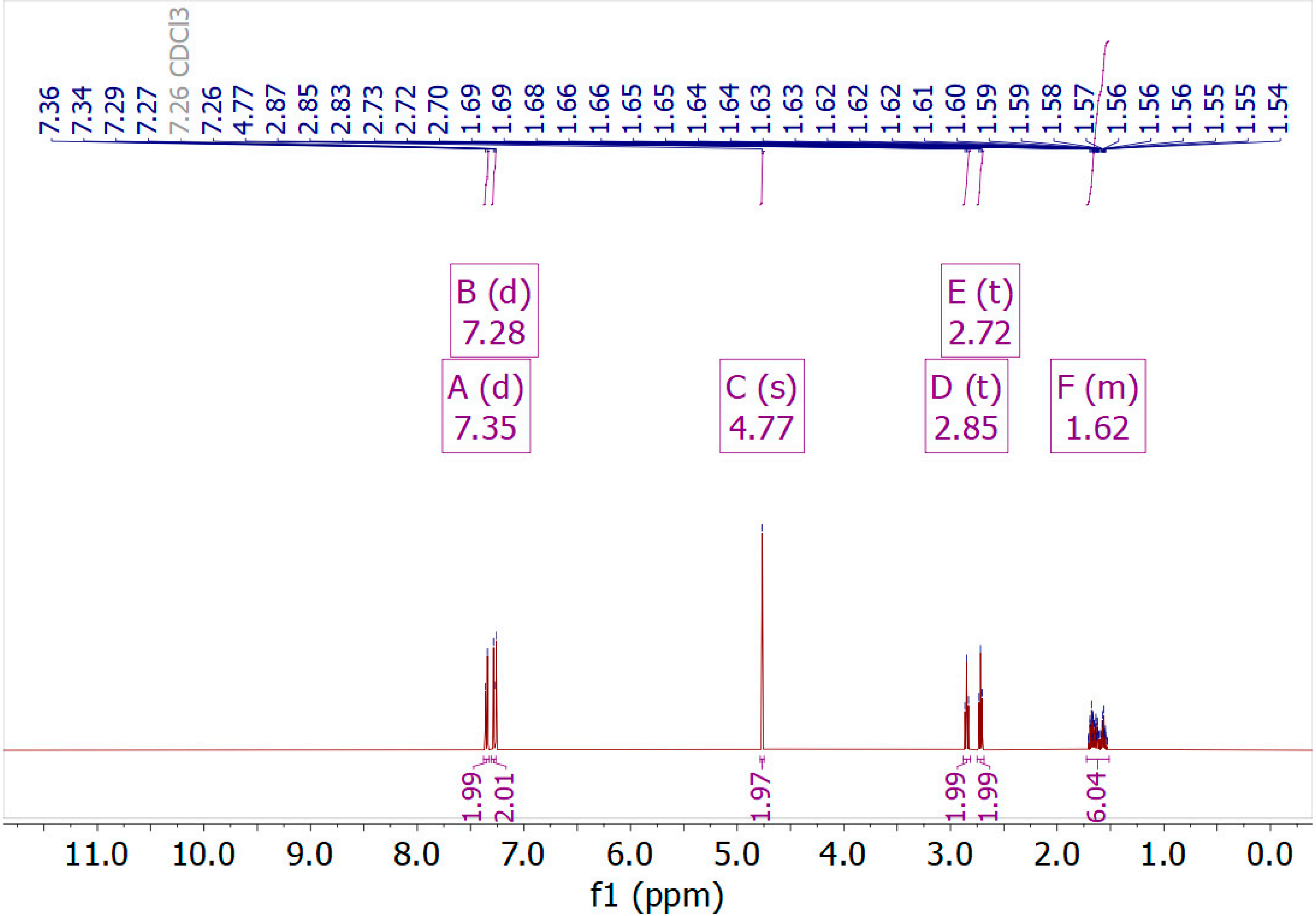
7.36
7.34
7.29
7.27
7.26 CDCl3
7.26
4.77
2.87
2.85
2.83
2.73
2.72
2.70
1.69
1.69
1.68
1.66
1.66
1.65
1.65
1.64
1.64
1.63
1.63
1.62
1.62
1.62
1.61
1.60
1.59
1.59
1.58
1.57
1.56
1.56
1.56
1.55
1.55
1.54
B (d) 7.28
E (t) 2.72
A (d) 7.35
C (s) 4.77
D (t) 2.85
F (m) 1.62
1.99
2.01
1.97
1.99
1.99
6.04
11.0
10.0
9.0
8.0
7.0
6.0
5.0
4.0
3.0
2.0
1.0
0.0
f1 (ppm)

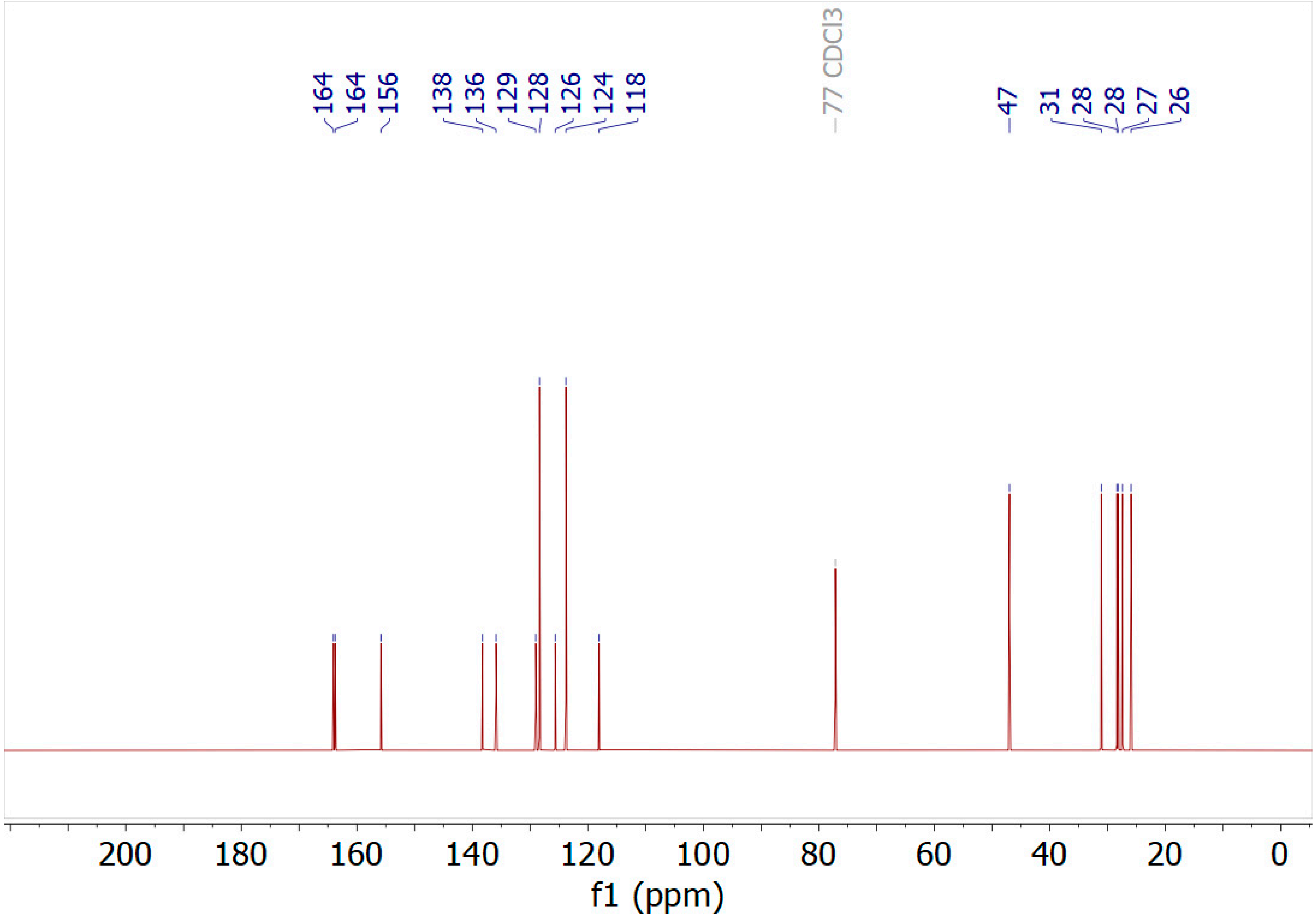

164
164
156
138
136
129
128
126
124
118
77 CDCl3
47
31
28
28
27
26
200
180
160
140
120
100
80
60
40
20
0
f1 (ppm)

Compound 13

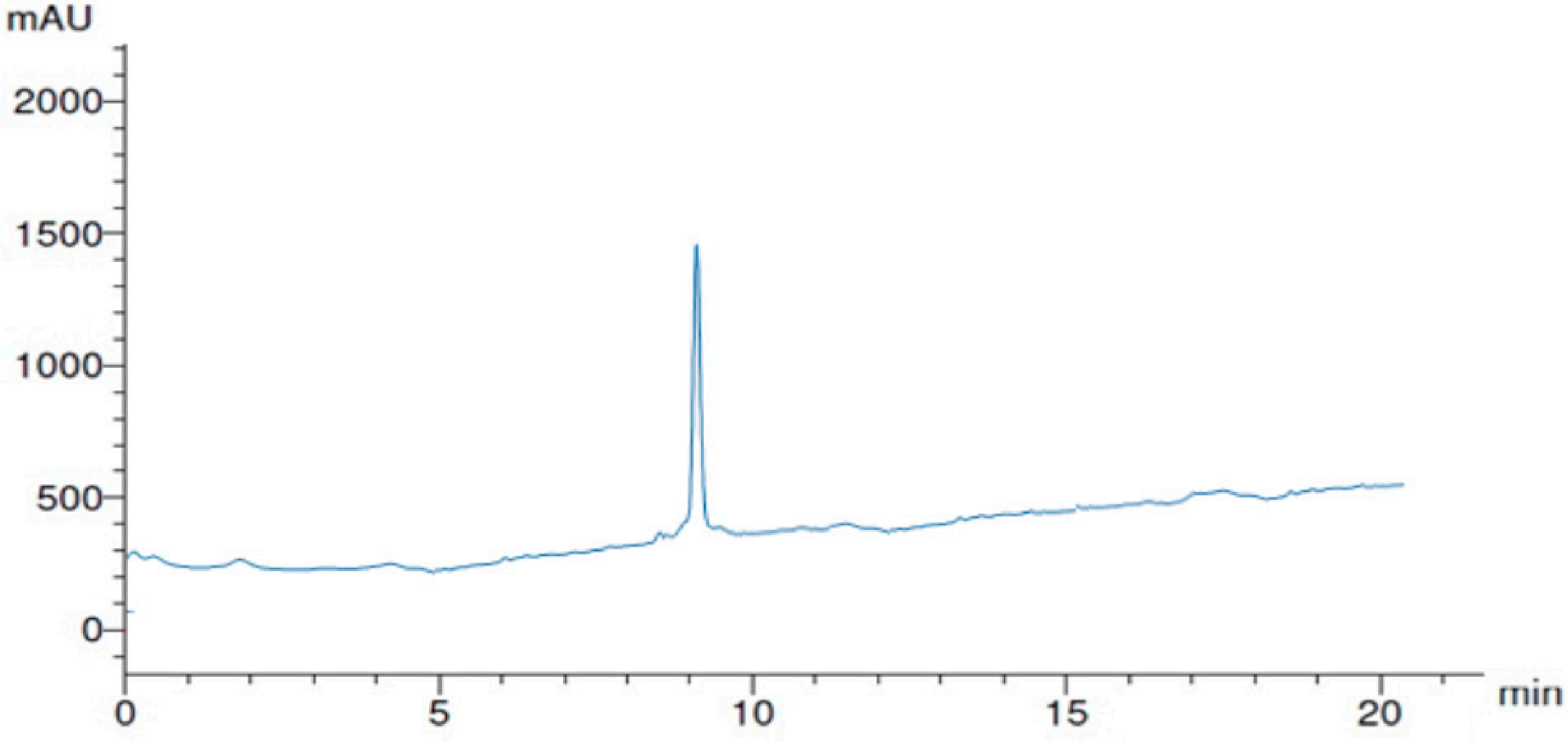

Ret. Time	Area (mAu)	Area %
9.0	77.632	100 %

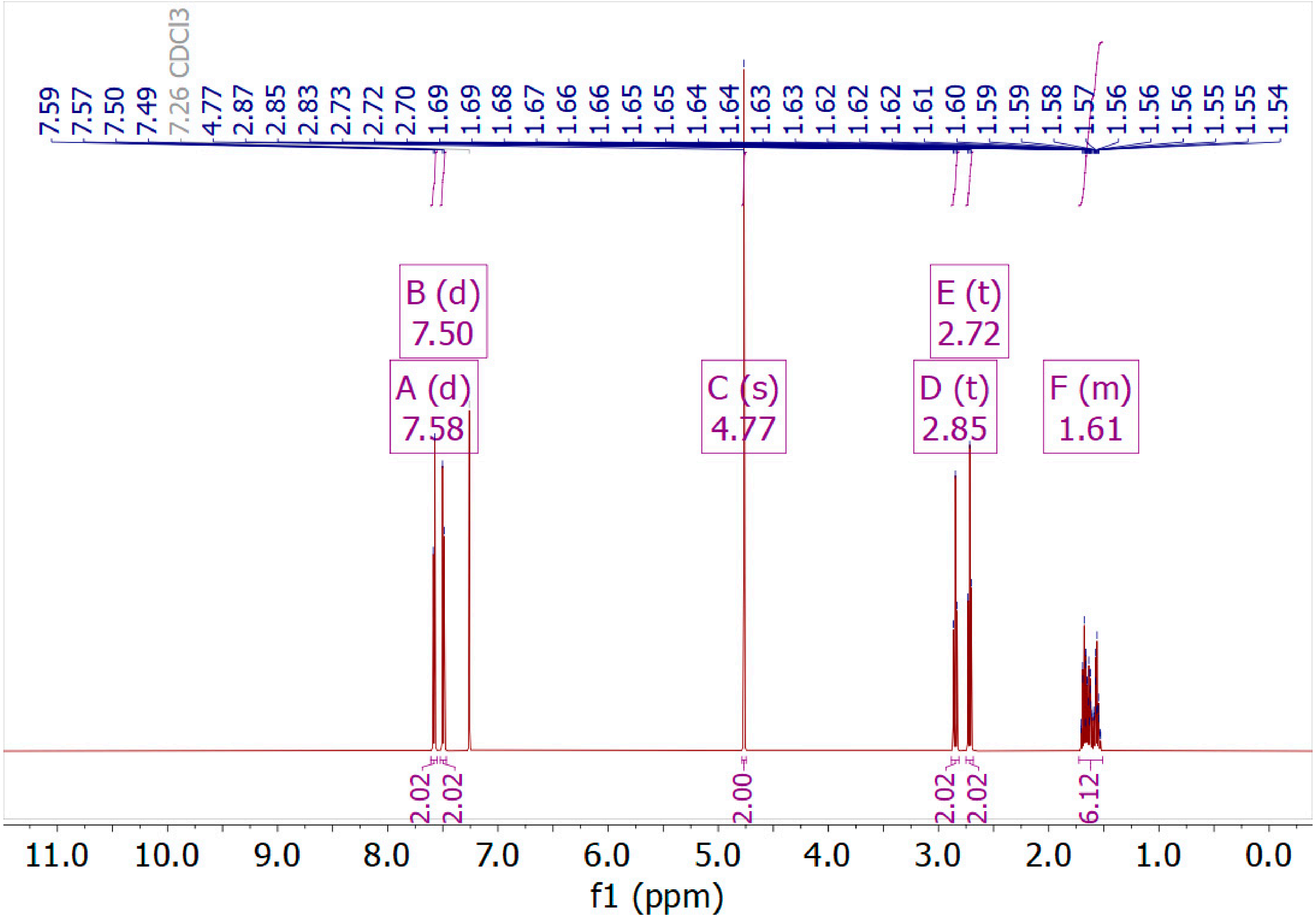
7.59 7.57 7.50 7.49 7.26 CDCl3 4.77 2.87 2.85 2.83 2.73 2.72 2.70 1.69 1.69 1.68 1.67 1.66 1.66 1.65 1.65 1.64 1.64 1.63 1.63 1.62 1.62 1.62 1.61 1.60 1.59 1.59 1.58 1.57 1.56 1.56 1.56 1.55 1.55 1.54
B (d) 7.50
A (d) 7.58
C (s) 4.77
E (t) 2.72
D (t) 2.85
F (m) 1.61
2.02 2.02 2.00 2.02 2.02 6.12
11.0 10.0 9.0 8.0 7.0 6.0 5.0 4.0 3.0 2.0 1.0 0.0
f1 (ppm)

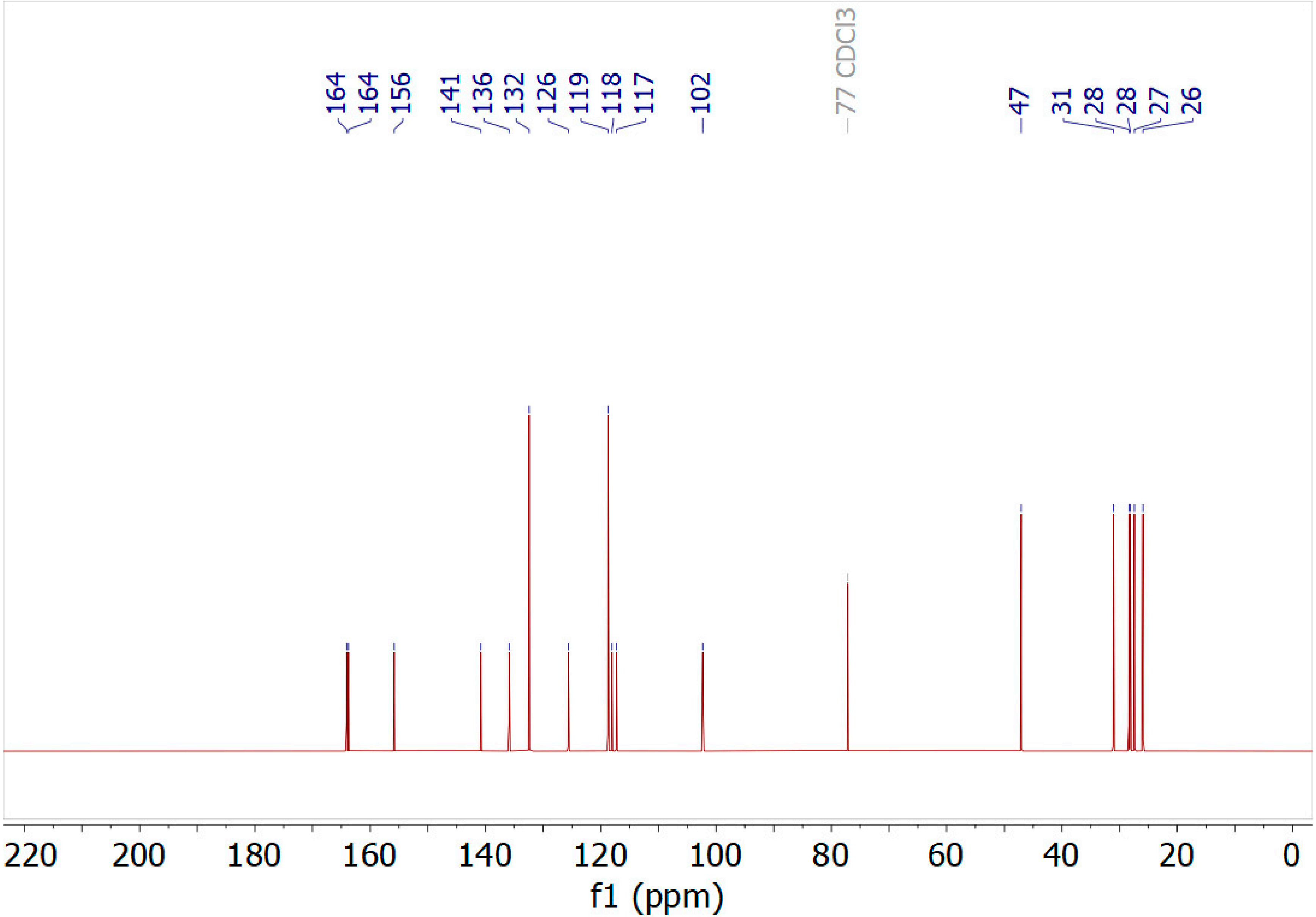
164
164
156
141
136
132
126
119
118
117
102
77 CDCl3
47
31
28
28
27
26
220
200
180
160
140
120
100
80
60
40
20
0
f1 (ppm)

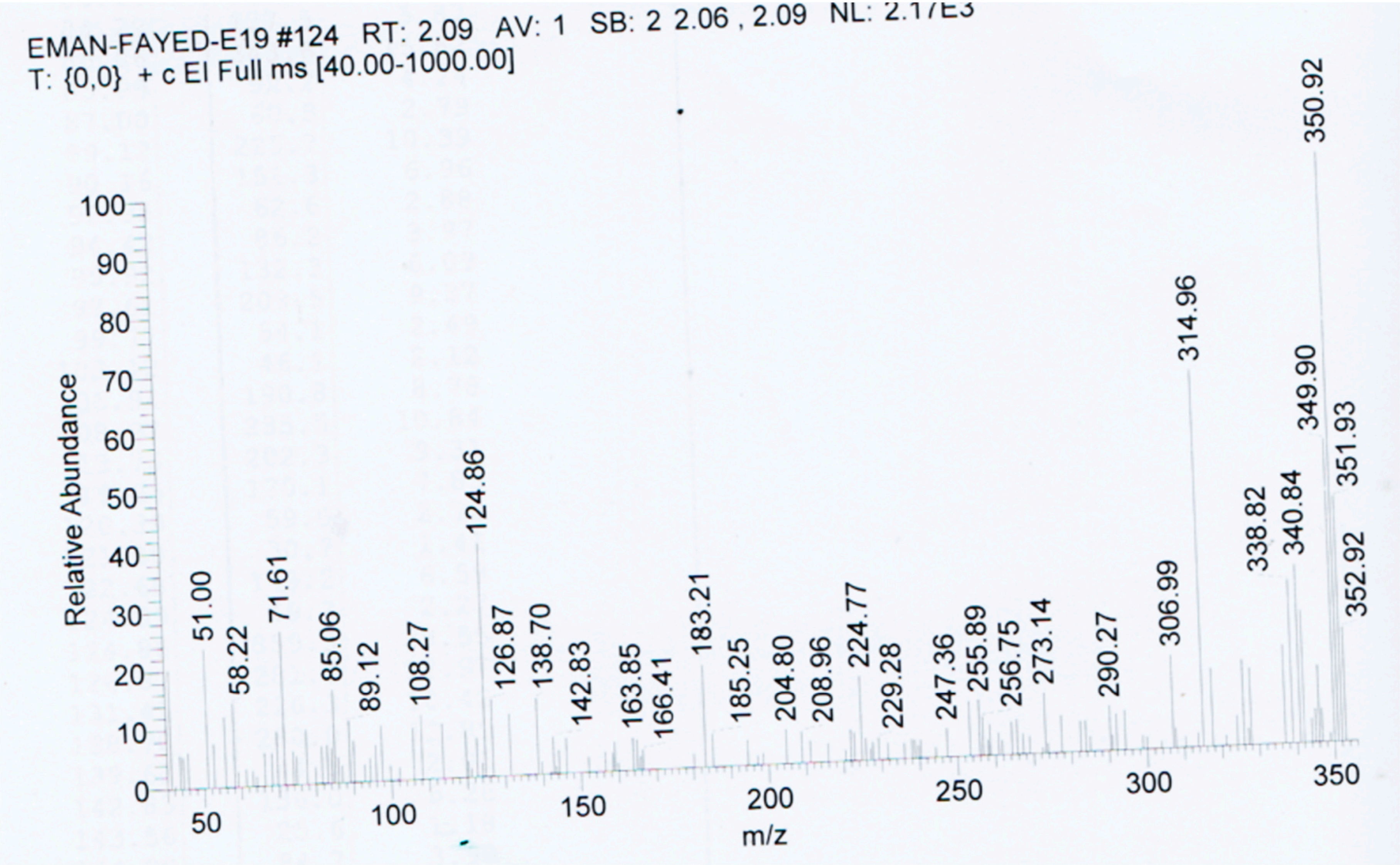
EMAN-FAYED-E19 #124 RT: 2.09 AV: 1 SB: 2 2.06 , 2.09 NL: 2.17E3
T: {0,0} + c EI Full ms [40.00-1000.00]
Relative Abundance
m/z
350.92
314.96
349.90
351.93
340.84
338.82
352.92
306.99
290.27
273.14
256.75
255.89
247.36
229.28
224.77
208.96
204.80
185.25
183.21
166.41
163.85
142.83
138.70
126.87
124.86
108.27
89.12
85.06
71.61
58.22
51.00

Compound 14

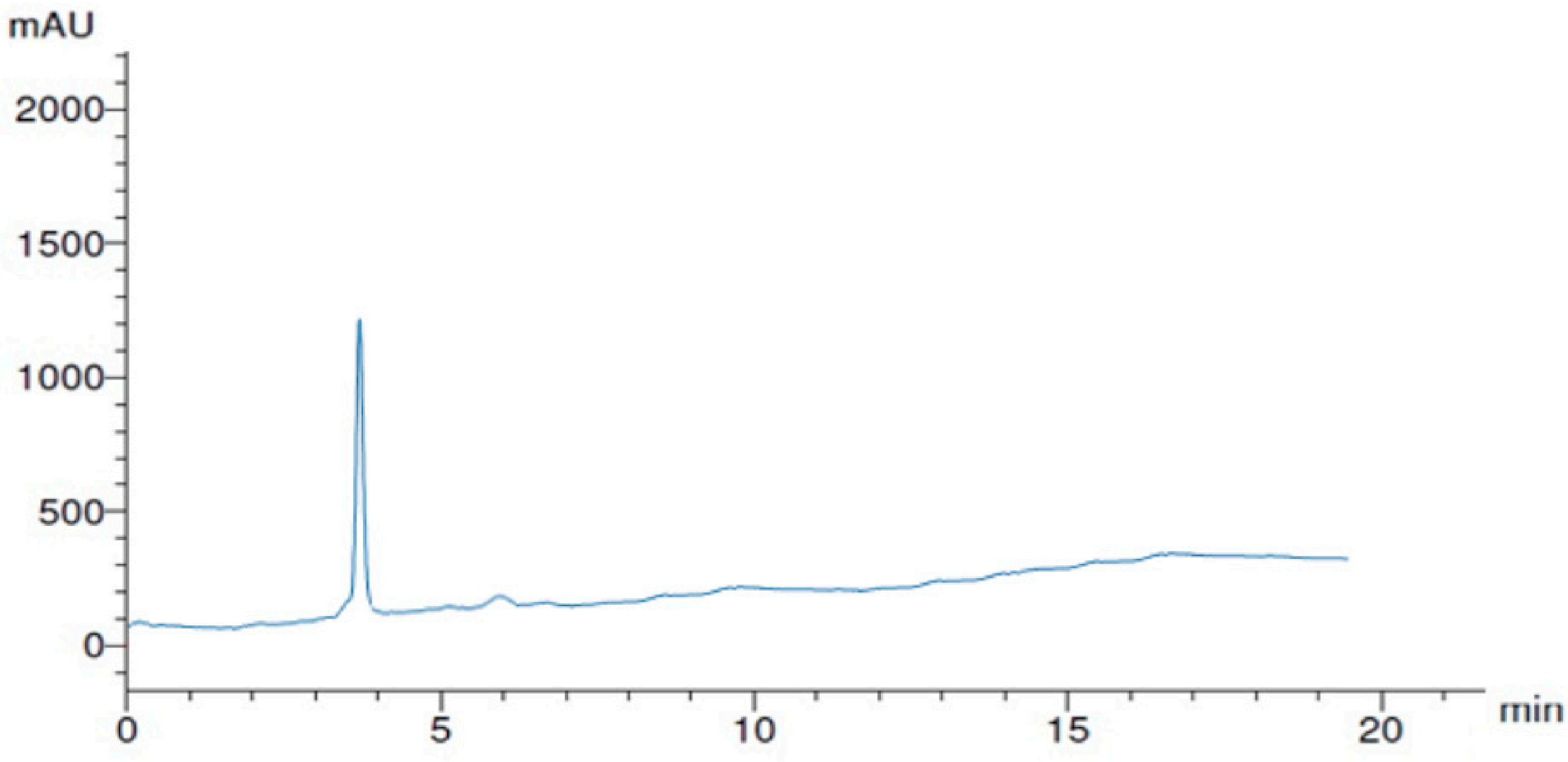

Ret. Time	Area (mAu)	Area %
4.0	70.114	100.0

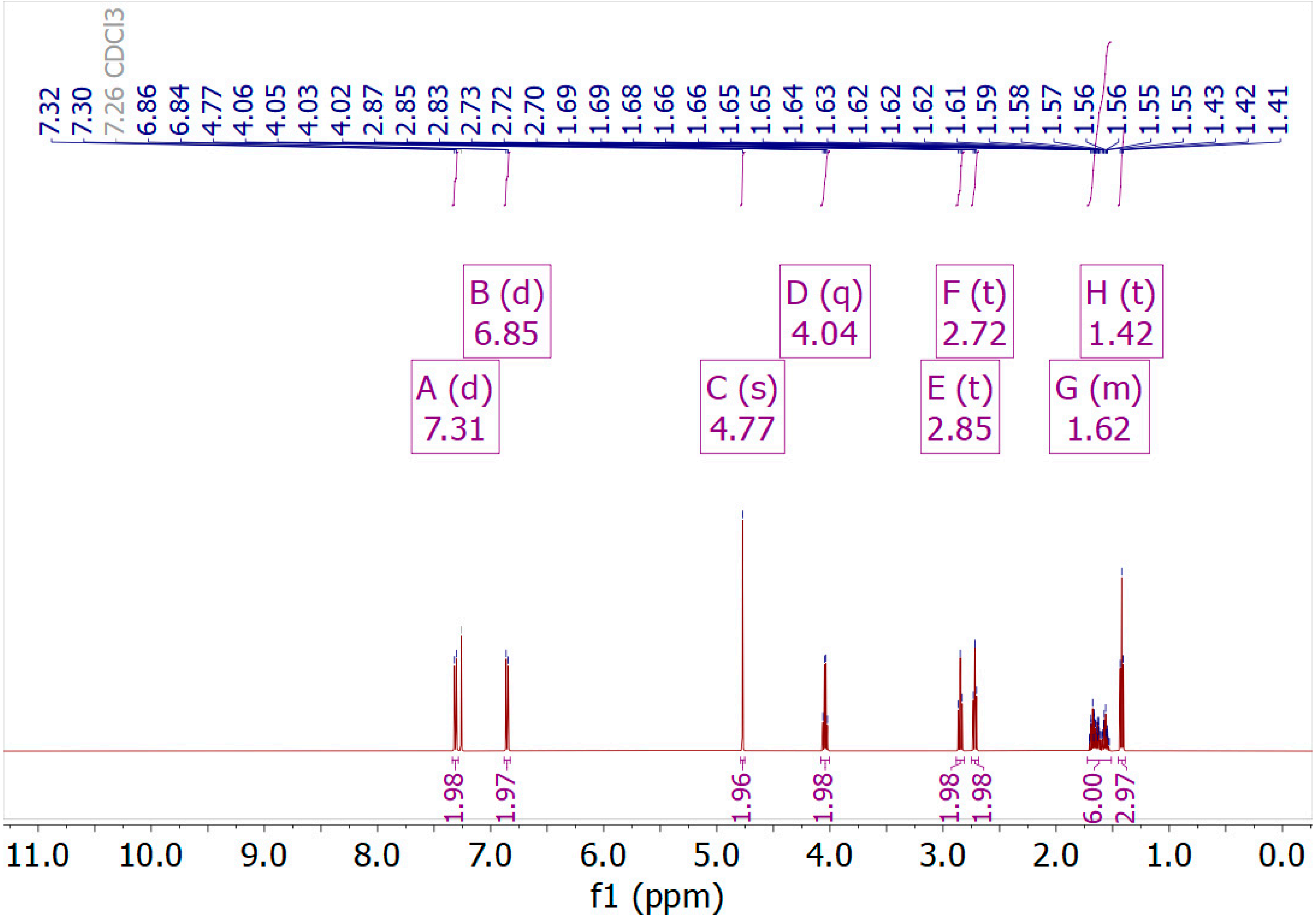

7.32
7.30
7.26 CDCl3
6.86
6.84
4.77
4.06
4.05
4.03
4.02
2.87
2.85
2.83
2.73
2.72
2.70
1.69
1.69
1.68
1.66
1.66
1.65
1.65
1.64
1.63
1.62
1.62
1.62
1.61
1.59
1.58
1.57
1.56
1.56
1.55
1.55
1.43
1.42
1.41
B (d) 6.85
D (q) 4.04
F (t) 2.72
H (t) 1.42
A (d) 7.31
C (s) 4.77
E (t) 2.85
G (m) 1.62
1.98
1.97
1.96
1.98
1.98
1.98
6.00
2.97
11.0
10.0
9.0
8.0
7.0
6.0
5.0
4.0
3.0
2.0
1.0
0.0
f1 (ppm)

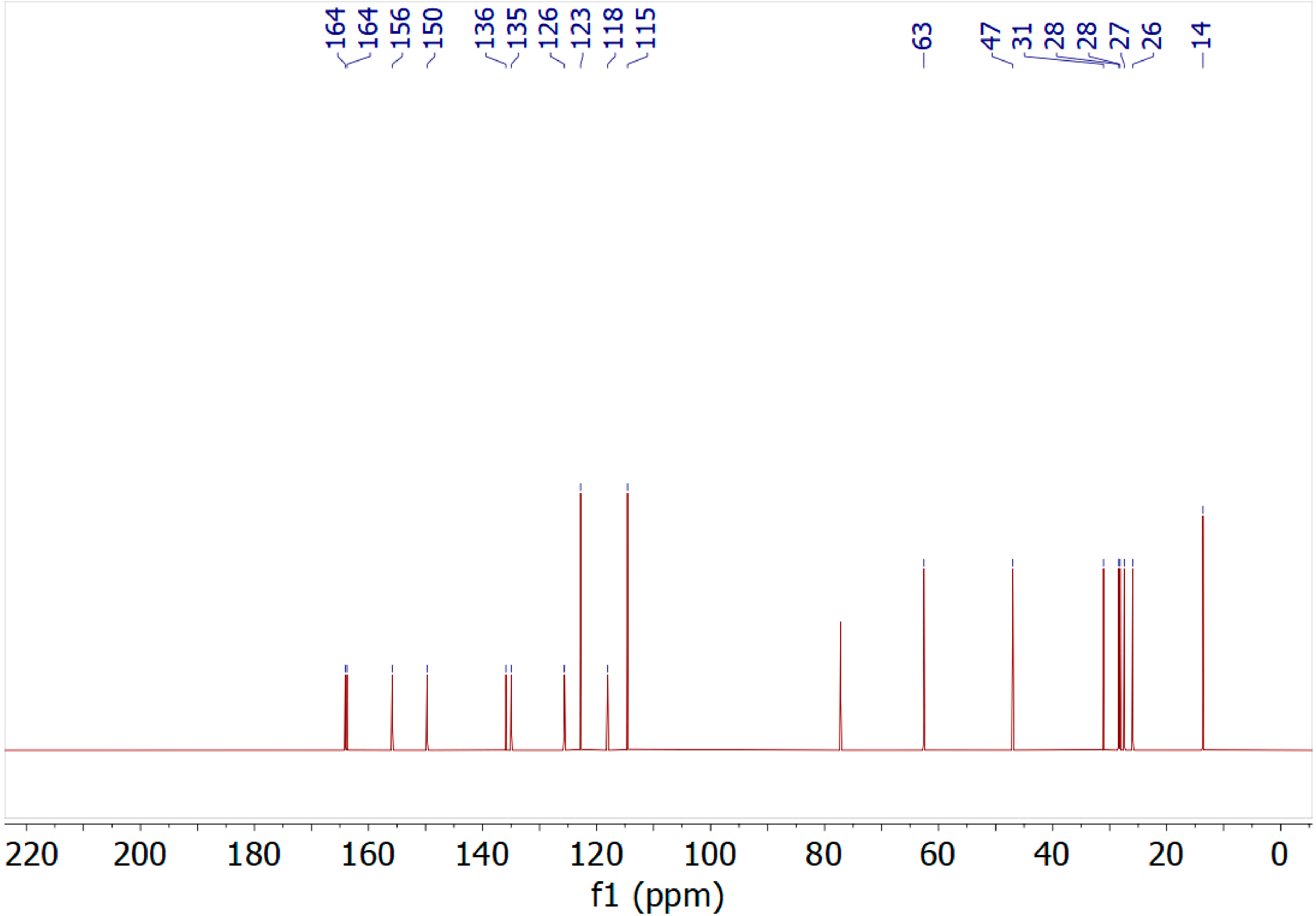
164
164
156
150
136
135
126
123
118
115
63
47
31
28
28
27
26
14
220
200
180
160
140
120
100
80
60
40
20
0
f1 (ppm)

Compound 15

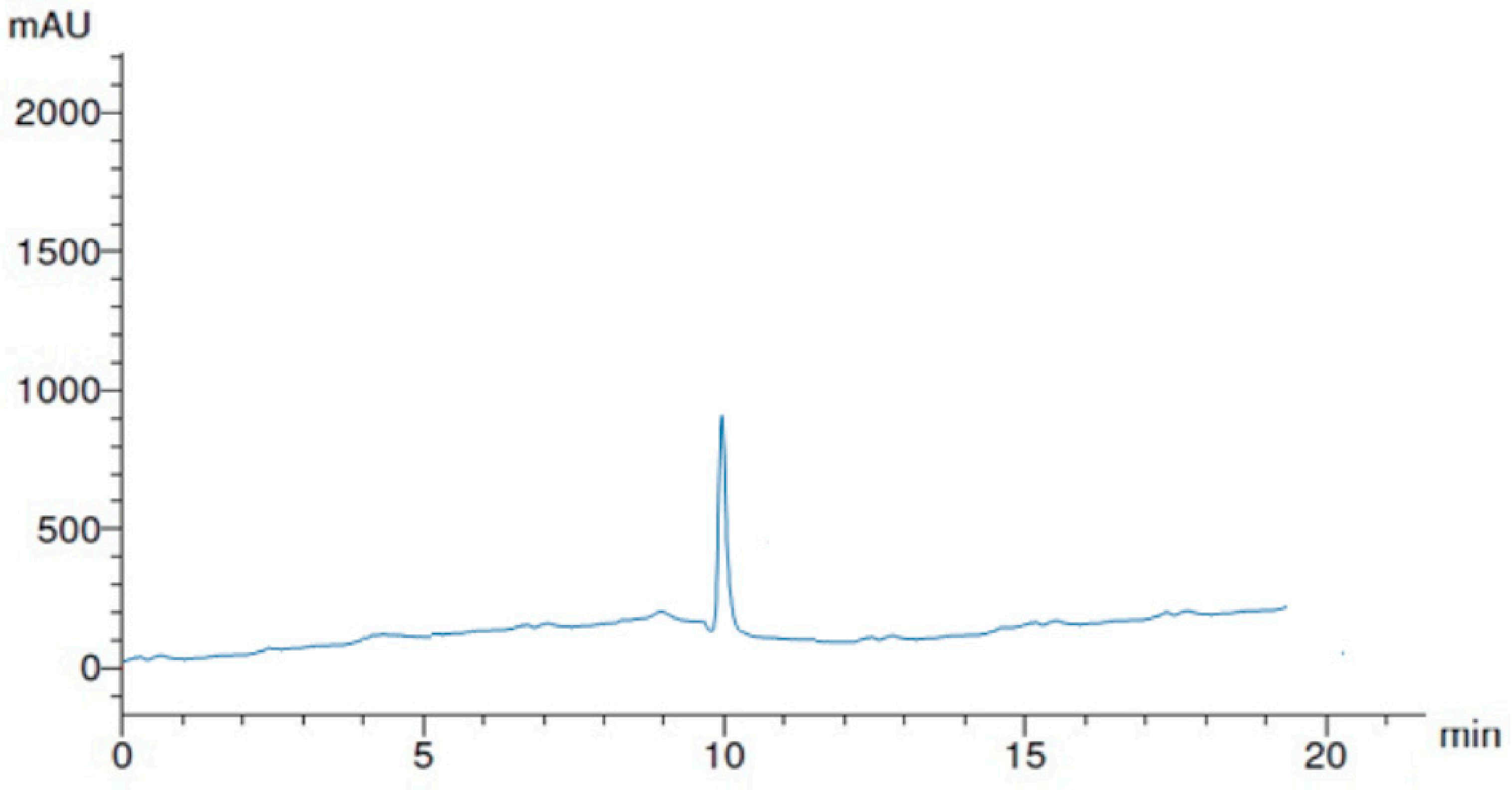

Ret. Time	Area (mAu)	Area %
10.0	78.155	100 %

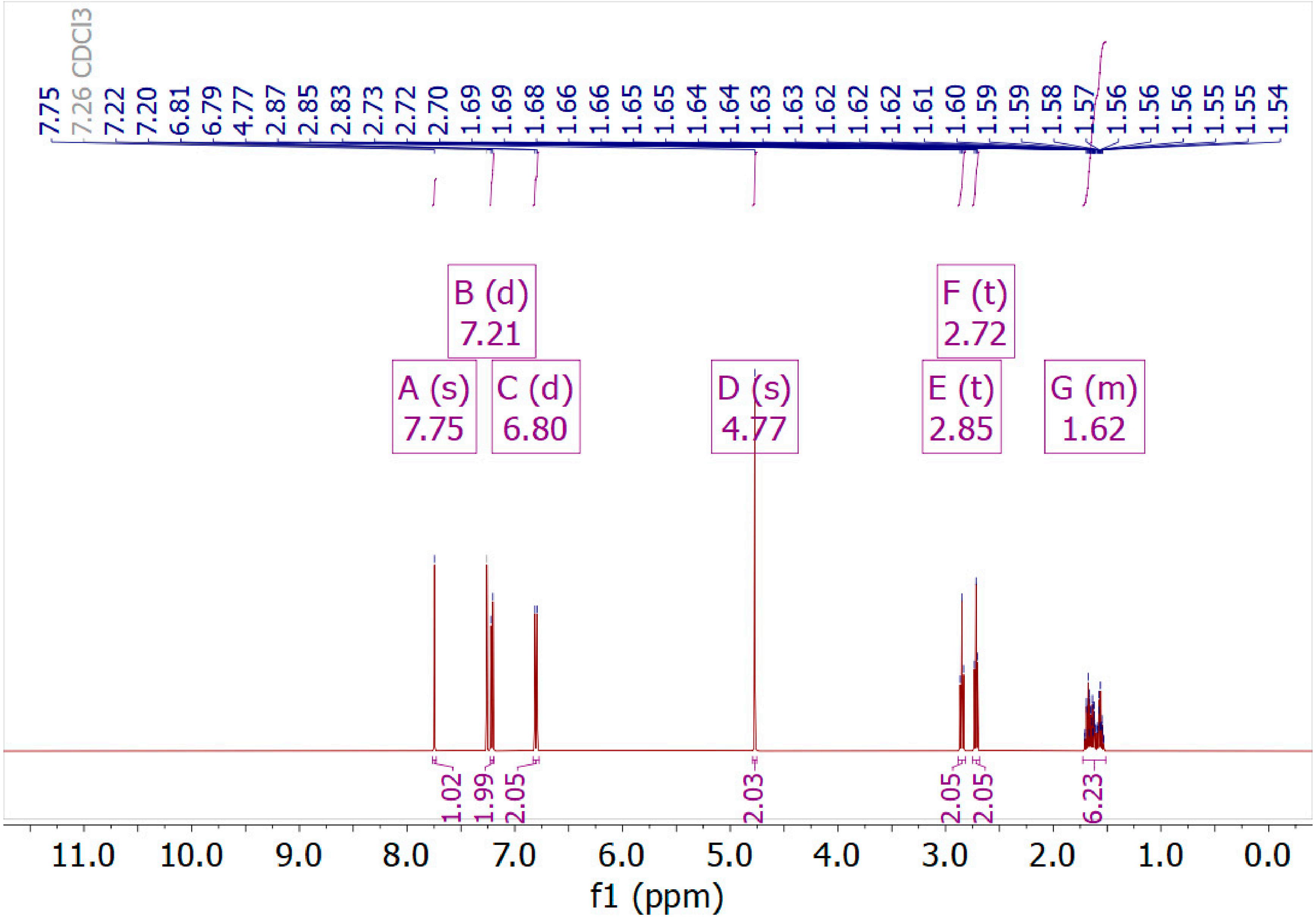
7.75 7.26 CDCl3 7.22 7.20 6.81 6.79 4.77 2.87 2.85 2.83 2.73 2.72 2.70 1.69 1.69 1.68 1.66 1.66 1.65 1.65 1.64 1.64 1.63 1.63 1.62 1.62 1.62 1.61 1.60 1.59 1.59 1.58 1.57 1.56 1.56 1.56 1.55 1.55 1.54
B (d) 7.21
F (t) 2.72
A (s) 7.75
C (d) 6.80
D (s) 4.77
E (t) 2.85
G (m) 1.62
1.02 1.99 2.05 2.03 2.05 2.05 6.23
11.0 10.0 9.0 8.0 7.0 6.0 5.0 4.0 3.0 2.0 1.0 0.0
f1 (ppm)

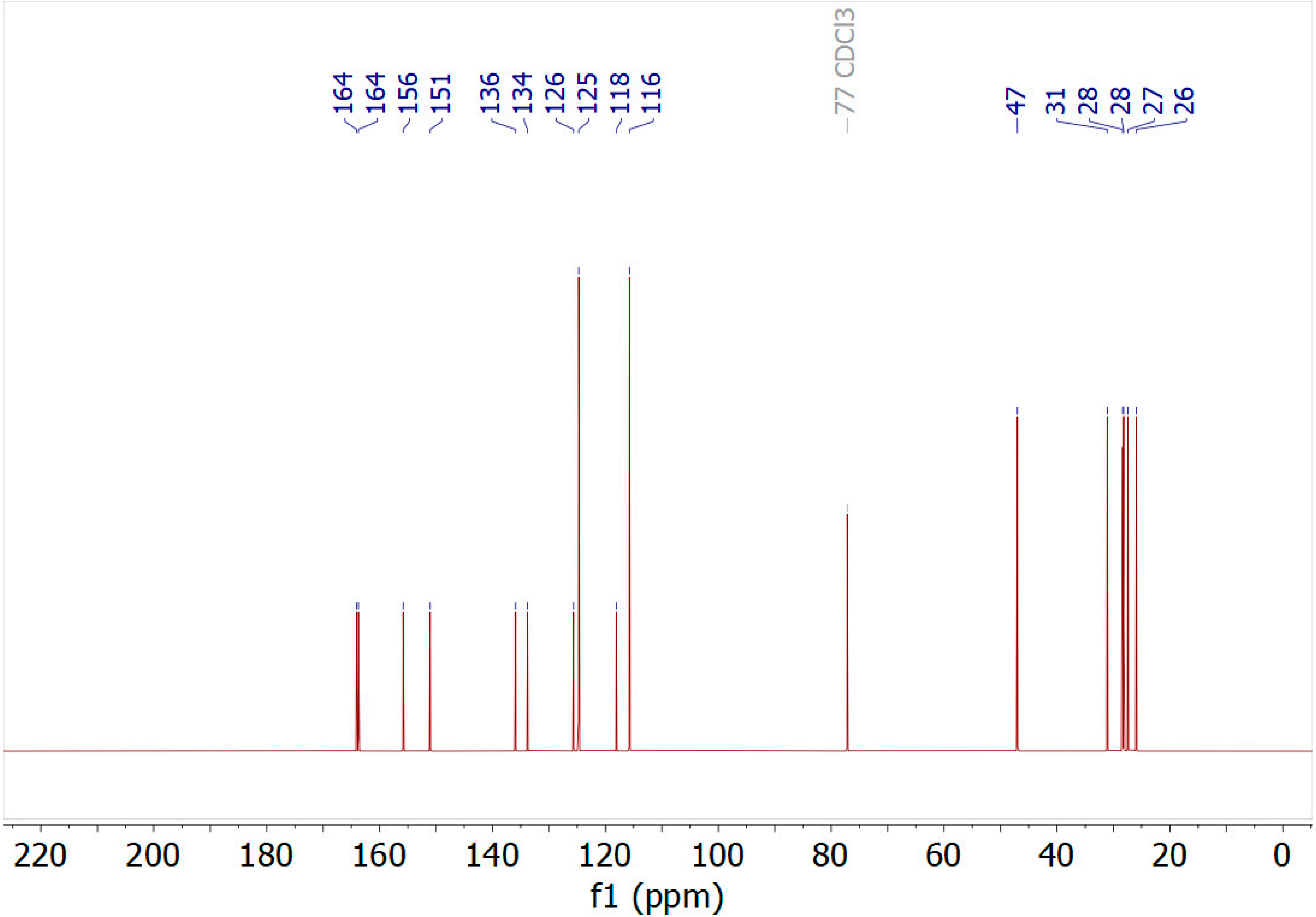
164
164
156
151
136
134
126
125
118
116
77 CDCl3
47
31
28
28
27
26
220 200 180 160 140 120 100 80 60 40 20 0
f1 (ppm)

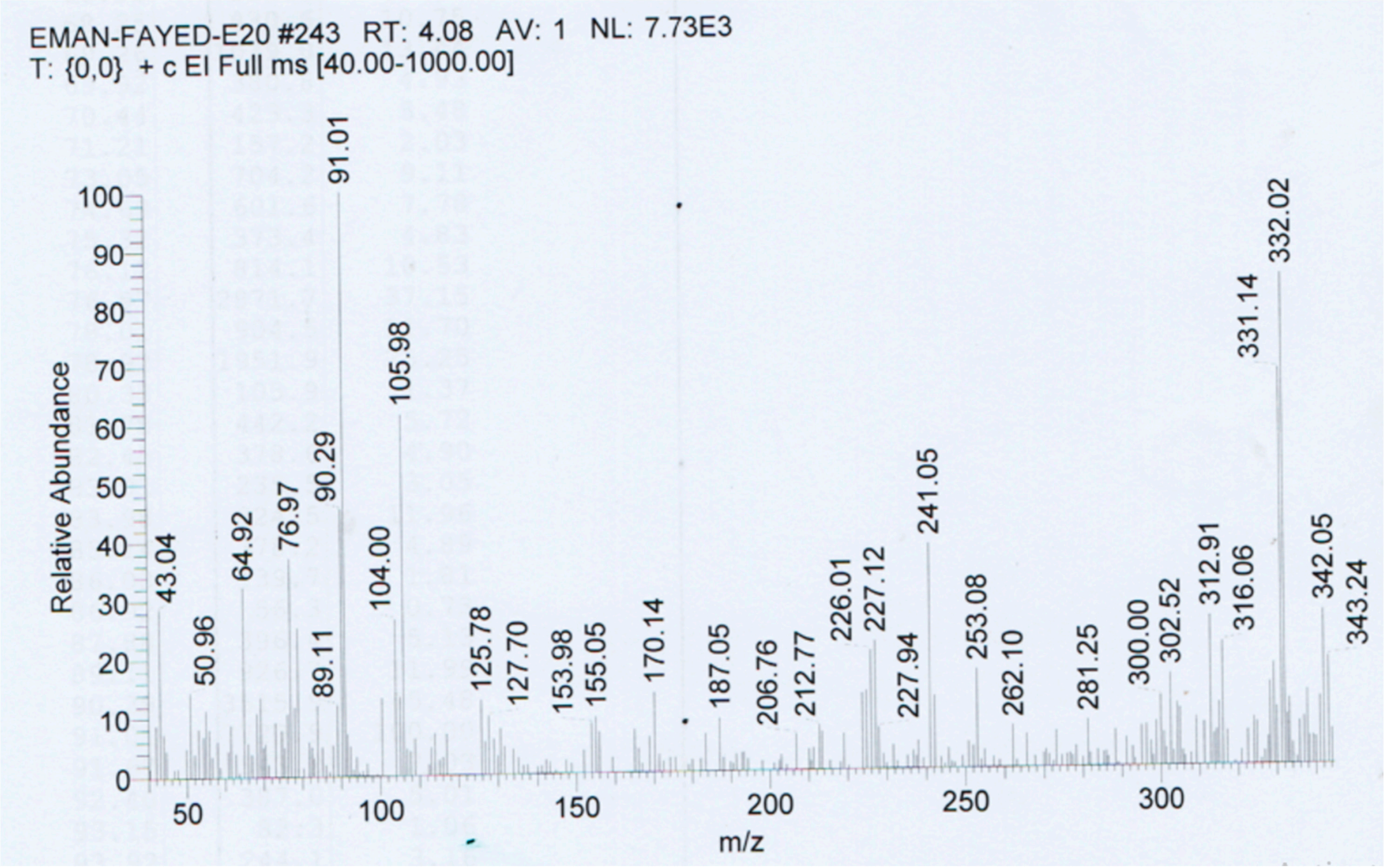
EMAN-FAYED-E20 #243 RT: 4.08 AV: 1 NL: 7.73E3
T: {0,0} + c EI Full ms [40.00-1000.00]
Relative Abundance
m/z
91.01
332.02
331.14
105.98
90.29
241.05
76.97
64.92
43.04
104.00
342.05
343.24
312.91
316.06
302.52
300.00
281.25
262.10
253.08
227.94
227.12
226.01
212.77
206.76
187.05
170.14
155.05
153.98
127.70
125.78
89.11
50.96

Compound 16

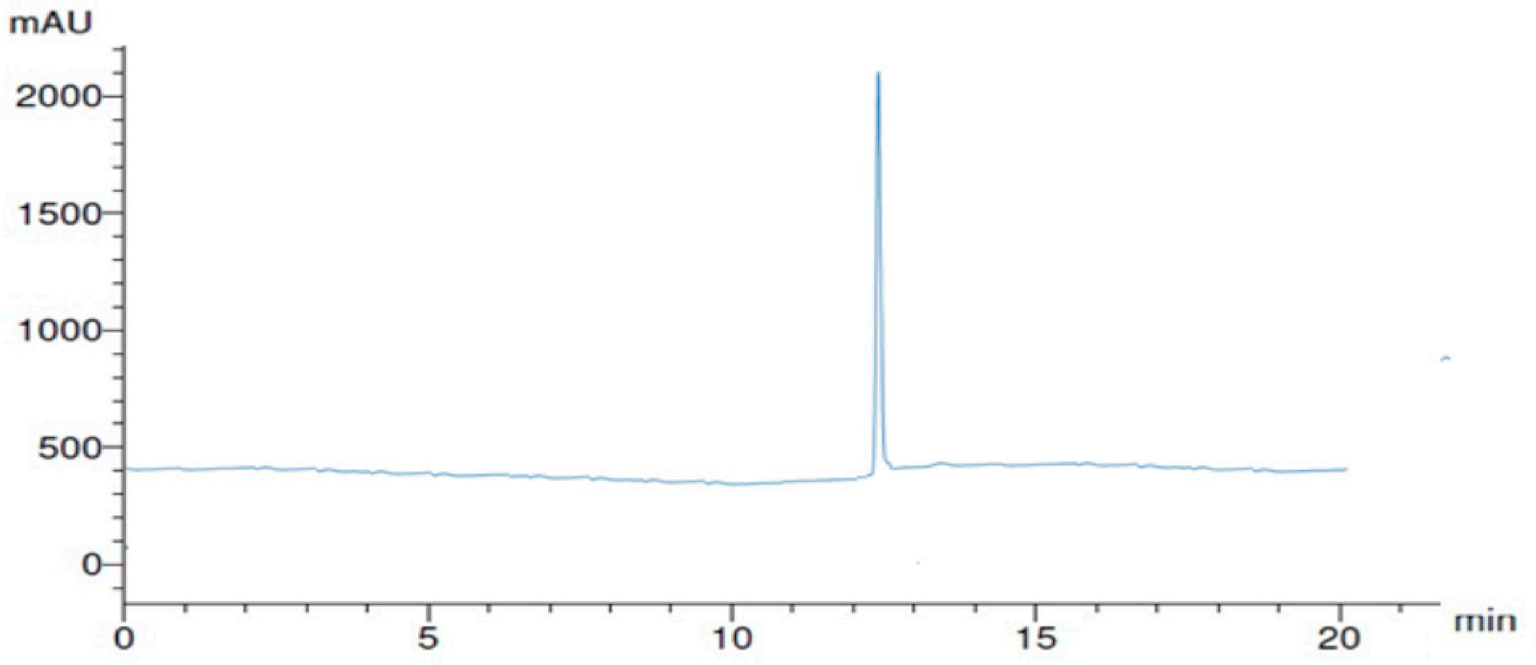

Ret. Time	Area (mAu)	Area %
12. 2	155. 652	100. 0

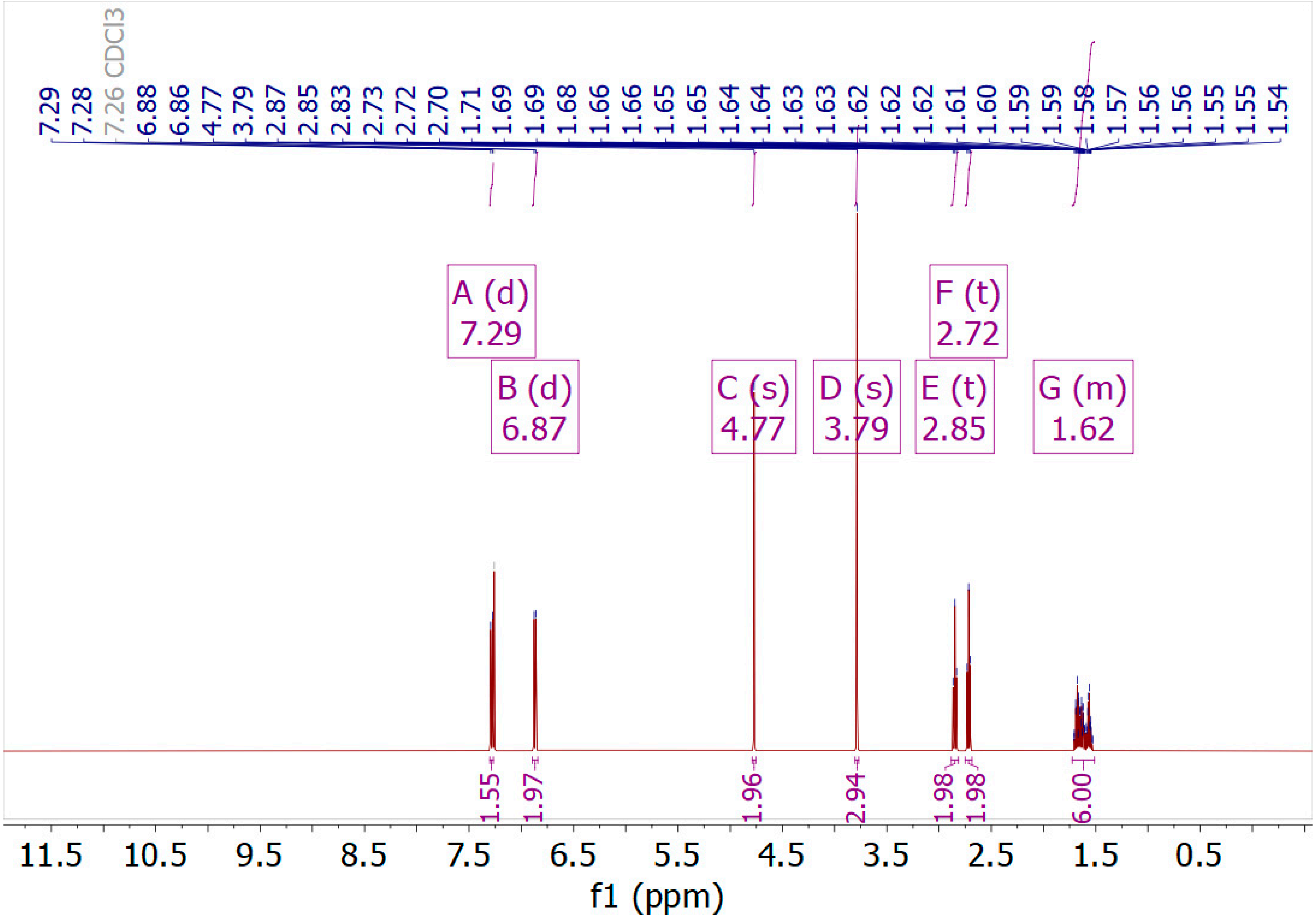

7.29
7.28
7.26 CDCl3
6.88
6.86
4.77
3.79
2.87
2.85
2.83
2.73
2.72
2.70
1.71
1.69
1.69
1.68
1.66
1.66
1.65
1.65
1.64
1.64
1.63
1.63
1.62
1.62
1.62
1.61
1.60
1.59
1.59
1.58
1.57
1.56
1.56
1.55
1.55
1.54
A (d) 7.29
B (d) 6.87
C (s) 4.77
D (s) 3.79
E (t) 2.85
F (t) 2.72
G (m) 1.62
1.55
1.97
1.96
2.94
1.98
1.98
6.00
11.5
10.5
9.5
8.5
7.5
6.5
5.5
4.5
3.5
2.5
1.5
0.5
f1 (ppm)

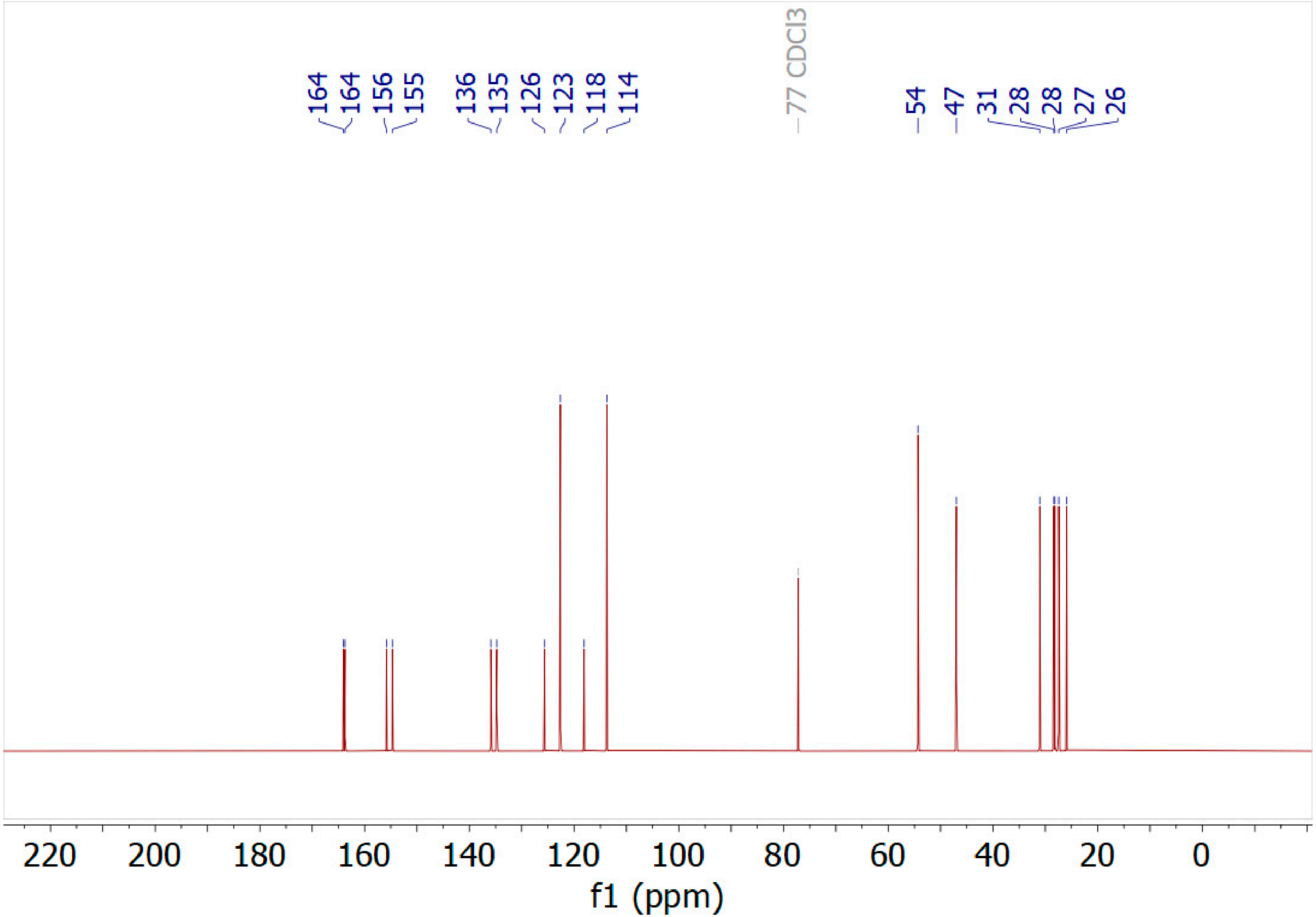

164
164
156
155
136
135
126
123
118
114
77 CDCl3
54
47
31
28
28
27
26
220 200 180 160 140 120 100 80 60 40 20 0
f1 (ppm)

Molecular Docking

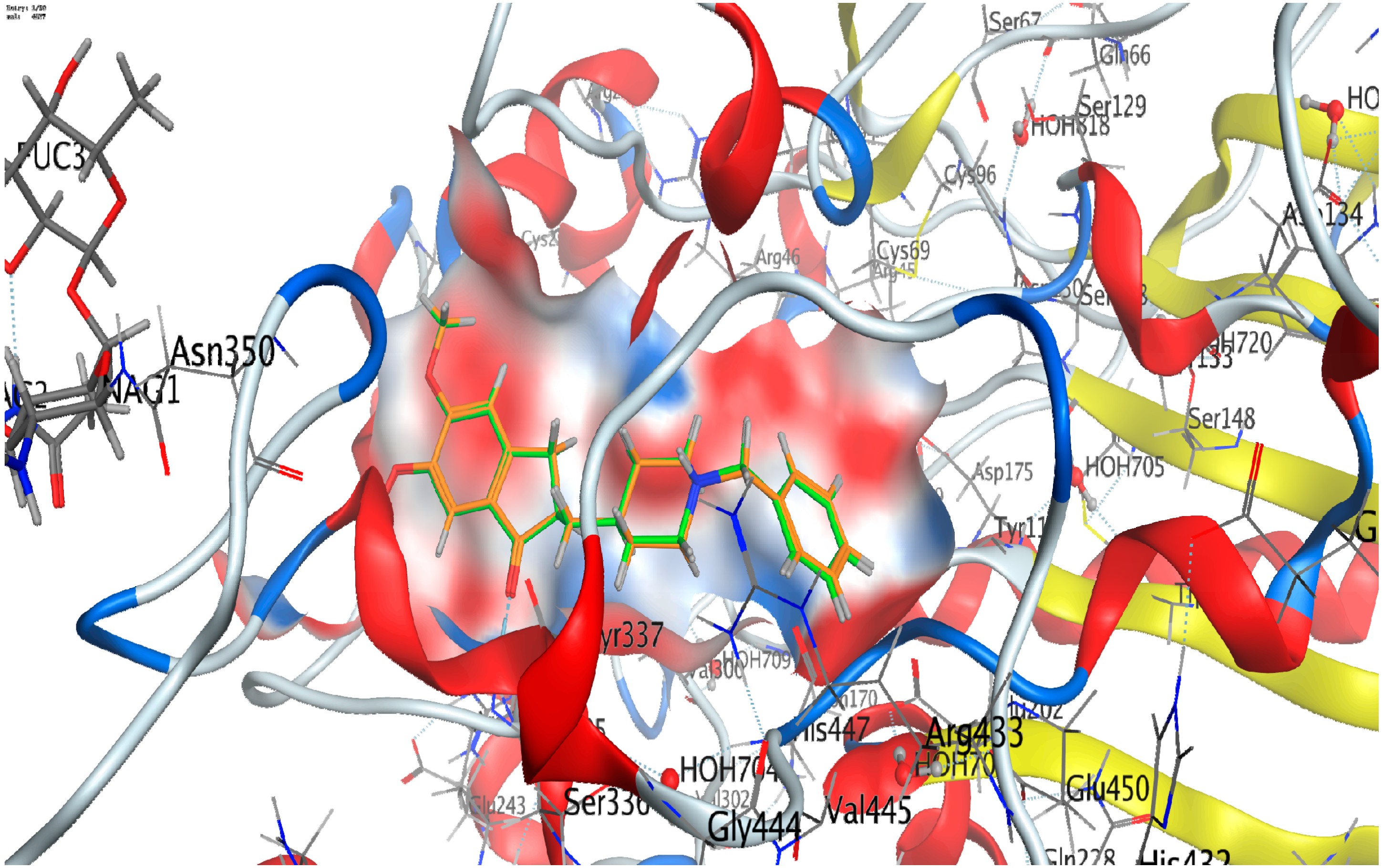

Figure S1: Overlay between co-crystallized ligand (green) and re-docked pose (orange) of Donepezil (RMSD= 0.117 Å)

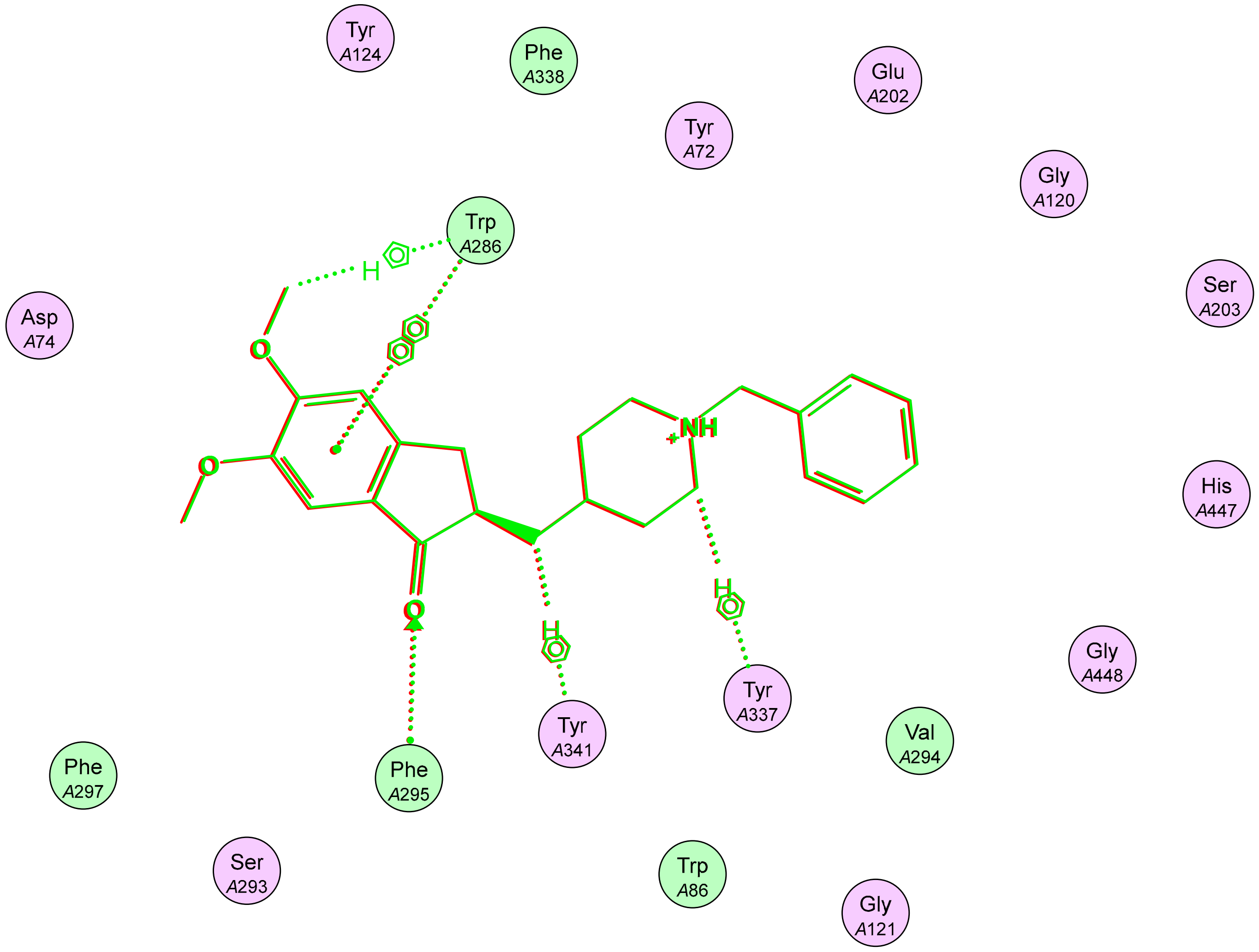

Figure S2: Ligand Interactions between the complex overlay and AChE pocket (PDB ID: 4EY7).

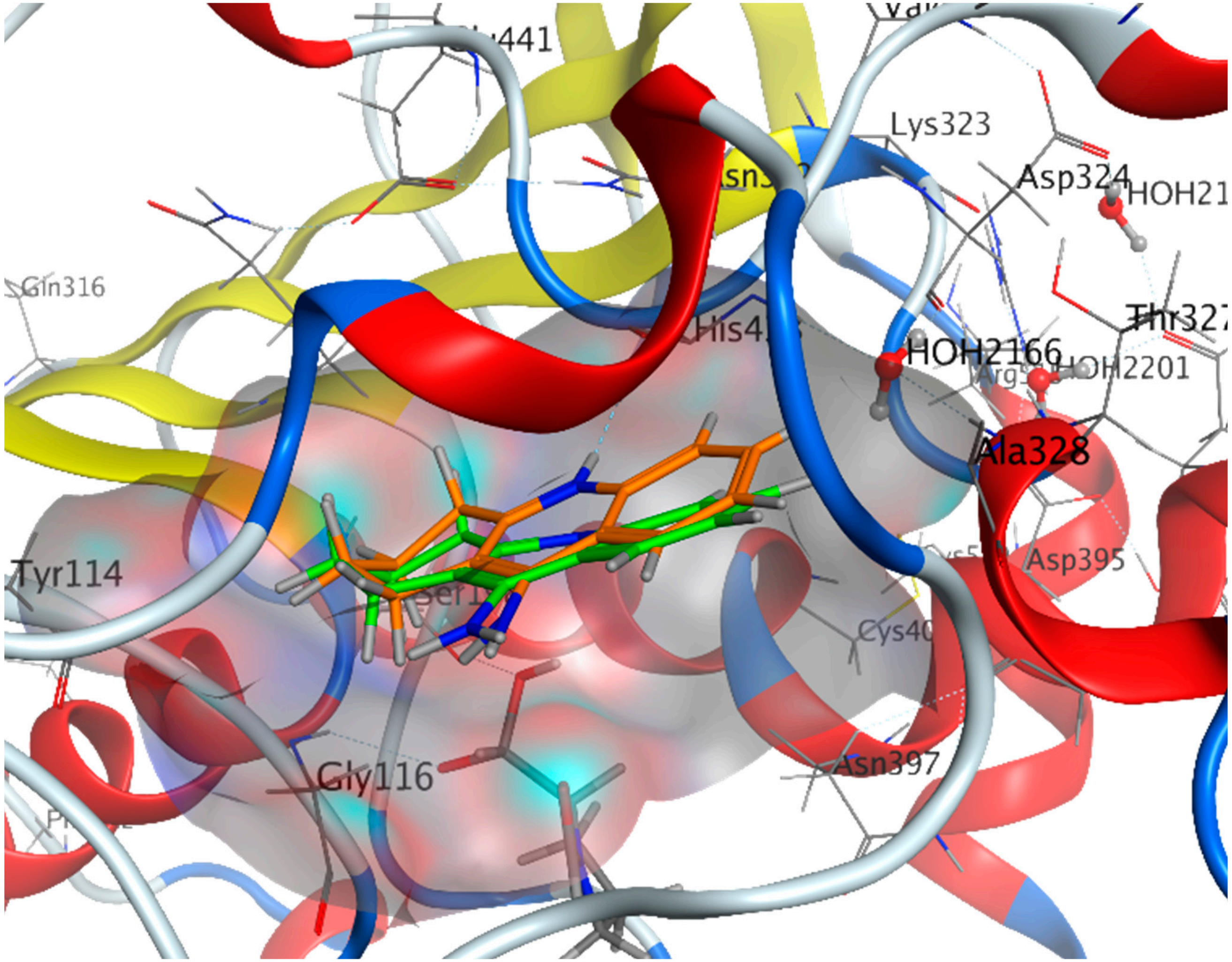

Figure S3: Overlay between co-crystallized ligand (green) and re-docked pose (orange) of Tacrine (RMSD= 0.589 Å).

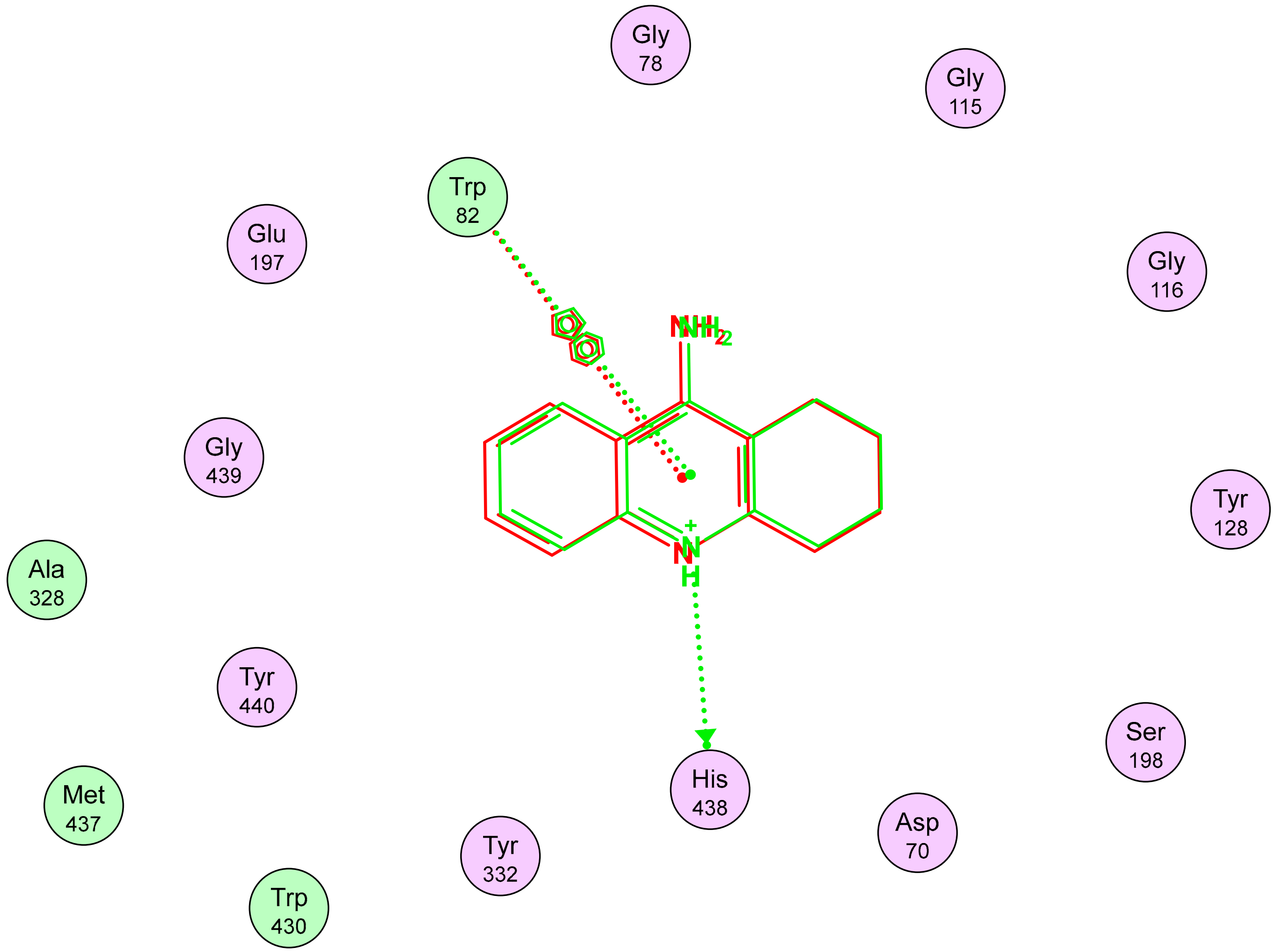

Figure S4: Ligand Interactions between the complex overlay and BChE pocket (PDB ID: 4BDS)

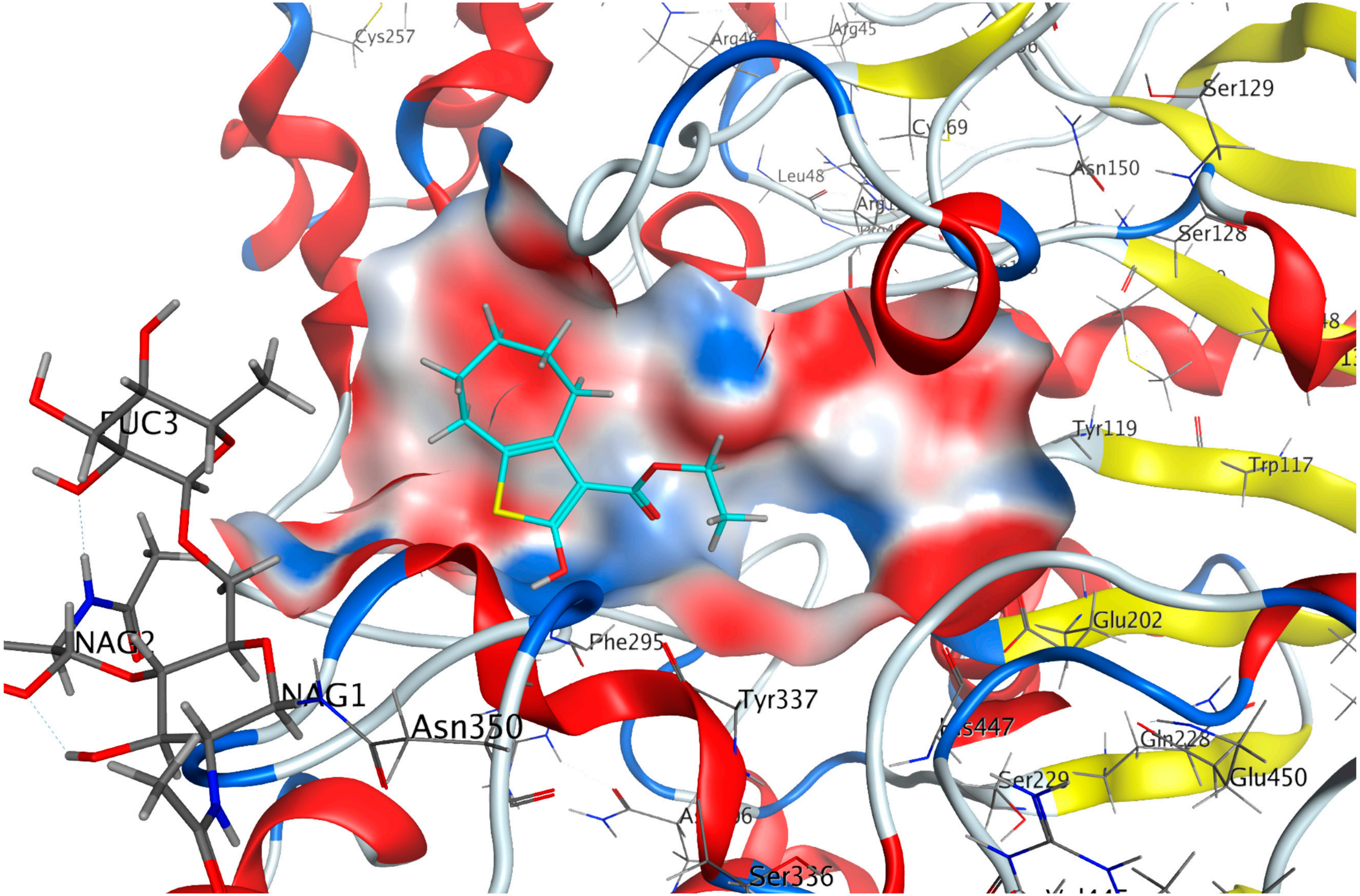

Figure S5: 3D binding modes of compound 2 with AChE (PDB ID: 4EY7).

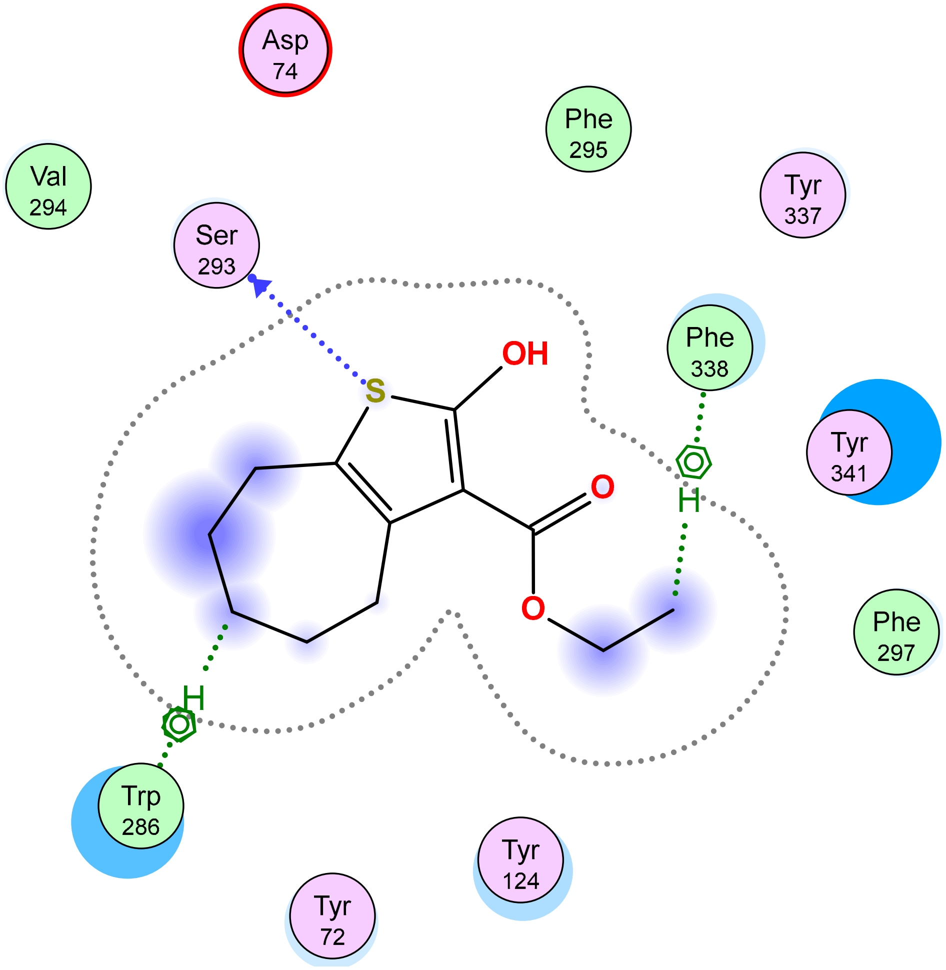

Figure S6: 2D ligand interactions of compound 2 with AChE (PDB ID: 4EY7).

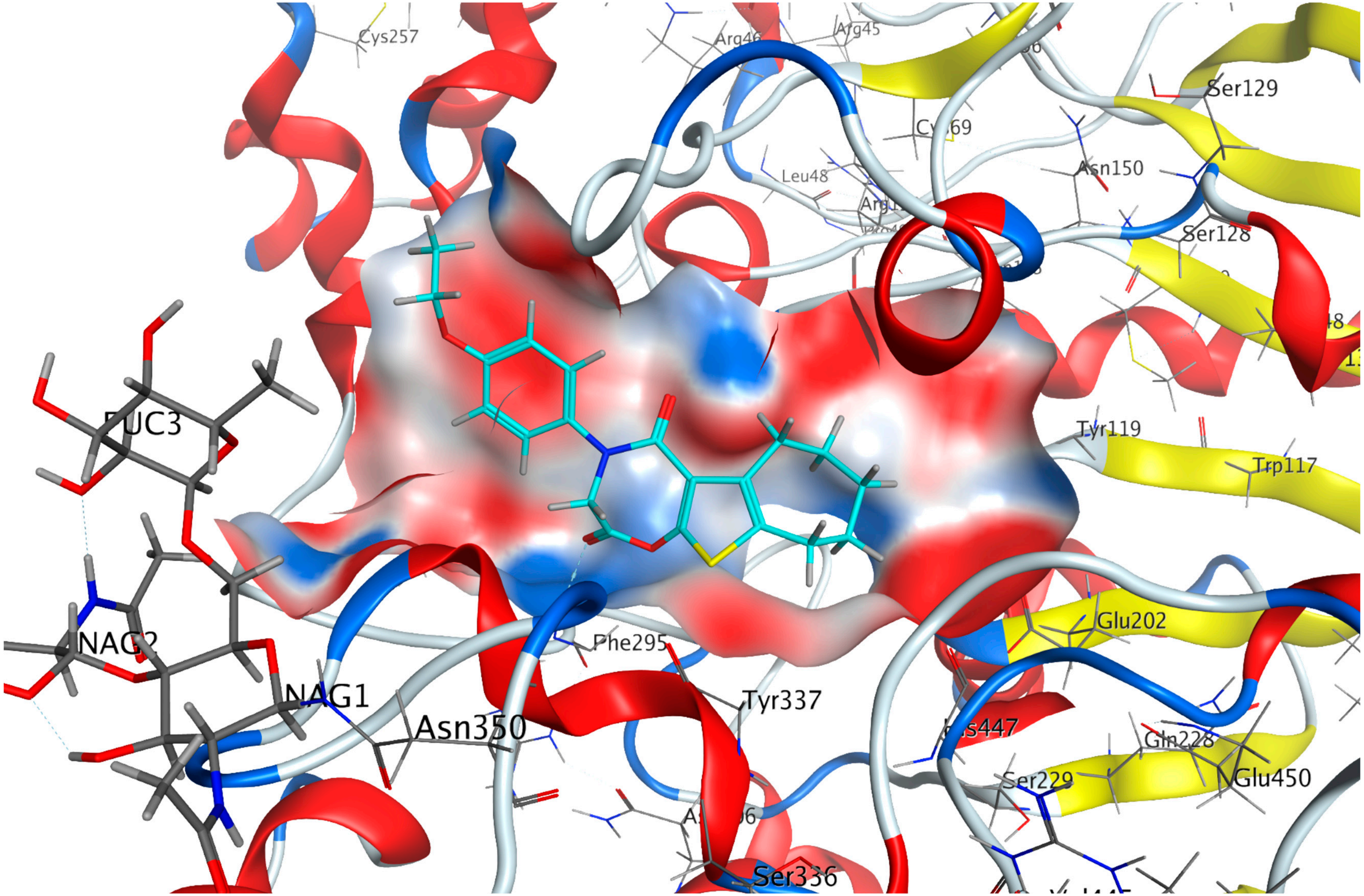

Figure S7: 3D binding mode of compound 14 with AChE (PDB ID: 4EY7)

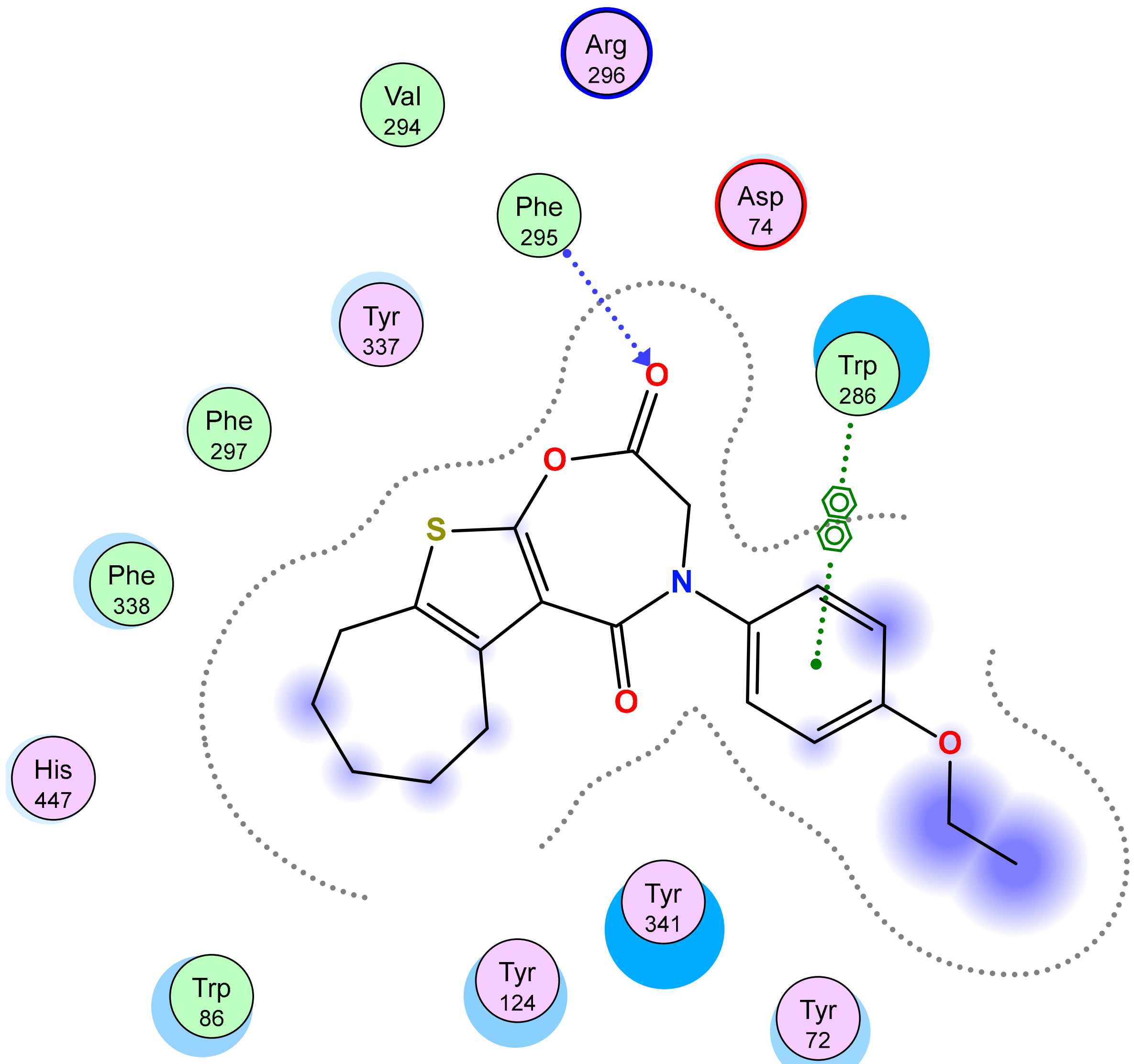

Figure S8: 2D ligand interactions of 14 with AChE (PDB ID: 4EY7)

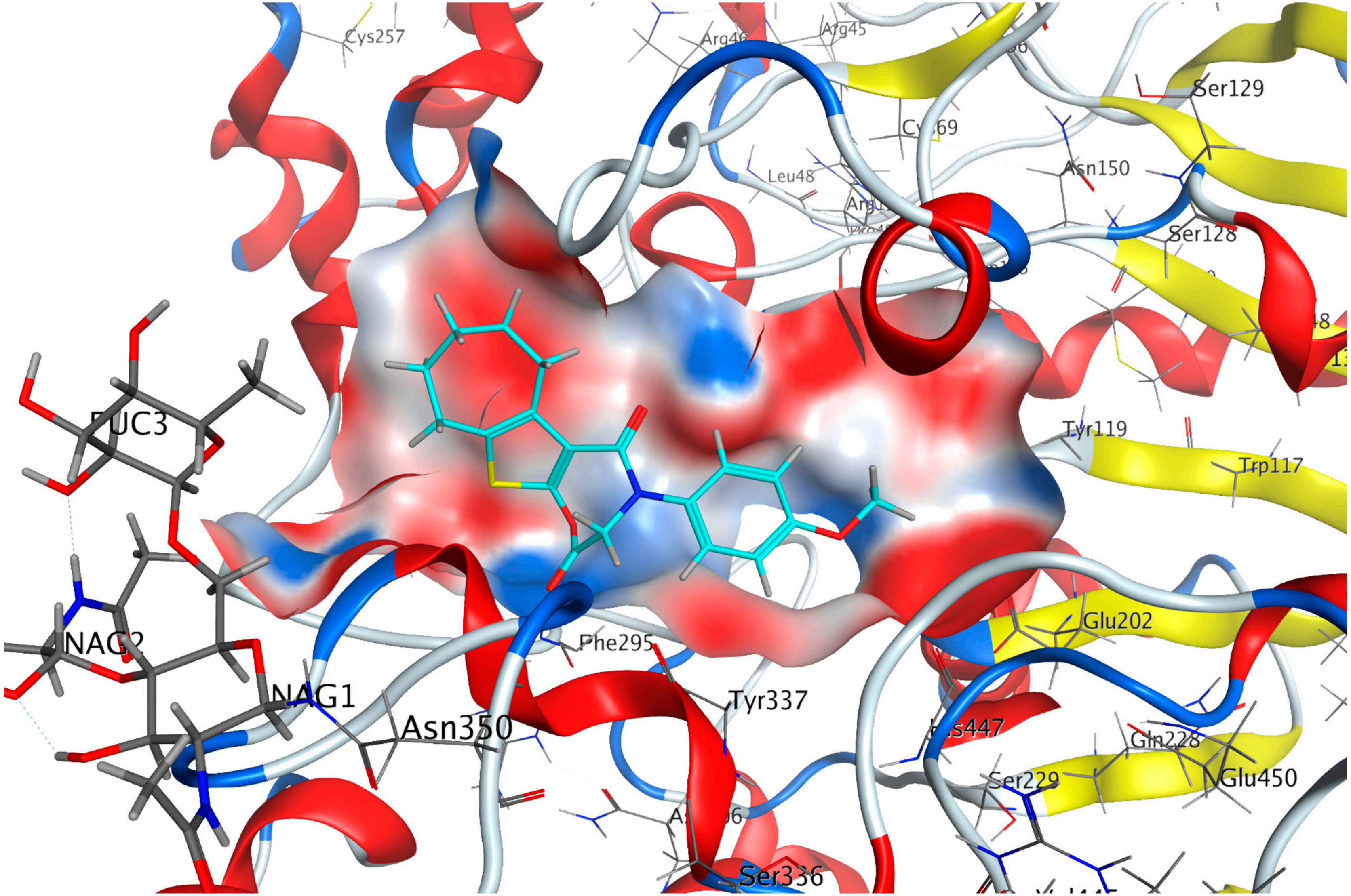

Figure S9: 3D binding mode of compound 16 with AChE (PDB ID: 4EY7).

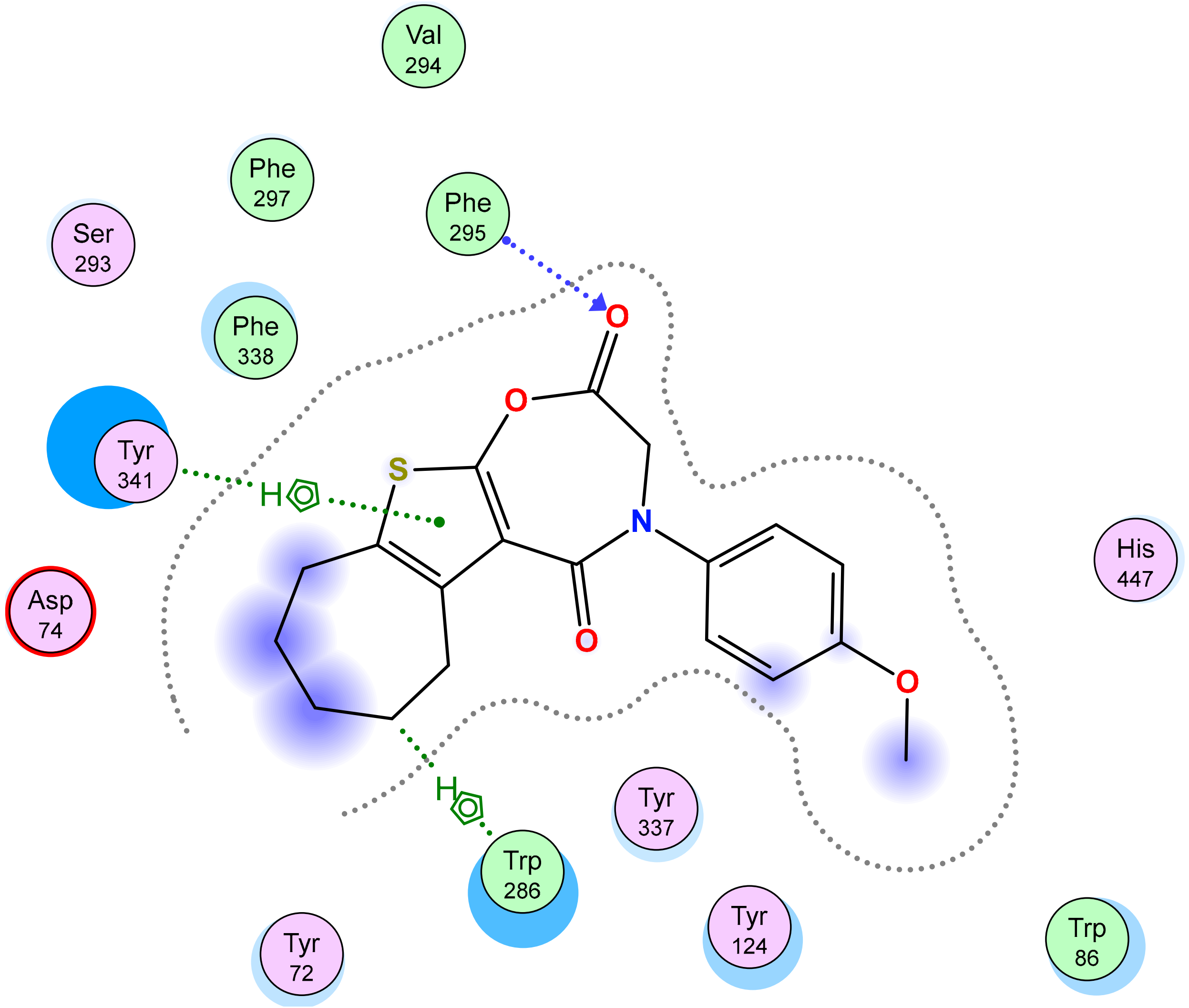

Figure S10: 2D ligand interactions of compound 16 with AChE (PDB ID: 4EY7).

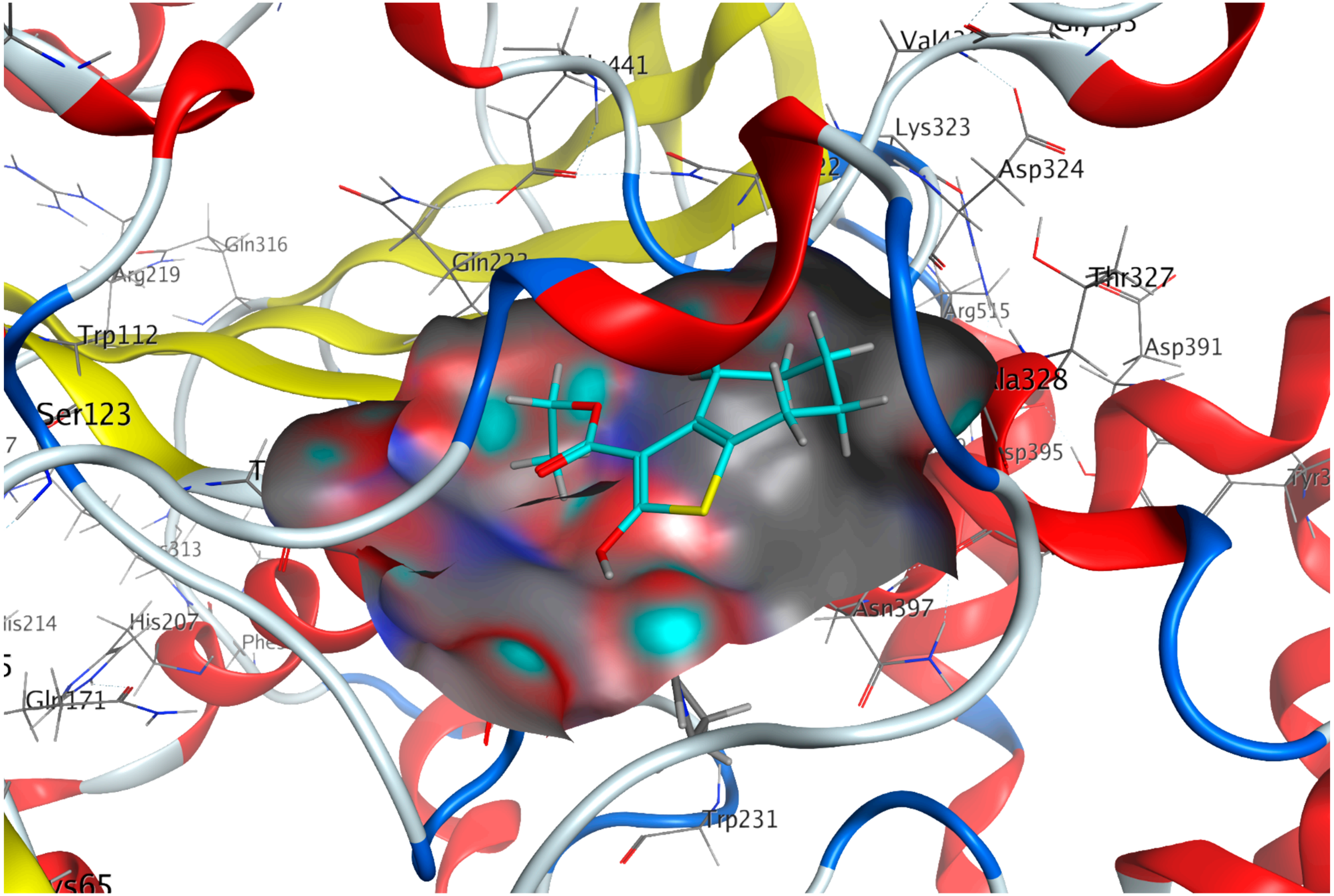

Figure S11: 3D binding mode of compound 2 with BChE (PDB ID: 4BDS)

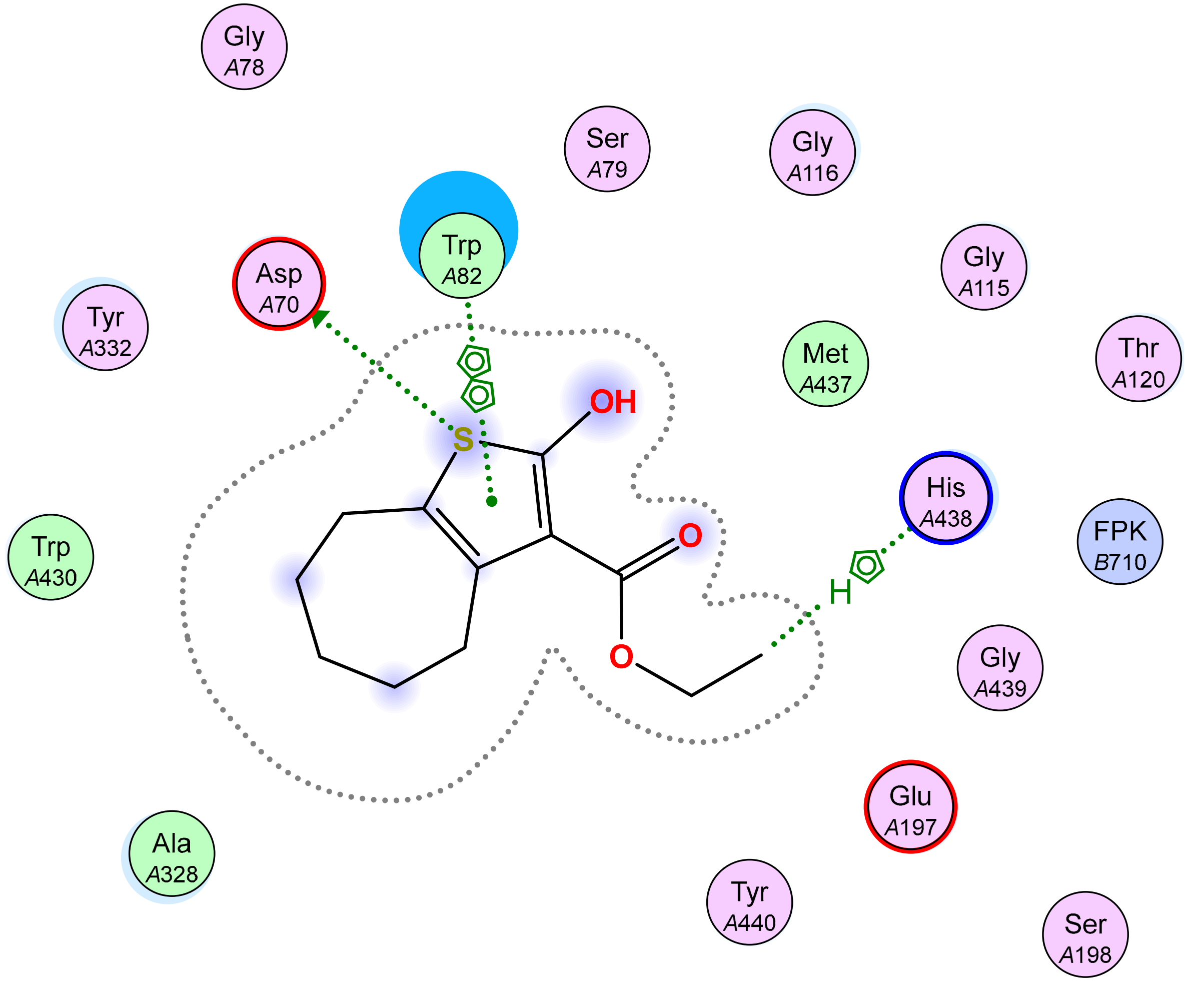

Figure S12: 2D ligand interactions of compound 2 with BChE (PDB ID: 4BDS)

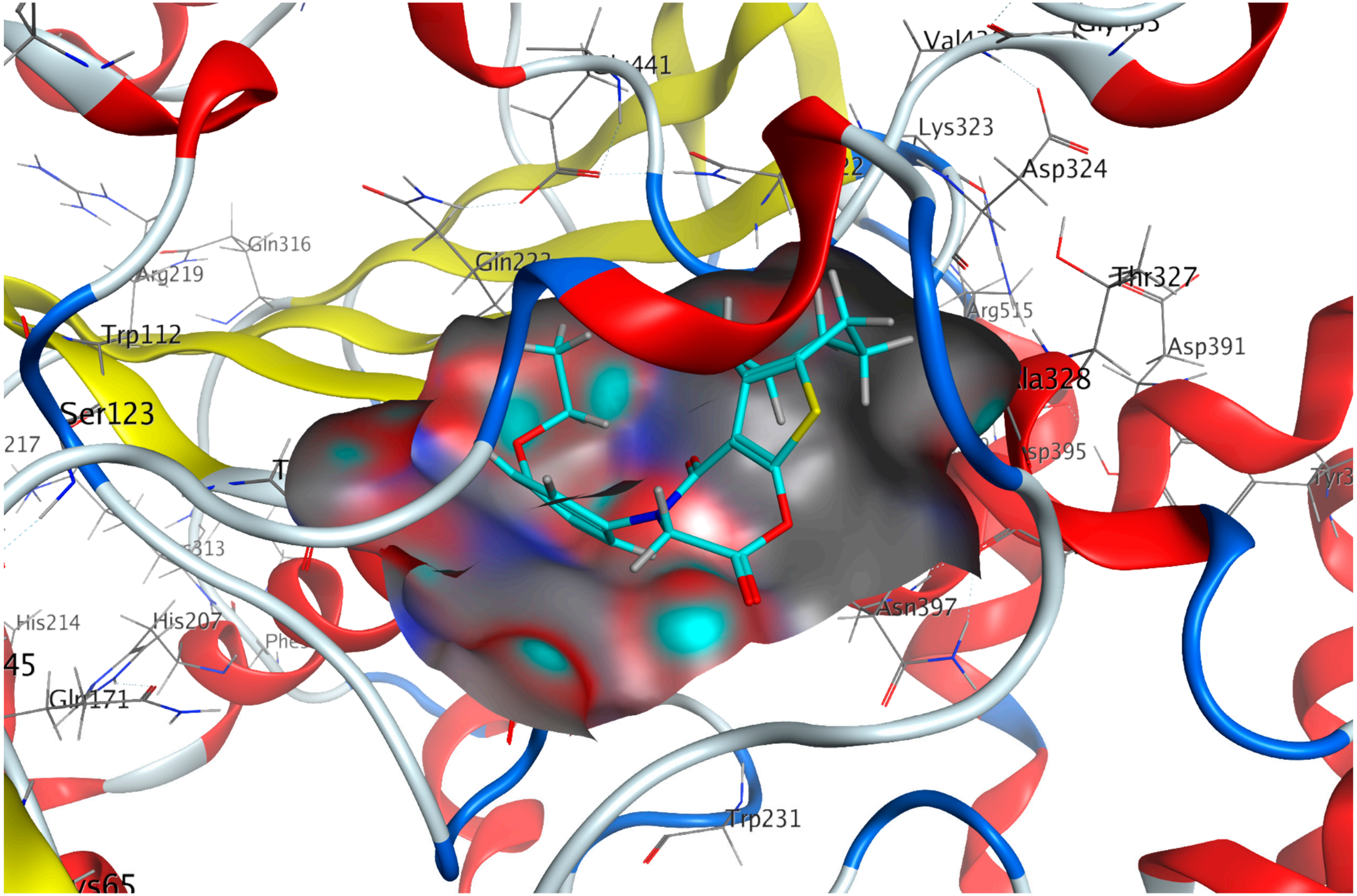

Figure S13: 3D binding mode of compound 14 with BChE (PDB ID: 4BDS)

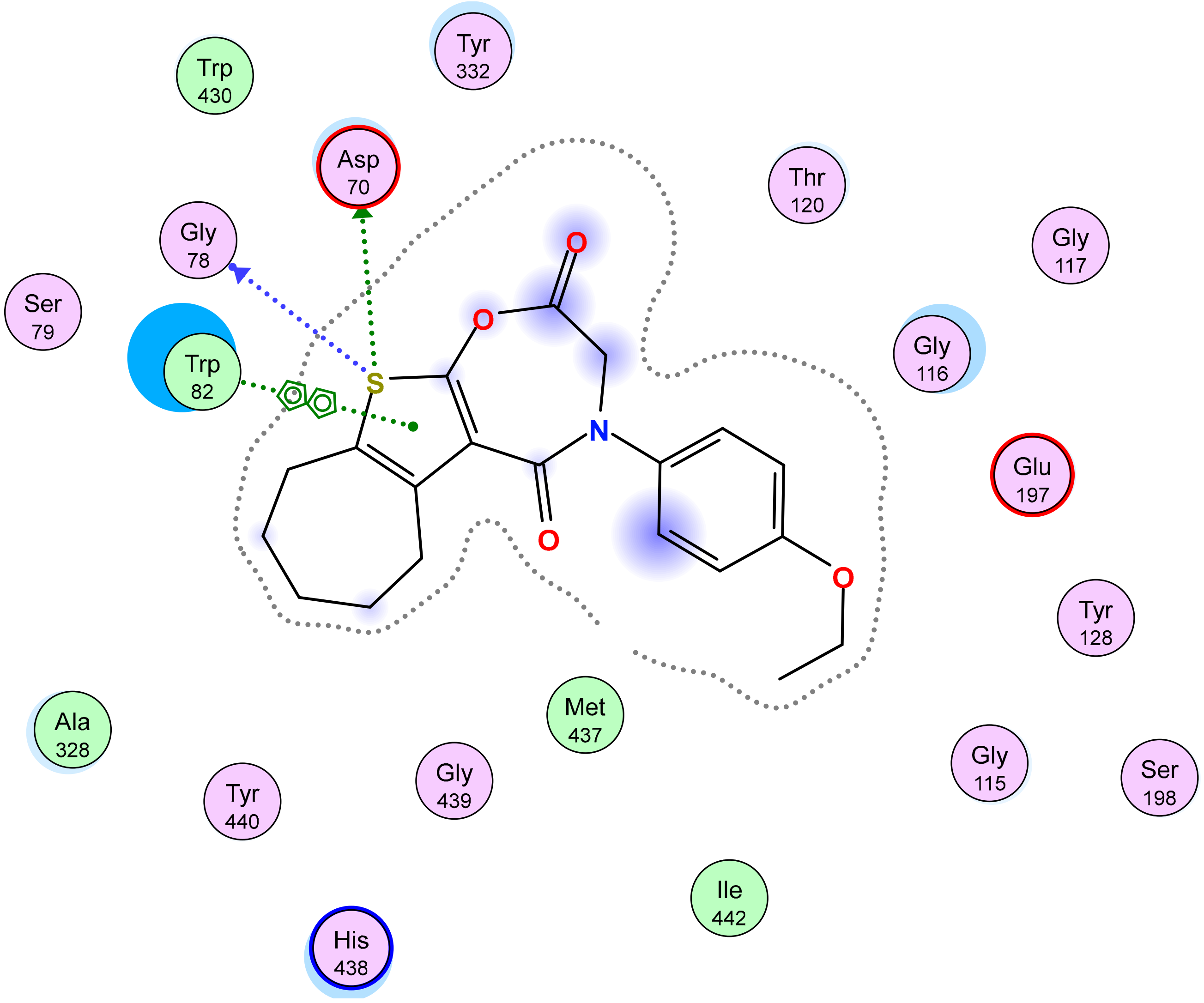

Figure S14: 2D ligand interactions of compound 14 with BChE (PDB ID: 4BDS)

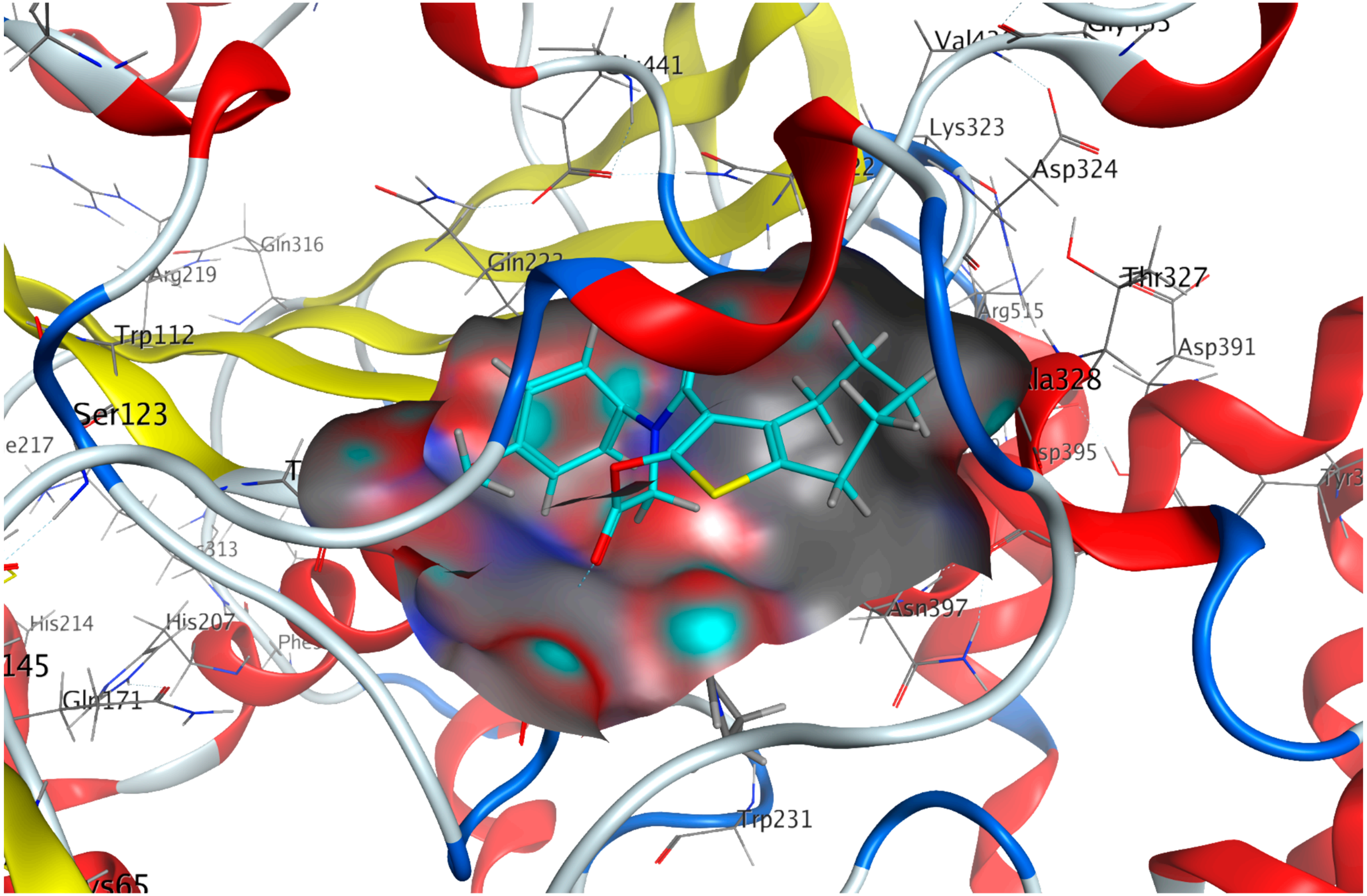

Figure S15: 3D binding mode of compound 16 with BChE (PDB ID: 4BDS)

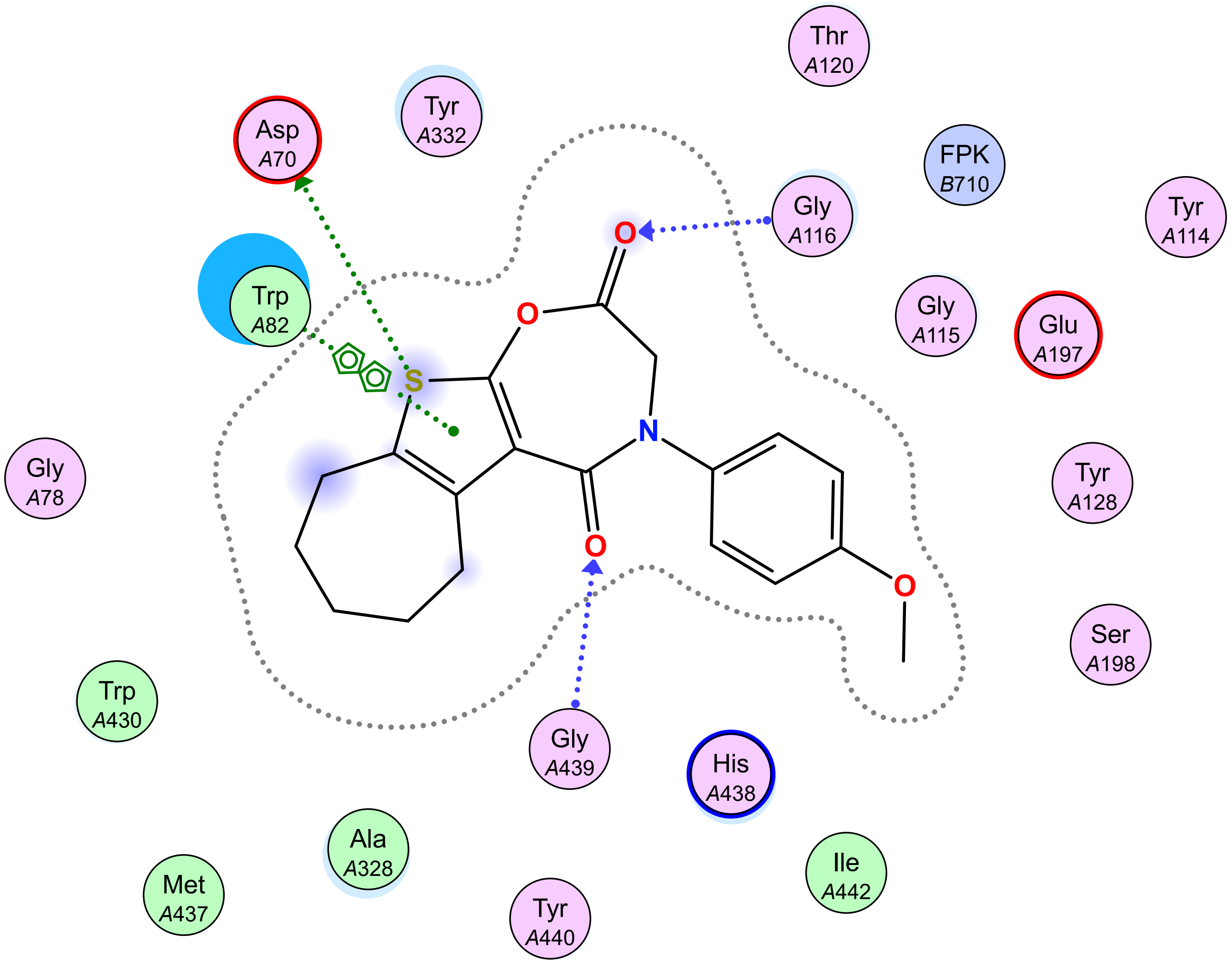

Figure S16: 2D ligand interactions of compound 16 with BChE (PDB ID: 4BDS)
